# Mapping the polysaccharide degradation potential of *Aspergillus niger*

**DOI:** 10.1186/1471-2164-13-313

**Published:** 2012-07-16

**Authors:** Mikael R Andersen, Malene Giese, Ronald P, Jens Nielsen

**Affiliations:** 1 Department of Systems Biology, Technical University of Denmark, Kgs. Lyngby, Denmark; 2 Fungal Physiology, CBS-KNAW Fungal Biodiversity Centre, Utrecht, The Netherlands; 3 Department of Chemical and Biological Engineering, Chalmers University of Technology, Gothenburg, Sweden

## Abstract

**Background:**

The degradation of plant materials by enzymes is an industry of increasing importance. For sustainable production of second generation biofuels and other products of industrial biotechnology, efficient degradation of non-edible plant polysaccharides such as hemicellulose is required. For each type of hemicellulose, a complex mixture of enzymes is required for complete conversion to fermentable monosaccharides. In plant-biomass degrading fungi, these enzymes are regulated and released by complex regulatory structures. In this study, we present a methodology for evaluating the potential of a given fungus for polysaccharide degradation.

**Results:**

Through the compilation of information from 203 articles, we have systematized knowledge on the structure and degradation of 16 major types of plant polysaccharides to form a graphical overview. As a case example, we have combined this with a list of 188 genes coding for carbohydrate-active enzymes from *Aspergillus niger*, thus forming an analysis framework, which can be queried. Combination of this information network with gene expression analysis on mono- and polysaccharide substrates has allowed elucidation of concerted gene expression from this organism. One such example is the identification of a full set of extracellular polysaccharide-acting genes for the degradation of oat spelt xylan.

**Conclusions:**

The mapping of plant polysaccharide structures along with the corresponding enzymatic activities is a powerful framework for expression analysis of carbohydrate-active enzymes. Applying this network-based approach, we provide the first genome-scale characterization of all genes coding for carbohydrate-active enzymes identified in *A. niger*.

## Background

Expression profiling of the genes encoding extracellular enzymes is of high relevance to several industries. Commercial enzyme-preparation are often targeted to a specific activity such as cellulase for the degradation of cellulose and preparation of fabrics, or amylases for the preparation of dense syrups from starch or similar compounds. The world market for industrial enzymes is a multi-billion dollar market [1]. Another emerging market is the production of second generation biofuels. Energy-efficient processes rely on enzymes produced by fungi and other degraders of dead biomass (saprobes) to produce cheap CO_2_-neutral fuels from non-edible plant matter [2]. For this study, we have chosen to study the saprobic fungus *Aspergillus niger*, a well studied, efficient, and widely used enzyme producer [3,4].

The interest in profiling the expression patterns of genes coding for polysaccharide-active enzyme is not new, but few large-scale surveys have been done. An early study was made by one of the authors [5] using Northern blotting to create expression profiles of 26 pectinolytic genes under 16 different growth conditions. The publication of the first *A. niger* genome [3] provided the prediction of 171 polysaccharide-active enzymes, along with microarrays for expression profiling. This has catalyzed more research, specifically the work of Martens-Uzunova [6], where expression profiles of 21 pectinolytic genes are examined. Other studies by Yuan et al. [7-9], examined the degradation of the polysaccharide inulin and identified the regulating protein and, in one case, performed expression profiling of genes from three out of the 36 carbohydrate-acting enzyme families predicted by Pel et al. [3] to be present in *A. niger*. While studies of this type address interesting parts of the polysaccharide degradation potential, there is a need to evaluate the entire scope of polysaccharides and enzymes, to fully address the challenges involved in complete degradation of plant biomass for biotechnology purposes.

The degradation of plant biomass is a complex procedure requiring a cocktail of enzymes. Furthermore, the polysaccharides are rarely found independently in nature. hemicellulose, found in the cell walls of plants, are a complex three-dimensional structure of several types and structures of polysaccharides such as glucomannan, arabinan, and xylan. It has been shown in several studies [10-12], that the presence of one simple saccharide can trigger the expression of enzymes for the degradation of an entirely different structure (as has been shown to be the case with D-xylose [13]). Another possibility with industrial applications is the use of a cheap carbon-source to induce the enzymes required for degradation of a more complex and expensive one [12,14]. One example is the work of Yuan et al. [7], where sucrose induces the genes for all of the enzymes required for degrading inulin.

With the complex regulation of expression of a large number of different enzymes and the fact that many different enzymes are needed for the utilization of certain carbon sources (see e.g. ref. [15]), it is necessary to apply a systems-wide approach for mapping the regulatory network. The enzyme expression network can be combined and cross-triggered, so being able to examine the entire system at once can shed light on systems that might not be possible to pre-empt with a hypothesis-driven approach.

In this study, we have compiled knowledge on the structure and degradation of 16 types of plant polysaccharides from ¿200 articles. This has been combined with a list of 188 genes from *A. niger* coding for carbohydrate-active enzymes [3] to form a systematic graphical overview.

This makes it possible to highlight directly on the polysaccharide structures e.g. which genes are actively induced on a specific carbon source. This is a network-based approach for interpretation of data, where the network is provided by the structures of the extracellular polysaccharides, in contrast to the intracellular metabolic or regulatory networks often used for data-interpretation.

We apply this reconstructed network to investigate how enzymes interact to degrade complex polysaccharides with applications within sustainable biotechnology. The correlation of gene expression analysis on three monosaccharides and three complex carbohydrates with the network allowed the detection of concerted enzymatic actions as well as cross-induction of enzymatic cocktails.

We also see the combination of the network mapping of available information on the structure of polysaccharides with the transcription analysis as a source of reference for researchers interested in the induction of specific carbohydrate-active genes on certain substrates.

## Results and discussion

### Polysaccharide mapping generates a graphical knowledge base on biomass degradation

In a review of the available literature on the degradation of polysaccharides by *A. niger*, information was compiled on the following 10 types of polysaccharides: starch, cellulose, pullulan, inulin, galactomannan, galactoglucomannan (soluble and insoluble), xyloglucan (types XXGG and XXXG), as well as the following six distinct components of pectin: smooth pectin (pectate), xylogalacturonan, arabinogalactan (type I and II, also known as protein-bound arabinogalactan), arabinan, and rhamnogalacturonan I. The last known polysaccharide component of pectin, rhamnogalacturonan II, was not included even though the structure has been elucidated [16], since no studies of its degradation by *A. niger* were found in the literature search. Analyses of the degradation of this polysaccharide is made more difficult by the fact that it is composed mainly of highly modified and rare sugars and thought to be the most complex polysaccharide on Earth [16].

**Figure 1:**
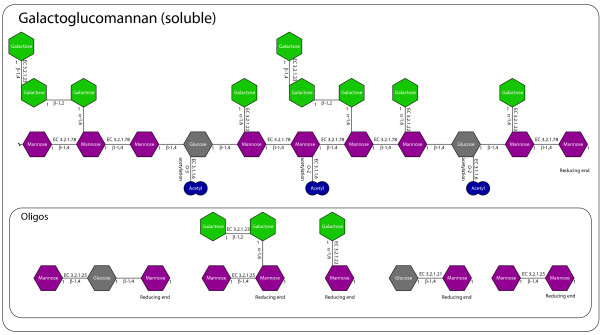
**Schematic representation of soluble galactoglucomannan.** The nature of the bonds between the sugar units are noted where they are known. The number of sides on the sugar polygons reflect the number of carbons of the sugar. The first carbon is indicated on all sugars with a 1 to clarify the bond configurations. Carbons are numbered clockwise from the first carbon with the two last carbons of the sugar (hexose or pentose) on the same corner of the polygon. The oligos are hypothetical hydrolysis products and sugars that appear due to the action of exo-acting enzymes. The structure is based on the reportings of refs. [18-21].

Sixteen structures have been gathered in schematic representations of each type of polysaccharide. An example of this (for soluble galactoglucomannan) is found in Figure 1. Schematic representations for all 16 structures and information on the genes are found in Additional files 1, 2, 3, 4, 5, 6, 7, 8, 9, 10, 11, 12, 13, 14, 15 and 16.

For each of these structures, the available literature and sequence databases (Swissprot/Uniprot http://www.expasy.org/sprot/) were examined and information was gathered on the enzymes required for their degradation. This was integrated on the schematic representation of the structure as EC numbers where available, or as enzyme-names if the EC number was not available (As demonstrated in Figure 1). This was compared to the annotation of *A. niger* CBS 513.88 genome sequence [3] to identify putative isoenzymes for each activity. For each structure, this has resulted in a table containing the activities required for degradation, reference to literature on the characterization of the genes and enzymes, as well as information on the specificities where available. The sequence information is found as Uniprot accession numbers as well as gene IDs in the *A. niger* CBS 513.88 and ATCC 1015 sequence. Further information was found in an analysis of the carbohydrate degradation genes from *A. nidulans*[17]. An example of this is found in Table 1, with the full table and tables for all 16 structures found in Additional file 17. This annotation is a valuable reference on carbohydrate degradation by *A. niger*. The combination of structures and specifics on required enzymatic activities and genes makes this a significant extension of previous studies, which are dealing with a subset of the structures and generic enzymatic activities [15].

**Table 1:** **Extract of Additional file **17**: Table S6 of the enzymatic activities required for the degradation of soluble galactoglucomannan by *A. niger* **


**EC number**	**Name**	**CAZy**	**Gene characterization**	**Specificity**	**Gene**	**Uniprot**	**CBS 513.88**	**ATCC 1015**
3.2.1.78	Endo-1,4-*β*-D-mannanase	GH5	[22]	[22]			An05g01320	50378
3.2.1.78	Endo-1,4-*β*-D-mannanase	GH26					An15g07760	40875
3.2.1.25	*β*-mannosidase	GH2					An01g06630	172587
3.2.1.25	*β*-mannosidase	GH2	[23-25]	[23,24]	**mndA**	Q9UUZ3	An11g06540	138876
3.2.1.22	*α*-galactosidase	GH36	[24,26]	[24]	**aglC**	Q9UUZ4	An09g00260	212736
3.2.1.22	*α*-galactosidase	GH27					An01g01320	172232
3.2.1.22	*α*-galactosidase	GH27	[26,27]		**aglB**	Q9Y865	An02g11150	207264
3.2.1.22	*α*-galactosidase	GH27	[26,28,29]		**aglA**	A2QL72	An06g00170	37736
3.2.1.21	*β*-glucosidase	GH3					An15g04800	181816
3.2.1.21	*β*-glucosidase	GH3					An17g00520	129891
3.2.1.21	*β*-glucosidase	GH3	[30-36]		**bgl1**	A2RAL4	An18g03570	56782
3.2.1.23	*β*-galactosidase	GH35					An01g10350	46429
3.2.1.23	*β*-galactosidase	GH35					An14g05820	41910
3.2.1.23	*β*-galactosidase	GH35	[26]		**lacA**	P29853	An01g12150	51764
3.1.1.6	Acetyl esterase	CE16					An02g02540	N/A

For each isoenzyme, putative or characterized, is noted literature references and the gene ID in the sequencings of *A. niger* CBS 513.88 and ATCC 1015. CAZy families are shown in the CAZy columnn [37], The list of necessary enzymes are gathered from the work described in refs. [18-21].

For a number of the studies found in the literature search, it was not possible to link the characterized enzymes with a sequence as only molecular mass and/or isoelectric point of the characterized enzyme(s) were given. However, as these characterizations still include valuable information on the enzymatic capabilities of *A. niger*, in Additional file 17: Table S17 includes an overview of the literature on characterizations of 24 enzymatic activities, where the gene could not be determined.

The mapping of these 16 structures includes information on 115 unique putative and characterized carbohydrate-active genes from the *A. niger* CBS 513.88 sequence [3], and 106 from the *A. niger* ATCC 1015 sequence (117 unique genes) [4]. Of these, the products of 57 of the genes have been previously characterized (See Additional file 17 for references). In total, the integrated information includes references to 203 articles. A full overview of the structures and the integrated genes is available in Additional file 18.

This map also includes a section with an overview of all of the 171 genes identified to code for putative or known polysaccharide-acting enzymes sorted into gene families. A plotting of expression indices directly on the structures of the map as well as in the overview section makes a systems-wide examination possible as described in the following.

### Transcriptome analysis

To assess the regulatory network of genes for extracellular enzymes, *A. niger* was cultivated on six different carbon sources: three mono-saccharides (D-glucose, L-arabinose, and D-xylose), and three complex polysaccharides with a defined composition of sugars (starch, arabinan, and oat spelt xylan). Crude preparations of polysaccharides may include a multitude of undefined sugars and other types of compounds. The use of defined complex polysaccharides in this type of analysis allows for stronger conclusions on which saccharides induce which genes.

The batch cultivations were performed in shake flasks and samples were taken for transcriptome analysis and determination of free sugar concentrations (Table 2). Pairwise statistical comparisons of data from the sets of biological replicates were performed, and the number of statistically significantly (Benjamini-Hochberg adjusted p-values < 0.05) regulated genes in each comparison is shown in Table 3.

**Table 2:** Biomass and sugar concentrations at the time of RNA-sampling from cultivations on six different carbon sources


	**Biomass**	**Xylose**	**Arabinose**	**Glucose**
**Carbon source**	**[g/L]**	**[mM]**	**[mM]**	**[mM]**
Arabinan	4.28±0.42	0.00±0.00	5.79±2.33	-
Arabinose	5.72±0.30	0.00±0.00	45.03±6.79	0.00±0.00
Glucose	6.49±0.96	-	-	34.75±4.66
Starch	7.49±0.60	-	-	23.48±0.56
Xylan	9.00±0.36	4.40±0.60	0.80±0.73	-
Xylose	6.24±0.21	42.56±19.45	-	-

All values are shown as average±standard deviation. Cells marked with - were not measured.

**Table 3:** Overview of significantly regulated genes between cultivations on six different carbon sources


	**Xylan**	**Starch**	**Arabinan**	**Xylose**	**Glucose**
**Arabinose**	991 (318*↑*/673*↓*)	220 (110*↑*/110*↓*)	1281(1048*↑*/233*↓*)	25 (6*↑*/19*↓*)	92 (16*↑*/76*↓*)
**Glucose**	1087 (375*↑*/712*↓*)	27 (17*↑*/10*↓*)	1874 (1485*↑*/389*↓*)	59 (50*↑*/9*↓*)	
**Xylose**	387 (109*↑*/278*↓*)	124 (52*↑*/72*↓*)	1844 (1449*↑*/395*↓*)		
**Arabinan**	2999 (509*↑*/2492*↓*)	1936 (473*↑*/1463*↓*)			
**Starch**	361 (127*↑*/234*↓*)				

The first number in the cells is the number of significantly regulated genes in the pairwise comparison of the carbon sources in the top row and left column. The number marked with an *↑*are the genes that are up-regulated on the carbon source in the top row relative to the one in the left column, while the number with an *↓*denotes the opposite.

In an examination of the sugar-concentration measurements of Table 2, it is rather clear that the sugar-profiles at the time of sampling for transcriptome analysis are quite different, thus giving clear difference between the experimental conditions. The only exception is the profiles on the D-glucose and starch media, where the concentrations of free D-glucose are rather similar. This is most likely due to the fact that the strain employed is an amylase-producer, meaning that starch is rapidly be hydrolyzed to D-glucose, thus making the actual difference between the two carbon sources small. This is reflected in the transcriptome comparison of Table 3, where it is seen that only 27 genes show significant changes in expression between D-glucose and starch. These genes could either be false positives, genes responding to polysaccharides present in the degraded starch medium, or genes responding to the difference in D-glucose concentration between the two conditions (Table 3). While polysaccharide induction is not unlikely, *A. niger* is also known to have gradual responses from 0.5 – 100 mM D-glucose [38].

In a closer examination of Table 3, it is seen that a larger number of genes are responding to the polysaccharides xylan and arabinan compared to the monosaccharides and starch. This is expected since degradation of the diverse components and types of covalent bonds constituting complex carbon sources must require a larger set of genes than a simple one-sugar monosaccharide substrate. Furthermore, as can be seen in Table 2, the concentration of free sugars is roughly an order of magnitude lower in the medium containing the complex carbon sources. It has been shown that CreA mediates carbon repression of xylanolytic enzymes by D-xylose, beginning at 1 mM and increases in strength up to in the area between 30–70 mM [39,40]. These ranges are similar to the concentrations in the comparison of D-xylose and xylan in this study. Similar effects within the same ranges of concentration are known for AraR and XlnR (L-Arabinose and D-xylose metabolism [41-44]), as well as for AmyR (Glucose-repression [38]). Cross-regulation of these carbon-repressing proteins has also been reported [40]. The free sugars are seemingly released from the polysaccharides (except starch) in a rate that effectively lessens the effects of carbon repression on these media.

The differences in induction were examined by preparing a map of the carbohydrate active enzymes for each pairwise comparison. For each map, the statistically significantly changed gene expressions are shown (Additional files 1, 2, 3, 4, 5, 6, 7, 8, 9, 10, 11, 12, 13, 14, 15, 16, 19, 20, 21, 22, 23 and 24). This gives maps of the enzymatic activities induced by a specific monosaccharide or the required enzymatic activities for degradation of a specific polysaccharide. A few examples of comparisons that showed results on a systemic level are discussed here:

**Figure 2:**
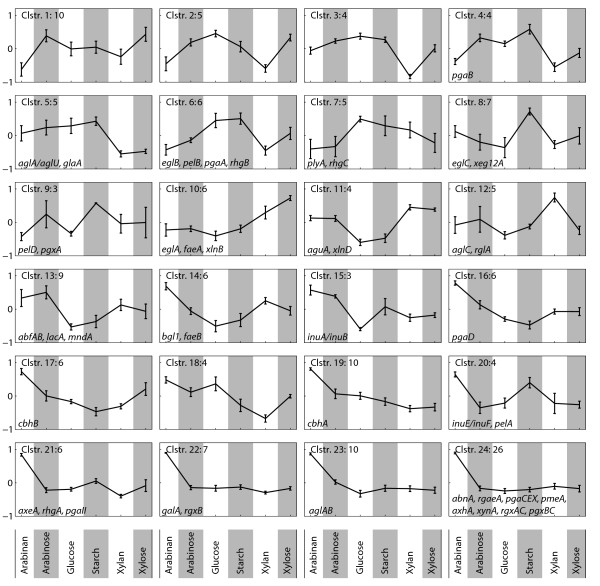
**Clustering of 161 putative and characterized genes coding for polysaccharide-active enzymes according to expression profiles.** The number of genes in each cluster is shown next to the cluster number. The gene names of known genes found in a particular cluster are found in each cluster. The genes were clustered using the ClusterLustre algorithm [50].

#### Arabinan versus L-arabinose

The comparison of these two carbon sources (Additional file 19) showed a diverse response. Regulation was found in 27 of the 36 carbohydrate-acting gene families predicted to be present in *A. niger*[3]. As the mapping shows, the induction on arabinan unsurprisingly includes all of the activities required to degrade arabinan (Additional file 17: Table S15), but also the entire sets of genes required to degrade the pectin components xylogalacturonan, arabinogalactan and rhamnogalacturonan. This is very likely due to the presence of small amounts of rhamnose, galactose, and galacturonic acid in the preparation of arabinan, as described in the materials section. Galacturonic acid is known to induce pectinolytic enzymes [12,14]. The induction on arabinan also includes activities directed towards acid-residues not described in the arabinan preparation, e.g. genes putatively acting on glucuronic acid of arabinogalactan, rhamnogalacturonan acetyl esterase (*rgaeA*), and ferulic acid esterase B (*faeB*) acting on arabinan. These enzymes are reacting to compounds found in arabinan and rhamnogalacturonan I (where arabinan is a component, see Additional files 15 and 16). It is therefore likely that triggering residues are present in minute amounts in the preparation.

#### Arabinose versus D-glucose

Despite only 92 genes being regulated, a mapping of these genes revealed that 13 of them were coding for putative polysaccharide-active gene products (Additional file 21) and all of them induced by L-arabinose. Interestingly, all three of the induced arabinan-acting genes were exo-acting arabinofuranosidases (EC 3.2.1.55), including the characterized *abfA* and *abfB*[5,10,11,39,45-49]. However, the remaining induced genes constitute all of the activities of the xylan-acting genes (Additional file 17: Table S9), including *β*-galactosidase *lacA*, endo-xylanases, and *α*-galacturonase A (*aguA*), suggesting that L-arabinose alone can induce the entire complex of xylan-degrading enzymes.

#### Starch versus xylan

A comparison of these two carbon sources is especially interesting, as no bond-types are shared between the two. Mapping of the significantly regulated genes (Additional file 22) indicates just this. The regulated genes are limited to all of the activities required to degrade starch (up-regulated on starch) and the activities required for degradation of xylan (up-regulated on xylan). Those up-regulated on starch include glucoamylase A (*glaA*), *α*-glucosidase A (*aglA/aglU*) as well as one or more of the three genes for *α*-amylase (*amyA*, *amyB*, and gene ID 140567) with almost identical nucleotide-level sequences. It is not possible with an expression analysis to differentiate between them. The genes induced on xylan are the same genes as described to be induced by L-arabinose in the comparison of L-arabinose and D-glucose, with the exception that the endo-xylanase *xlnB* is induced on xylan instead of the putative endo-xylanase An15g04550/183088 that is induced by L-arabinose. We have thus identified a full set of extracellular polysaccharide-acting genes for the degradation of oat spelt xylan and starch and shown that with the exception of *xlnB*, L-arabinose induces the same array of xylan-degrading enzymes as does xylan (at the concentrations examined here).

### Transcription-based clustering

To further investigate the regulation of single genes and the functions of genes that appear to be co-regulated, a clustering analysis of the expression indices of all 161 putative and characterized glucoside hydrolases, polysaccharide lyases and carbohydrate esterases on the six different carbon sources was performed, thus including also genes which could not be assigned directly to a polysaccharide structure. (Figure 2). 24 gene clusters were identified. Clustering allows the identification of genes that are only induced by the complex substrates, as well as genes that are induced equally well by a particular monosaccharide as well as the complex polysaccharide.

The transcription profile of each cluster (Figure 2) was examined and classified in terms of regulation on the different carbon sources (Additional file 17: Table S18). If a distinct regulation was evident from the clustering profile, this information was added to the table as well, making this an overview of regulation and possible polysaccharide specificity for each of the 161 genes in the survey. The same study was made for the subset of genes coding for carbohydrate-active enzymes that were significantly regulated in one or more pairwise comparisons (Additional file 23 and Additional file 17: Table S19). This subset consists of 103 of the 161 genes, and 47 of the 57 characterized genes. However, as can be seen in Additional file 23, the clustering of the subset is very similar to those of the full set of genes (Figure 2). A comparison of the clusters of the two studies are also present in Additional file 17: Table S19. Since the majority of the genes were significantly regulated, and the remainder fall mostly in the same clusters, the following detailed analysis of the clustering was made using the full set, thereby giving information on the expression patterns of as many genes as possible.

As it is evident from Figure 2, clusters 21–24 are highly similar. In making the clusters, it was attempted to use fewer clusters, and thereby combine these four clusters, however this combination required a decrease of the total numbers of clusters to 10, which increased the variation in the other clusters dramatically (Data not shown). For this reason, it was concluded that they have distinct patterns, as the small standard deviations of the clusters also suggest, and they have been kept as separate clusters. Furthermore, as clusters 21–24 are almost solely induced on arabinan, one would need to include more pectin-like substrates, e.g. polygalacturonic acid, in the analysis to be able to differentiate between the regulation of these genes. However, what one can conclude from this is that the genes of cluster 24 are specific for arabinan, and are not induced by any of the other saccharides in this study. It is interesting that *axhA* clusters with this group, as it was previously shown to be induced on birchwood xylan but not on D-xylose [41]. However, no induction on xylan is seen for cluster 24, suggesting that this gene is not induced by oat spelt xylan in this strain.

In examining the clusters for general trends, it was found that for most of the clusters, regulation on D-glucose and starch are very similar. This is in good agreement with the high level of free D-glucose in both cultures shown in Table 2. Exceptions are clusters 8, 9, 15, 18, and 20, which should be interesting for determining genes that are sensitive to degradation-products of starch, but not necessarily statistically significant in the comparison of starch versus D-glucose (Table 3).

For each cluster, a map marking the genes of that cluster was prepared and examined to determine an activity profile if possible (Additional file 24).

Several of the clusters are targeted to a specific type of polysaccharide. One example hereof is cluster 1. While it does not containing any characterized genes, the genes of the cluster are quite specifically predicted to code for enzymes with activity towards galactomannan and insoluble galactoglucomannan (Additional file 24). This homogeneity of functions confirms the putative annotations. As the cluster is up-regulated on the mono-sugars and the relatively easily degradable potato starch, it is likely that this cluster contains genes are induced by mono-sugar-abundance and code for a set of “scouting enzymes” with the role to liberate more substrates.

**Figure 3:**
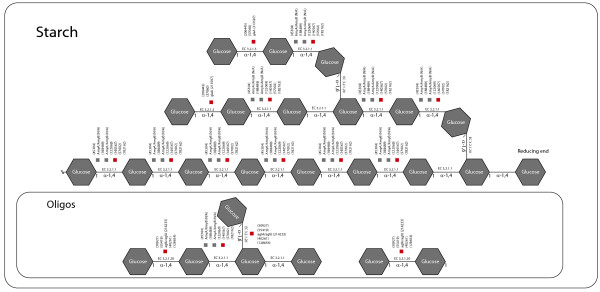
**Map of starch-degrading enzymes in cluster 5.** Genes marked with a red box are found in the cluster, gray boxes means that the gene is found in the *A. niger* CBS 513.88 sequence, but no bi-directional best hit is found in the *A. niger* ATCC 1015 sequence.

Cluster 5 has the highest level of expression on D-glucose and starch carbon sources. An examination of it with the enzyme mapping (Additional file 24), reveals that it constitutes a full set of co-regulated starch-degrading enzymes. The individual genes found in the cluster encode solely amylases and glucosidases, including glucoamylase A (*glaA*) and one or more of the three *α*-amylases with similar sequences discussed above (including *amyA* and *amyB*). The starch part of the map can be found in Figure 3. The fact that these enzymes cluster together can be seen as a validation of the transcription analysis and the clustering employed.

Rather interesting is the observation that the hemicellulose component arabinan seems to induce enzymes acting on cellulose and other *β*-glucans (Additional file 24). This is seen in clusters 17 and 19, each containing one of the characterized cellobiohydrolases (which were shown to be induced on D-xylose [51]) and several putative *β*-glucosidases. *β*-glucans are not found as components of pectins, which makes this pattern a genuine induction across carbon-sources of entirely different chemical make-up.

As discussed above, the profile of cluster 24 indicates that it mainly contains genes that can be described as pectinolytic. A map inspection (Additional file 24) confirms that these genes have a very distinct profile including all enzymatic activities for the degradation of rhamnogalacturonan I, smooth pectin, and xylogalacturonan, all components of pectin, as are arabinan.

### Comparison of transcriptional regulation to previous studies

As described in the introduction, relatively few large-scale studies exist where the carbon source-based induction of extracellular enzymes have been studied. There is little overlap in the carbon sources employed in this study. Studies of polysaccharides have mainly been done with di-saccharides such as maltose or sucrose [7] or on commercial preparations of pectin for food gelling [5,6]. However, a few have been made, which will be compared to the results of the present study here. A relatively larger number of studies including single or a few genes are published, however, these have been cited in the text above where appropriate, and in Additional file 17.

The method applied here is comparable to a study of one of the authors, where a principal component analysis (PCA) allows clustering according to expression profiles [5]. While that study focuses mainly on pectinolysis, it includes studies of growth on D-glucose, D-xylose, and L-arabinose. The study describes *abfAabfB*, and *lacA* as being co-regulated and induced by 25 mM L-arabinose, which is in good accordance with the results of this work, as all of these are found in cluster 13. Interestingly however, that work also reports *abnA* as being induced by L-arabinose and co-regulated with *abfAB* and *lacA*, but in the present work, only induction on arabinan is seen (cluster 24), and with a statistically significant up-regulation on arabinan in all pairwise comparisons. This may be due to our higher concentration of L-arabinose (45 mM relative to 25 mM) leading to differences in carbon-repression. The PCA also indicates a close relationship between *pmeApgaX*, and *abnA*, which describes accurately the patterns of cluster 24.

*faeA* and *faeB* are known to be largely co-regulated, but *faeA* is induced on D-xylose due to an induction by the xylanolytic regulator XlnR [5,41]. *faeA* and *faeB* are separated in clusters 10 and 14, that are very similar, but cluster 10 responds to D-xylose, whereas cluster 14 responds to the ferulic acid-containing arabinan, suggesting that *faeB* is more targeted to arabinan.

Another study [9] examines induction of genes on D-xylose and maltose, which has an *α*-1,4-glucoside bond similar to starch. In Figure two in ref. [9], eleven genes can be seen to be induced by maltose and/or D-xylose (An11g03340, An04g06930, An01g06120, An14g04190, An12g02450, An09g03070, *agdA*, An01g10930, *glaA*, An09g05880, An09g03300). All of these are regulated in a similar fashion in the present work, if maltose (Glc-*α*-1,4-Glc) is seen to be similar to starch ((Glc-*α*-1,4-Glc-*α*-1,4-)_*n*_).

## Conclusions

Enzyme preparations have multiple areas of application and are a billion dollar market with a low cost/profit ratio. Thus, it is of great importance and interest to gain an understanding of the processes leading to production of enzymes with a specific profile.

In this study, the first genome-scale characterization of all genes coding for carbohydrate-active enzymes identified in *A. niger* is provided. Based on a review of more than 200 articles and sequence database searches, information of 117 genes and enzymes has been systematized according to the polysaccharide structures they degrade, thereby providing an updated reference on extracellular enzyme-expression in *A. niger*. This data-integration effort has produced schematic representations (maps) of 16 types of polysaccharide-structures specifically updated with the *A. niger* enzymatic machinery.

An application of this knowledge was performed in a transcription study where statistical analysis as well as expression-level-based clustering and mapping were employed to provide transcription profiles of 161 genes on six defined carbon sources, thereby adding considerably to our knowledge of the transcription-level regulation of these genes. New knowledge was generated on the carbon source-based transcriptional regulation of previously characterized genes including, but not limited to, *abfA, abfB, aglA, cbhA, cbhB, glaA, eglA, eglB, faeA, faeB, lacA, pelA, pelB, pelC, xlnB*, and *xynA* as well as more than 100 uncharacterized genes predicted to code for carbohydrate-active enzymes. The mapping of transcribed genes allowed us to identify induction by mono-saccharides of complete sets of enzymes for degradation of complex substrates, as well as the induction of cellulolytic enzymes on a hemicellulose substrate, e.g. xylan.

## Methods

### Cultivation procedure

#### Strain information

The strain used was *A. niger* BO-1, a progenitor to high-yield amylase-producing industrial strains, obtained from Novozymes A/S. The strain is maintained as frozen spore suspensions at -80°C in 20 % glycerol.

#### Growth media

Complex medium: 2 g/L yeast extract, 3 g/L tryptone, 10 g/L glucose monohydrate, 20 g/L agar, 0.52 g/L KCl, 0.52 g/L MgSO_4_·7H_2_O, 1.52 g/L KH_2_PO_4_, and 1 mL/L of trace elements solution. Trace element solution: 0.4 g/L CuSO_4_·5H_2_O, 0.04 g/L Na_2_B_4_O_7_·10H_2_O, 0.8 g/L FeSO_4_·7H_2_O, 0.8 g/L MnSO_4_·H_2_O, 0.8 g/L Na_2_MoO_4_·2H_2_O, 8 g/L ZnSO_4_·7H_2_O.

Batch cultivation medium salt concentrations: 2.5 g/L (NH_4_)_2_SO_4_, 0.75 g/L KH_2_PO_4_, 1.0 g/L MgSO_4_·7H_2_O, 1 g/L NaCl, 0.1 g/L CaCl_2_·2H_2_O, 0.05 mL/L antifoam 204 (Sigma), and 1 mL/L trace element solution. Trace element solution composition: 7.2 g/L ZnSO_4_·7H_2_O, 0.3 g/L NiCl_2_·6H_2_O, 6.9 g/L FeSO_4_·7H_2_O, 3.5 g/L MnCl_2_·4H_2_O, and 1.3 g/L CuSO_4_·5H_2_O. The carbon sources used for the cultivations were D-glucose H_2_O (20 g/L), D-xylose (20 g/L), L-arabinose (19.5 g/L + 0.5 g/L D-glucose), sugar beet arabinan (Megazyme, P-ARAB) (16.3 g/L), oat spelt xylan (Sigma, no 95590) (16.2 g/L), and potato starch (Sigma, no 85650) (16.5 g/L). These concentrations all equal 0.61 Cmole/L. Cultivations with L-arabinose were added D-glucose in order to make the spores germinate.

Sugar beet arabinan (Megazyme) is 95% pure 1,5-*α*-L-arabinan containing arabinose:galactose:rhamnose:galacturonic acid in the ratios 88:4:2:6. The documentation further describes the arabinan as being “a polymer of 1,5-*alpha*-L-linked arabinofuranose units which is highly substituted by 1,3- and 1,2-linked single *α*-L-arabinofuranose residues. About 50% of 1,5-linked arabinosyl residues in the main chain are substituted by 1,3 or 1,2 linked arabinofuranosyl branches.”

Oat spelt xylan (Sigma) contains ≤10% L-arabinose, ≤15% D-glucose, and ≥70% D-xylose.

#### Shake flask cultivations

Shake flasks were initiated by spore inoculation to a final concentration of 2×10^9^ spores/L. Spores were propagated on complex medium plates and incubated for 7–8 days at 30°C before being harvested with 10 mL of 0.01% Tween 80. All cultivations were carried out in 500 mL baffled shake flasks with a total volume of 200 mL liquid medium at 30°C and 150 rpm. The initial pH was set to pH 2.5 to avoid pellet formation. The entire content of the shake flask was harvested when the biomass concentration was approximately half the maximum biomass concentration on the given medium (mid-growth phase). All cultivations were performed in biological triplicates except starch, where a duplicate culture was performed.

#### Sampling

Cell dry weight was determined using nitrocellulose filters (pore size 0.45 *μ*m, Gelman Sciences). The filters were pre-dried in a microwave oven at 150 W for 15 minutes, cooled in a desiccator and subsequently weighed. A known volume of cell culture was filtered and the residue was washed with 0.9% NaCl and dried on the filter for 15 minutes in a microwave oven at 150 W and cooled in a desiccator. The filtrate was saved for quantification of sugars and extracellular metabolites and stored at -80°C. The filter was weighed again and the cell mass concentration was calculated. These values were used to calculate maximum specific growth rates. For gene expression analysis, mycelium was harvested at the mid-late exponential phase (at approximately half the maximum concentration of biomass) by filtration through sterile Mira-Cloth (Calbiochem) and washed with a PBS buffer (8 g/L NaCl, 0.20 g/L KCl, 1.44 g/L Na_2_HPO_4_, and 0.24 g/L KH_2_PO_4_ in distilled water). The mycelium was quickly dried by squeezing, and subsequently frozen in liquid nitrogen. Samples were stored at -80°C until RNA extraction.

#### Quantification of sugars

The concentrations of sugar in the filtrates were determined using HPLC on an Aminex HPX-87H ion-exclusion column (BioRad, Hercules, CA). The column was eluted at 60°C with 5 mM H_2_SO_4_ at a flow rate of 0.6 mL/min. Sugars were detected with a refractive index detector and a UV detector.

### Transcriptome analysis

#### Extraction of total RNA

40–50 mg of frozen mycelium was placed in a 2 mL Eppendorf tube, pre-cooled in liquid nitrogen, containing three steel balls (two balls with a diameter of 2 mm and one ball with a diameter of 5 mm). The tubes were then shaken in a Mixer Mill, at 5°C for 10 minutes, until the mycelium was ground to powder. Total RNA was isolated from the powder using the Qiagen RNeasy Mini Kit, according to the protocol for isolation of total RNA from plant and fungi. The quality of the extracted total RNA was assessed using a BioAnalyzer 2100 (Agilent Technologies Inc., Santa Clara, CA, USA) and the quantity determined using a spectrophotometer (GE Healthcare Bio-Sciences AB, Uppsala, Sweden). The total RNA was stored at -80°C until further processing.

#### Preparation of biotin-labeled cRNA and microarray processing

15 *μ*g of fragmented biotin-labeled cRNA was prepared from 5 *μ*g of total RNA and hybridized to the 3AspergDTU GeneChip [13] according to the Affymetrix GeneChip Expression Analysis Technical Manual [52].

cRNA was quantified in a spectrophotometer (same as above). cRNA quality was assessed using a BioAnalyzer. A GeneChip Fluidics Station FS-400 (fluidics protocol FS450_001) and a GeneChip Scanner 3000 were used for hybridization and scanning.

The scanned probe array images (.DAT files) were converted into .CEL files using the GeneChip Operating Software (Affymetrix).

#### Analysis of transcription data

Affymetrix CEL-data files were preprocessed using the statistical language and environment R [53] version 2.6.1. The probe intensities were normalized for background using the RMA method [54] using only perfect match (PM) probes. Normalization was performed subsequently using the quantiles algorithm [55]. Gene expression values were calculated from the PM probes with the medianpolish summary method [54]. All statistical preprocessing methods were used by invoking them through the affy package [56].

Statistical analysis was applied to determine genes subject to differential transcriptional regulation. The limma package [57] was used to perform moderated t-tests between two sets of triplicates from each pH level. Empirical Bayesian statistics were used to moderate the standard errors within each gene and Benjamini-Hochberg’s method [58] to adjust for multi-testing. A cut-off value of adjusted p<0.05 was set to assess statistical significance.

Normalized and raw data-values are deposited with GEO as series GSE11930.

### Clustering

Genes were clustered using the clustering algorithm ClustreLustre [50], using *k*-means clustering, a pearson-based distance measure and accounting for biological replicates.

## Competing interests

The authors declare that they have no competing interests

## Authors’ contributions

MRA conceived the study, designed the experiments, analyzed the data and wrote the manuscript. MGJ conducted the experiments, designed the experiments and analyzed data. RPdV analyzed data and wrote the manuscript. JN conceived the study, designed the experiments, and wrote the manuscript. All authors read and approved the final manuscript.

## Supplementary Material

Additional file 1: **Schematic representation of starch.** The nature of the bonds between the sugar units are noted where they are known. The number of sides on the sugar polygons reflect the number of carbons of the sugar. The first carbon is indicated on all sugars with a 1 to clarify the bond configurations. Carbons are numbered clockwise from the first carbon with the two last carbons of the sugar (hexose or pentose) on the same corner of the polygon. The oligos are hypothetical hydrolysis products and sugars that appear due to the action of exo-acting enzymes. The structure is based on ref. [59]. Click here for file

Additional file 2: **Schematic representation of cellulose.** The nature of the bonds between the sugar units are noted where they are known. The number of sides on the sugar polygons reflect the number of carbons of the sugar. The first carbon is indicated on all sugars with a 1 to clarify the bond configurations. Carbons are numbered clockwise from the first carbon with the two last carbons of the sugar (hexose or pentose) on the same corner of the polygon. The oligos are hypothetical hydrolysis products and sugars that appear due to the action of exo-acting enzymes. The structure is based on refs. [20,60]. Click here for file

Additional file 3: **Schematic representation of pullulan.** The nature of the bonds between the sugar units are noted where they are known. The number of sides on the sugar polygons reflect the number of carbons of the sugar. The first carbon is indicated on all sugars with a 1 to clarify the bond configurations. Carbons are numbered clockwise from the first carbon with the two last carbons of the sugar (hexose or pentose) on the same corner of the polygon. The oligos are hypothetical hydrolysis products and sugars that appear due to the action of exo-acting enzymes. The structure is based on refs. [59,61]. Click here for file

Additional file 4: **Schematic representation of inulin.** The nature of the bonds between the sugar units are noted where they are known. The number of sides on the sugar polygons reflect the number of carbons of the sugar. The first carbon is indicated on all sugars with a 1 to clarify the bond configurations. Carbons are numbered clockwise from the first carbon with the two last carbons of the sugar (hexose or pentose) on the same corner of the polygon. The oligos are hypothetical hydrolysis products and sugars that appear due to the action of exo-acting enzymes. The structure is based on ref. [62]. Click here for file

Additional file 5: **Schematic representation of galactomannan.** The nature of the bonds between the sugar units are noted where they are known. The number of sides on the sugar polygons reflect the number of carbons of the sugar. The first carbon is indicated on all sugars with a 1 to clarify the bond configurations. Carbons are numbered clockwise from the first carbon with the two last carbons of the sugar (hexose or pentose) on the same corner of the polygon. The oligos are hypothetical hydrolysis products and sugars that appear due to the action of exo-acting enzymes. The structure is based on refs. [20,63]. Click here for file

Additional file 6: **Schematic representation of insoluble galactoglucomannan.** The nature of the bonds between the sugar units are noted where they are known. The number of sides on the sugar polygons reflect the number of carbons of the sugar. The first carbon is indicated on all sugars with a 1 to clarify the bond configurations. Carbons are numbered clockwise from the first carbon with the two last carbons of the sugar (hexose or pentose) on the same corner of the polygon. The oligos are hypothetical hydrolysis products and sugars that appear due to the action of exo-acting enzymes. The structure is based on ref. [20]. Click here for file

Additional file 7: **Schematic representation of soluble galactoglucomannan.** The nature of the bonds between the sugar units are noted where they are known. The number of sides on the sugar polygons reflect the number of carbons of the sugar. The first carbon is indicated on all sugars with a 1 to clarify the bond configurations. Carbons are numbered clockwise from the first carbon with the two last carbons of the sugar (hexose or pentose) on the same corner of the polygon. The oligos are hypothetical hydrolysis products and sugars that appear due to the action of exo-acting enzymes. The structure is based on ref. [18-21]. Click here for file

Additional file 8: **Schematic representation of smooth pectin.** The nature of the bonds between the sugar units are noted where they are known. The number of sides on the sugar polygons reflect the number of carbons of the sugar. The first carbon is indicated on all sugars with a 1 to clarify the bond configurations. Carbons are numbered clockwise from the first carbon with the two last carbons of the sugar (hexose or pentose) on the same corner of the polygon. The oligos are hypothetical hydrolysis products and sugars that appear due to the action of exo-acting enzymes. The structure is based on refs. [20,60]. Click here for file

Additional file 9: **Schematic representation of xylogalactouronan.** The nature of the bonds between the sugar units are noted where they are known. The number of sides on the sugar polygons reflect the number of carbons of the sugar. The first carbon is indicated on all sugars with a 1 to clarify the bond configurations. Carbons are numbered clockwise from the first carbon with the two last carbons of the sugar (hexose or pentose) on the same corner of the polygon. The oligos are hypothetical hydrolysis products and sugars that appear due to the action of exo-acting enzymes. The structure is based on refs. [20,64-67]. Click here for file

Additional file 10: **Schematic representation of xylan.** The nature of the bonds between the sugar units are noted where they are known. The number of sides on the sugar polygons reflect the number of carbons of the sugar. The first carbon is indicated on all sugars with a 1 to clarify the bond configurations. Carbons are numbered clockwise from the first carbon with the two last carbons of the sugar (hexose or pentose) on the same corner of the polygon. The oligos are hypothetical hydrolysis products and sugars that appear due to the action of exo-acting enzymes. The structure is based on refs. [20,60,68,69]. Click here for file

Additional file 11: **Schematic representation of xyloglucan type XXGG.** The nature of the bonds between the sugar units are noted where they are known. The number of sides on the sugar polygons reflect the number of carbons of the sugar. The first carbon is indicated on all sugars with a 1 to clarify the bond configurations. Carbons are numbered clockwise from the first carbon with the two last carbons of the sugar (hexose or pentose) on the same corner of the polygon. The oligos are hypothetical hydrolysis products and sugars that appear due to the action of exo-acting enzymes. The structure is based on refs. [20,70,71]. Click here for file

Additional file 12: **Schematic representation of xyloglucan type XXXG.** The nature of the bonds between the sugar units are noted where they are known. The number of sides on the sugar polygons reflect the number of carbons of the sugar. The first carbon is indicated on all sugars with a 1 to clarify the bond configurations. Carbons are numbered clockwise from the first carbon with the two last carbons of the sugar (hexose or pentose) on the same corner of the polygon. The oligos are hypothetical hydrolysis products and sugars that appear due to the action of exo-acting enzymes. The structure is based on refs. [20,70,71]. Click here for file

Additional file 13: **Schematic representation of arabinogalactan type I.** The nature of the bonds between the sugar units are noted where they are known. The number of sides on the sugar polygons reflect the number of carbons of the sugar. The first carbon is indicated on all sugars with a 1 to clarify the bond configurations. Carbons are numbered clockwise from the first carbon with the two last carbons of the sugar (hexose or pentose) on the same corner of the polygon. The oligos are hypothetical hydrolysis products and sugars that appear due to the action of exo-acting enzymes. The structure is based on refs. [12,72,73]. Click here for file

Additional file 14: **Schematic representation of arabinogalactan type II (protein bound arabinogalactan).** The nature of the bonds between the sugar units are noted where they are known. The number of sides on the sugar polygons reflect the number of carbons of the sugar. The first carbon is indicated on all sugars with a 1 to clarify the bond configurations. Carbons are numbered clockwise from the first carbon with the two last carbons of the sugar (hexose or pentose) on the same corner of the polygon. The oligos are hypothetical hydrolysis products and sugars that appear due to the action of exo-acting enzymes. The structure is based on [12,72,74,75]. Click here for file

Additional file 15: **Schematic representation of arabinan.** The nature of the bonds between the sugar units are noted where they are known. The number of sides on the sugar polygons reflect the number of carbons of the sugar. The first carbon is indicated on all sugars with a 1 to clarify the bond configurations. Carbons are numbered clockwise from the first carbon with the two last carbons of the sugar (hexose or pentose) on the same corner of the polygon. The oligos are hypothetical hydrolysis products and sugars that appear due to the action of exo-acting enzymes. The structure is based on refs. [76,77,78]. Click here for file

Additional file 16: **Schematic representation of rhamnogalacturonan type I.** The nature of the bonds between the sugar units are noted where they are known. The number of sides on the sugar polygons reflect the number of carbons of the sugar. The first carbon is indicated on all sugars with a 1 to clarify the bond configurations. Carbons are numbered clockwise from the first carbon with the two last carbons of the sugar (hexose or pentose) on the same corner of the polygon. The oligos are hypothetical hydrolysis products and sugars that appear due to the action of exo-acting enzymes. The structure is based on refs. [20,76,77,79,80]. Click here for file

Additional file 17: **Tables of enzymatic activities required for the degradation of 16 different plant-derived polysaccharides.** For each isoenzyme, putative or characterized, is noted literature references and the gene ID in the whole genome sequencings of *A. niger* CBS 513.88 and ATCC 1015 [3-7,10-12,14,18-36,39-41,45-49,51,59-68,70-226]. Click here for file

Additional file 18: **Full map of 16 polysaccharide structures with the addition of genes from * A. niger * ATCC 1015 specific for each type of bond.** The nature of the bonds between the sugar units are noted where they are known. The number of sides on the sugar polygons reflect the number of carbons of the sugar. The first carbon is indicated on all sugars with a 1 to clarify the bond configurations. Carbons are numbered clockwise from the first carbon with the two last carbons of the sugar (hexose or pentose) on the same corner of the polygon. The oligos are hypothetical hydrolysis products and sugars that appear due to the action of exo-acting enzymes. Type and approximate 2D orientation is shown for each of the chemical bonds connecting sugars and/or acids. Next to each bond, one can find verified and hypothetical enzymes in *A. niger* ATCC 1015 capable of catalyzing the hydrolysis of the bond. The bottom panel denotes CAZy-families for each of the genes. See Additional Files 1-16 for details on literature references for structures. Click here for file

Additional file 19: **Map of statistically significantly regulated genes coding for carbohydrate-active enzymes in a pairwise comparison of growth on arabinan versus L-arabinose.** Genes marked with a red box are significantly up-regulated on arabinan relative to L-arabinose, a green box denotes the opposite, gray boxes means that the gene is found in the *A. niger* CBS 513.88 sequence, but no bi-directional best hit is found in the *A. niger* ATCC 1015 sequence. The absence of a box mean that this gene is not statistically significantly regulated in this comparison. Click here for file

Additional file 20: **Map of statistically significantly regulated genes coding for carbohydrate-active enzymes in a pairwise comparison of growth on starch versus D-glucose.** Genes marked with a red box are significantly up-regulated on starch relative to D-glucose, a green box denotes the opposite, gray boxes means that the gene is found in the *A. niger* CBS 513.88 sequence, but no bi-directional best hit is found in the *A. niger* ATCC 1015 sequence. The absence of a box mean that this gene is not statistically significantly regulated in this comparison. Click here for file

Additional file 21: **Map of statistically significantly regulated genes coding for carbohydrate-active enzymes in a pairwise comparison of growth on L-arabinose versus D-glucose.** Genes marked with a red box are significantly up-regulated on L-arabinose relative to D-glucose, a green box denotes the opposite, gray boxes means that the gene is found in the *A. niger* CBS 513.88 sequence, but no bi-directional best hit is found in the *A. niger* ATCC 1015 sequence. The absence of a box mean that this gene is not statistically significantly regulated in this comparison. Click here for file

Additional file 22: **Map of statistically significantly regulated genes coding for carbohydrate-active enzymes in a pairwise comparison of growth on starch versus xylan.** Genes marked with a red box are significantly up-regulated on starch relative to xylan, a green box denotes the opposite, gray boxes means that the gene is found in the *A. niger* CBS 513.88 sequence, but no bi-directional best hit is found in the *A. niger* ATCC 1015 sequence. The absence of a box mean that this gene is not statistically significantly regulated in this comparison. Click here for file

Additional file 23: **Clustering of 103 significantly regulated genes coding for enzymes with putative and characterized polysaccharide-activities.** All genes were found to be significantly regulated in at least one pairwise-comparison of two carbon sources. Cluster numbers in parentheses is the number of the corresponding cluster(s) in Figure 2. The number of genes in each cluster is shown next to the cluster number. The gene names of known genes found in a particular cluster are found at the bottom of the transcription profile graph of each cluster. The genes were clustered using the ClusterLustre algorithm [50]. Click here for file

Additional file 24: **24 maps of enzymatic activities present in the expression-based clusters 1-24 of Figure **2**.** Genes marked with a red box are found in the cluster, gray boxes means that the gene is found in the *A. niger* CBS 513.88 sequence, but no bi-directional best hit is found in the **A. niger** ATCC 1015 sequence. Click here for file
