# Supplementary figures and images for: Mapping the polysaccharide degradation potential of Aspergillus niger
Source: BMC Genomics. 2012 Jul 16;13:313. doi: 10.1186/1471-2164-13-313 (PMC3542576; doi:10.1186/1471-2164-13-313)

# Starch

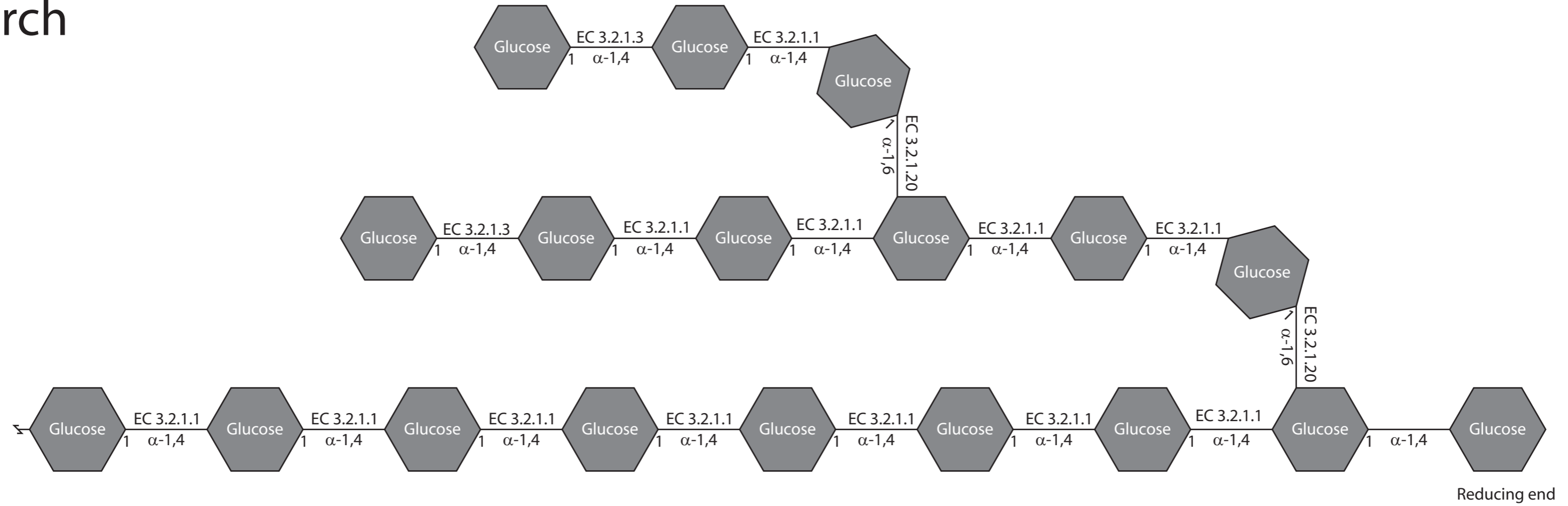

## Oligos

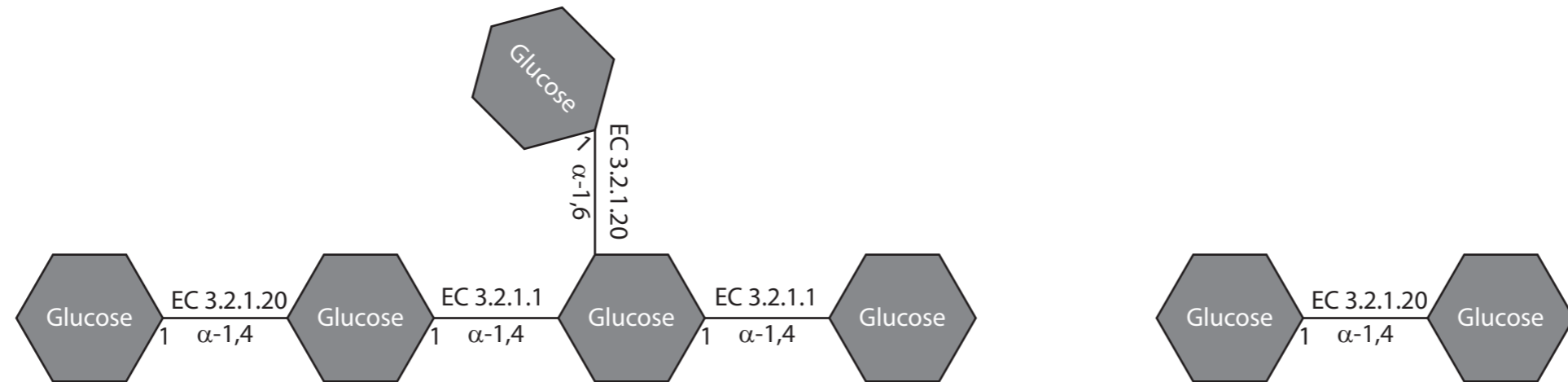

Supplement: Additional file 1: — Schematic representation of starch. The nature of the bonds between the sugar units are noted where they are known. The number of sides on the sugar polygons reflect the number of carbons of the sugar. The first carbon is indicated on all sugars with a 1 to clarify the bond configurations. Carbons are numbered clockwise from the first carbon with the two last carbons of the sugar (hexose or pentose) on the same corner of the polygon. The oligos are hypothetical hydrolysis products and sugars that appear due to the action of exo-acting enzymes. The structure is based on ref. [59]. [file 1471-2164-13-313-S1.pdf]

# Cellulose

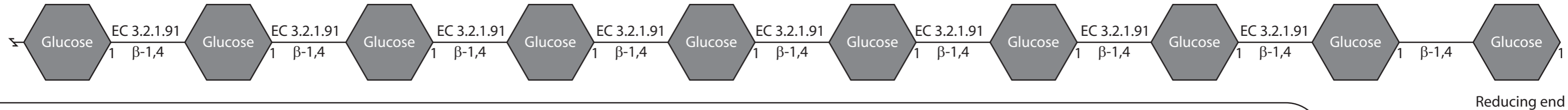

## Cellodextrins

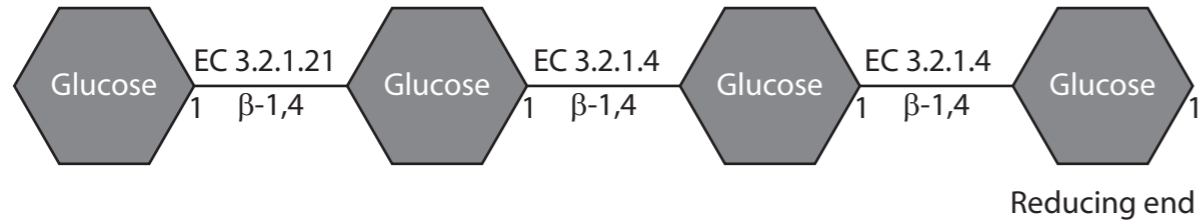

## Cellobiose

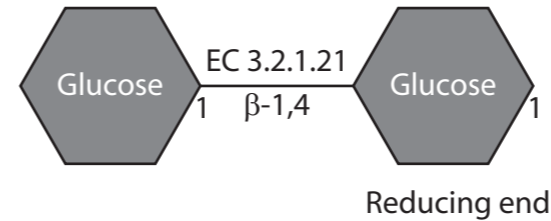

Supplement: Additional file 2: — Schematic representation of cellulose. The nature of the bonds between the sugar units are noted where they are known. The number of sides on the sugar polygons reflect the number of carbons of the sugar. The first carbon is indicated on all sugars with a 1 to clarify the bond configurations. Carbons are numbered clockwise from the first carbon with the two last carbons of the sugar (hexose or pentose) on the same corner of the polygon. The oligos are hypothetical hydrolysis products and sugars that appear due to the action of exo-acting enzymes. The structure is based on refs. [20,60]. [file 1471-2164-13-313-S2.pdf]

# Pullulan

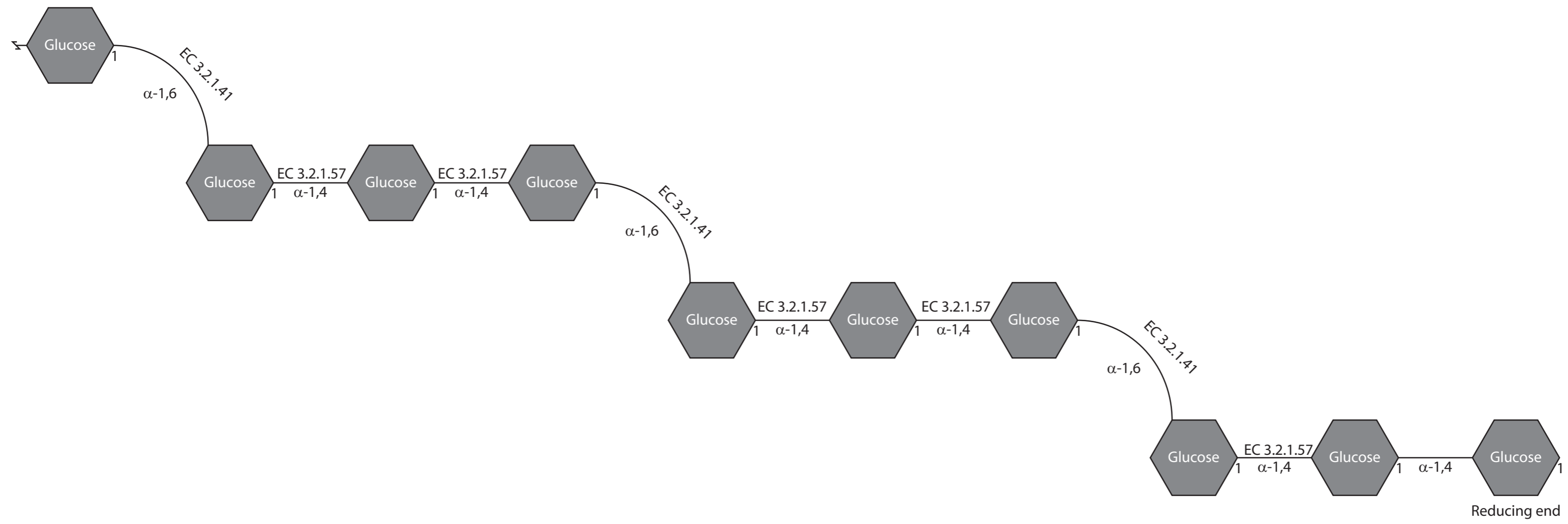

# Oligos

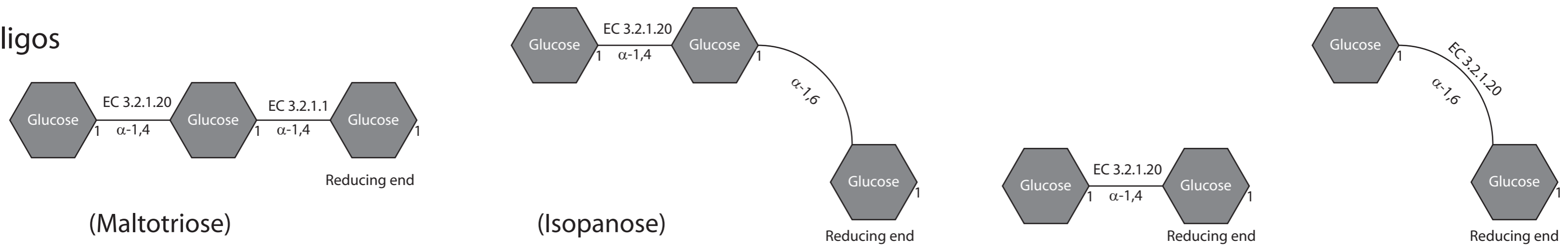

Supplement: Additional file 3: — Schematic representation of pullulan. The nature of the bonds between the sugar units are noted where they are known. The number of sides on the sugar polygons reflect the number of carbons of the sugar. The first carbon is indicated on all sugars with a 1 to clarify the bond configurations. Carbons are numbered clockwise from the first carbon with the two last carbons of the sugar (hexose or pentose) on the same corner of the polygon. The oligos are hypothetical hydrolysis products and sugars that appear due to the action of exo-acting enzymes. The structure is based on refs. [59,61]. [file 1471-2164-13-313-S3.pdf]

# Inulin

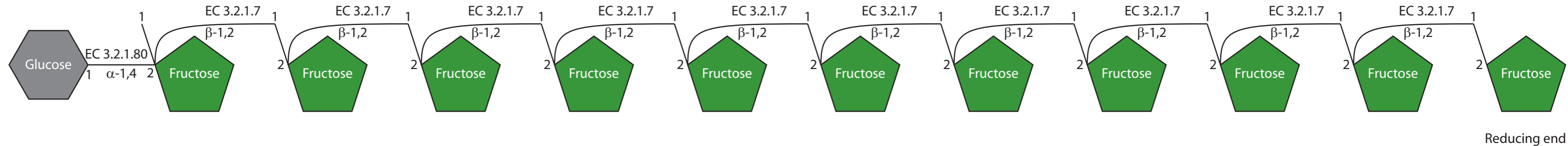

# Oligos

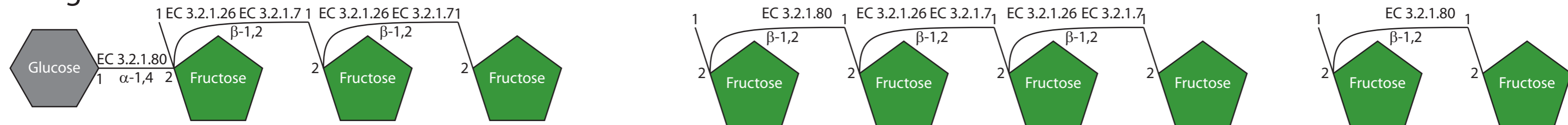

Supplement: Additional file 4: — Schematic representation of inulin. The nature of the bonds between the sugar units are noted where they are known. The number of sides on the sugar polygons reflect the number of carbons of the sugar. The first carbon is indicated on all sugars with a 1 to clarify the bond configurations. Carbons are numbered clockwise from the first carbon with the two last carbons of the sugar (hexose or pentose) on the same corner of the polygon. The oligos are hypothetical hydrolysis products and sugars that appear due to the action of exo-acting enzymes. The structure is based on ref. [62]. [file 1471-2164-13-313-S4.pdf]

# Galactomannan

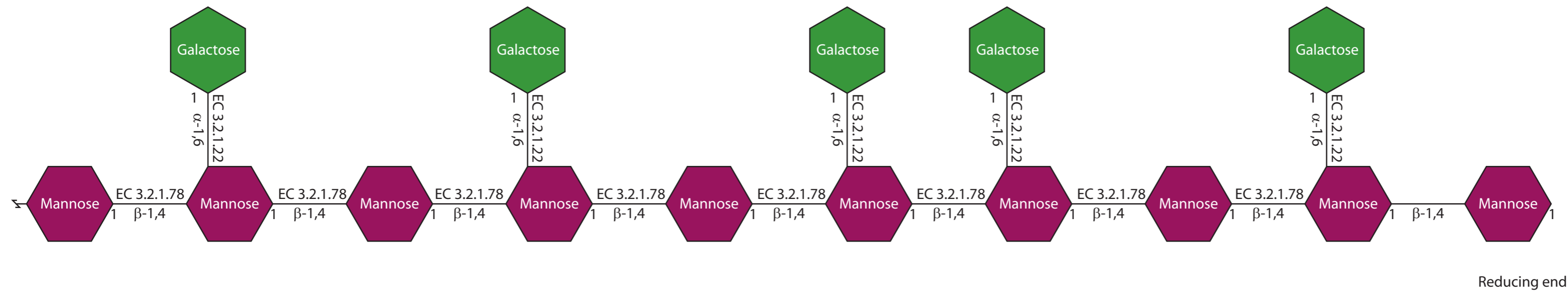

## Oligos

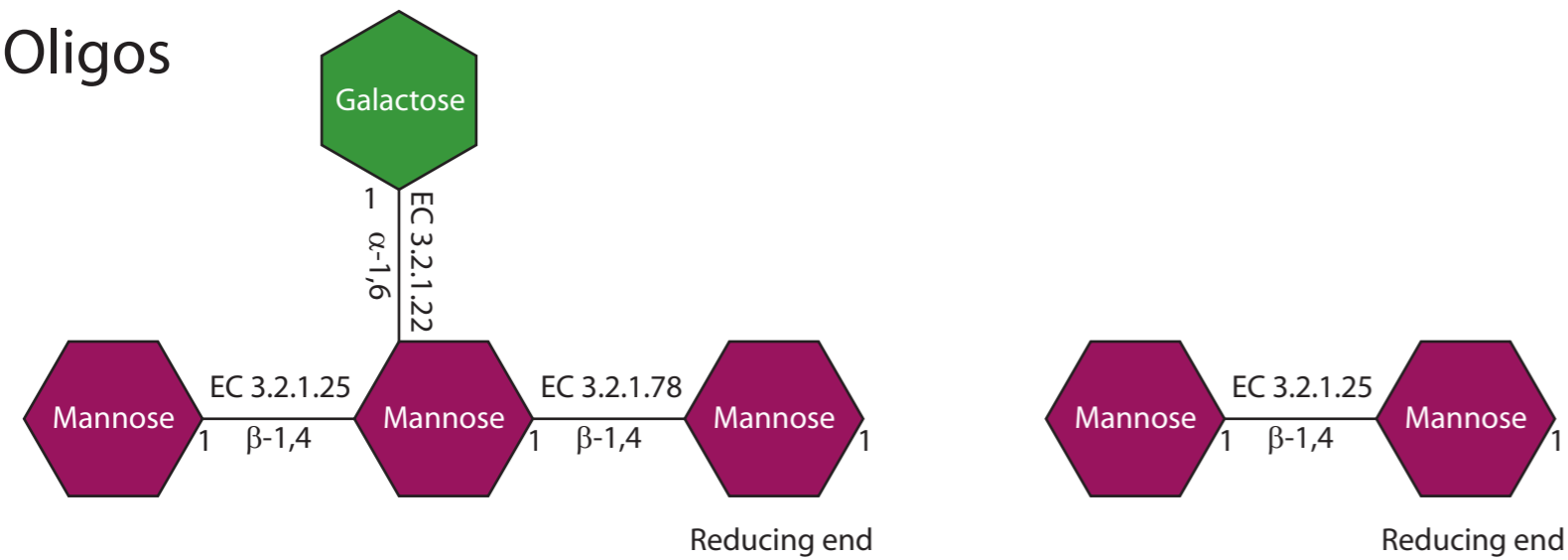

Supplement: Additional file 5: — Schematic representation of galactomannan. The nature of the bonds between the sugar units are noted where they are known. The number of sides on the sugar polygons reflect the number of carbons of the sugar. The first carbon is indicated on all sugars with a 1 to clarify the bond configurations. Carbons are numbered clockwise from the first carbon with the two last carbons of the sugar (hexose or pentose) on the same corner of the polygon. The oligos are hypothetical hydrolysis products and sugars that appear due to the action of exo-acting enzymes. The structure is based on refs. [20,63]. [file 1471-2164-13-313-S5.pdf]

# Galactoglucomannan (insoluble)

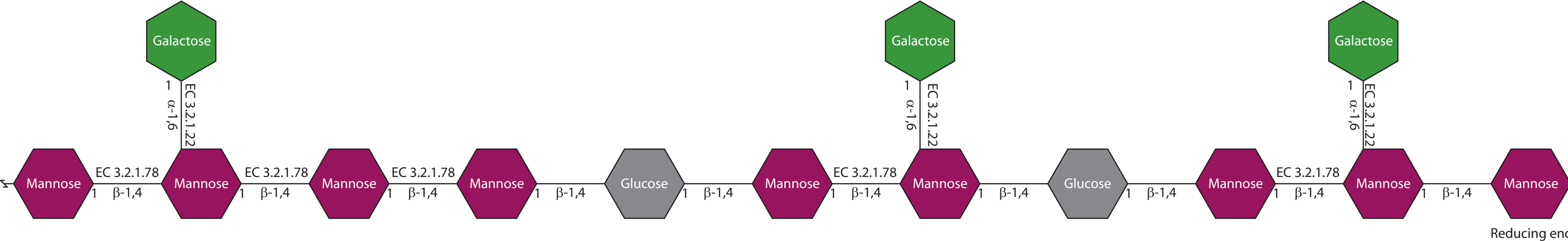

## Oligos

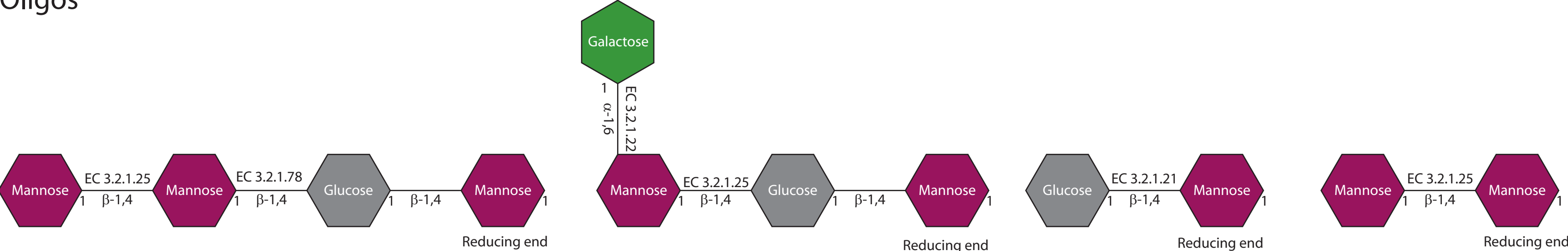

Supplement: Additional file 6: — Schematic representation of insoluble galactoglucomannan. The nature of the bonds between the sugar units are noted where they are known. The number of sides on the sugar polygons reflect the number of carbons of the sugar. The first carbon is indicated on all sugars with a 1 to clarify the bond configurations. Carbons are numbered clockwise from the first carbon with the two last carbons of the sugar (hexose or pentose) on the same corner of the polygon. The oligos are hypothetical hydrolysis products and sugars that appear due to the action of exo-acting enzymes. The structure is based on ref. [20]. [file 1471-2164-13-313-S6.pdf]

# Galactoglucomannan (soluble)

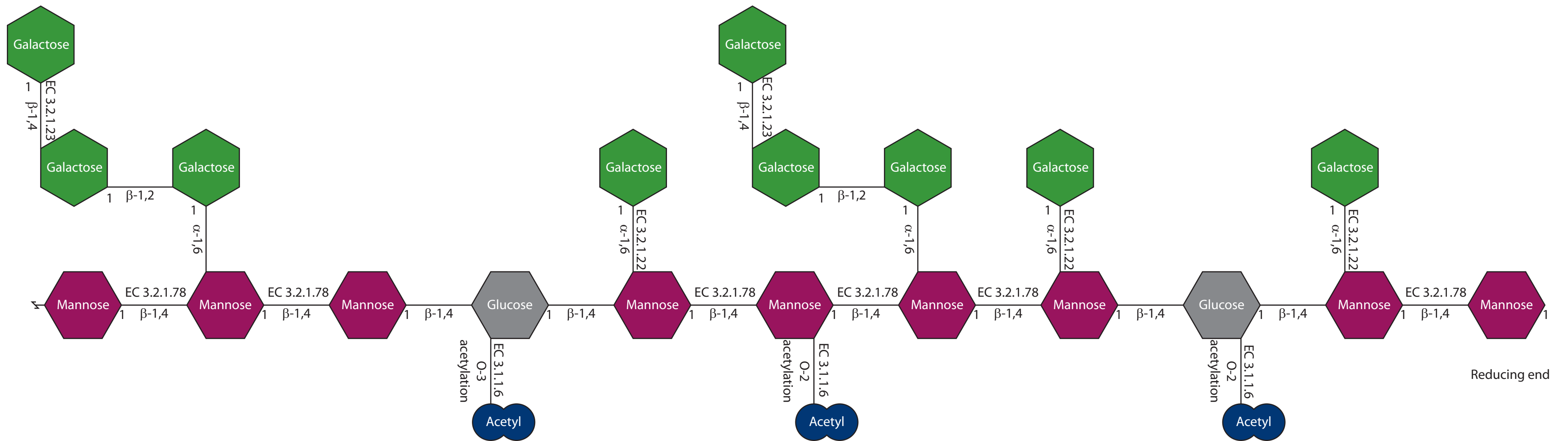

## Oligos

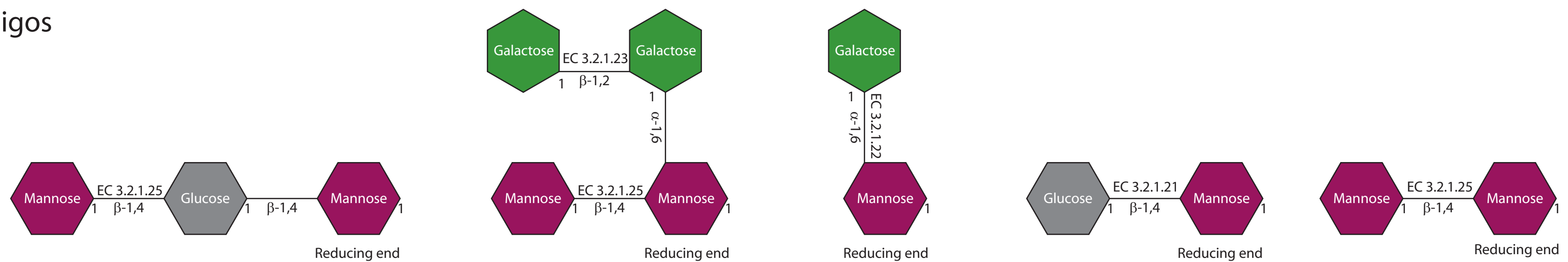

Supplement: Additional file 7: — Schematic representation of soluble galactoglucomannan. The nature of the bonds between the sugar units are noted where they are known. The number of sides on the sugar polygons reflect the number of carbons of the sugar. The first carbon is indicated on all sugars with a 1 to clarify the bond configurations. Carbons are numbered clockwise from the first carbon with the two last carbons of the sugar (hexose or pentose) on the same corner of the polygon. The oligos are hypothetical hydrolysis products and sugars that appear due to the action of exo-acting enzymes. The structure is based on ref. [18-21]. [file 1471-2164-13-313-S7.pdf]

# Smooth pectin

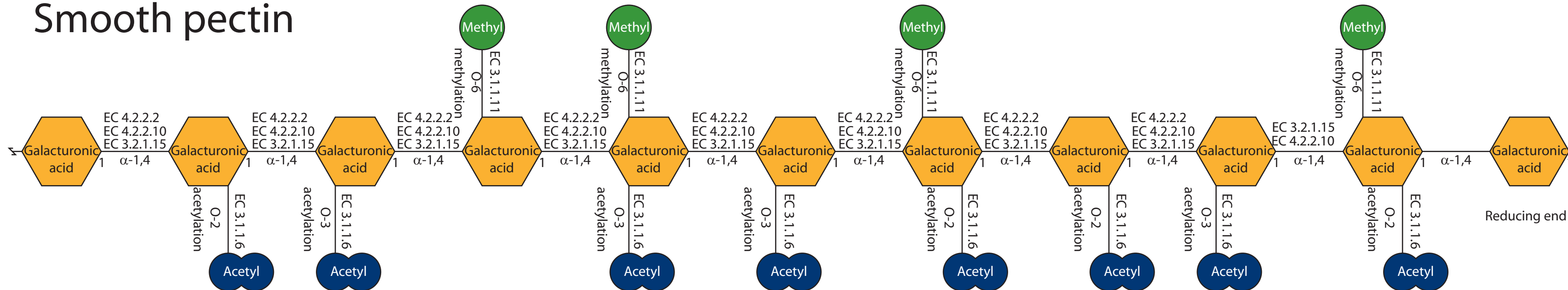

## Oligos

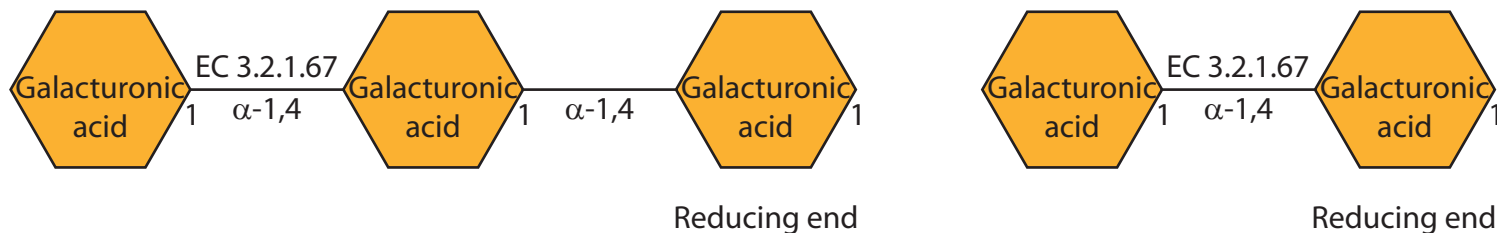

Supplement: Additional file 8: — Schematic representation of smooth pectin. The nature of the bonds between the sugar units are noted where they are known. The number of sides on the sugar polygons reflect the number of carbons of the sugar. The first carbon is indicated on all sugars with a 1 to clarify the bond configurations. Carbons are numbered clockwise from the first carbon with the two last carbons of the sugar (hexose or pentose) on the same corner of the polygon. The oligos are hypothetical hydrolysis products and sugars that appear due to the action of exo-acting enzymes. The structure is based on refs. [20,60]. [file 1471-2164-13-313-S8.pdf]

# Xylogalacturonan

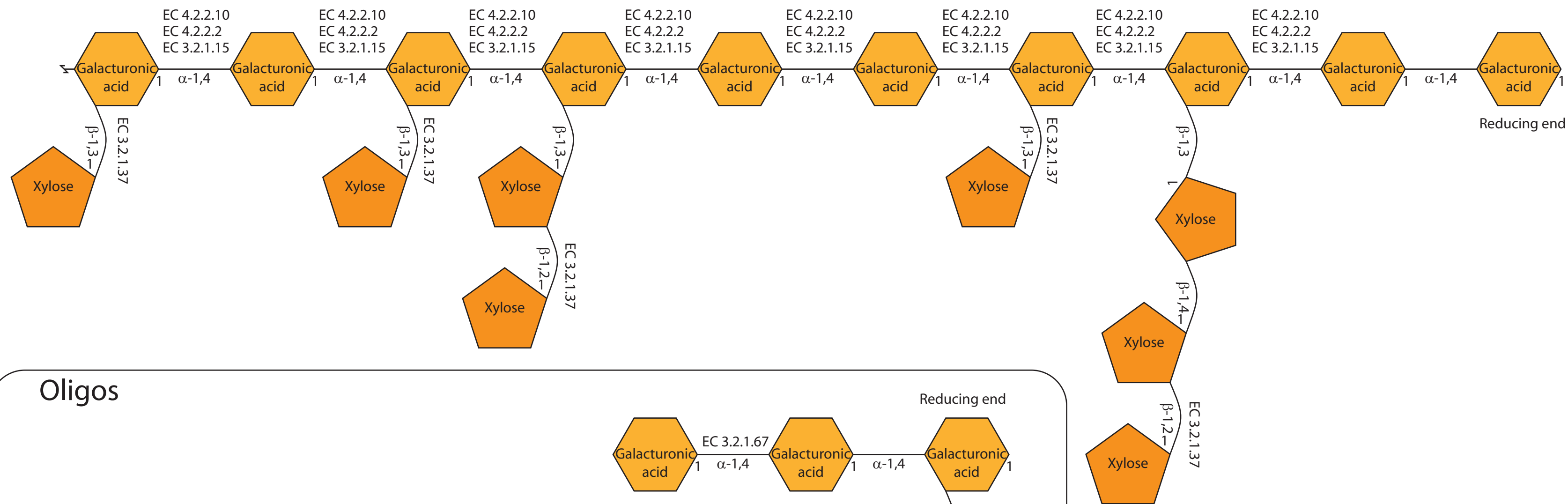

## Oligos

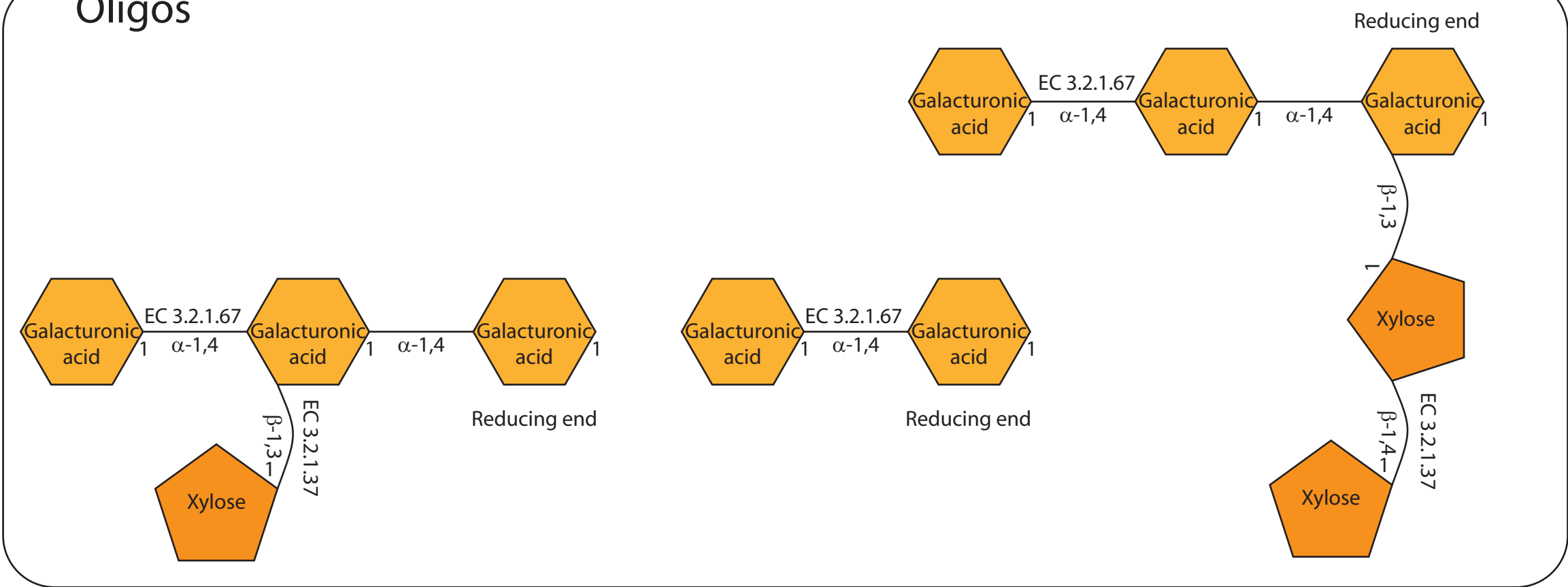

Supplement: Additional file 9: — Schematic representation of xylogalactouronan. The nature of the bonds between the sugar units are noted where they are known. The number of sides on the sugar polygons reflect the number of carbons of the sugar. The first carbon is indicated on all sugars with a 1 to clarify the bond configurations. Carbons are numbered clockwise from the first carbon with the two last carbons of the sugar (hexose or pentose) on the same corner of the polygon. The oligos are hypothetical hydrolysis products and sugars that appear due to the action of exo-acting enzymes. The structure is based on refs. [20,64-67]. [file 1471-2164-13-313-S9.pdf]

Xylan

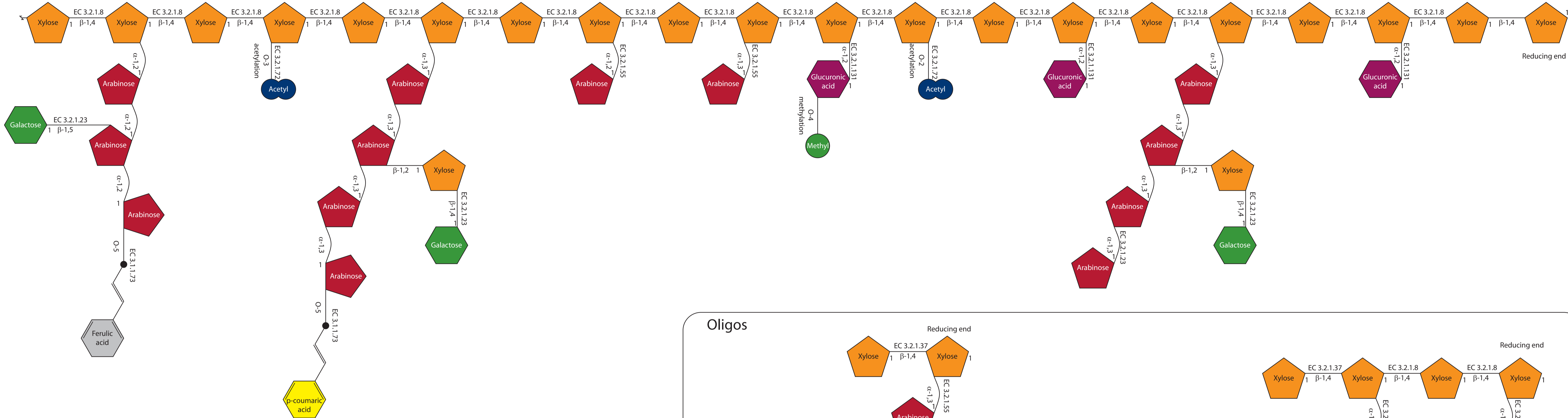

Oligos

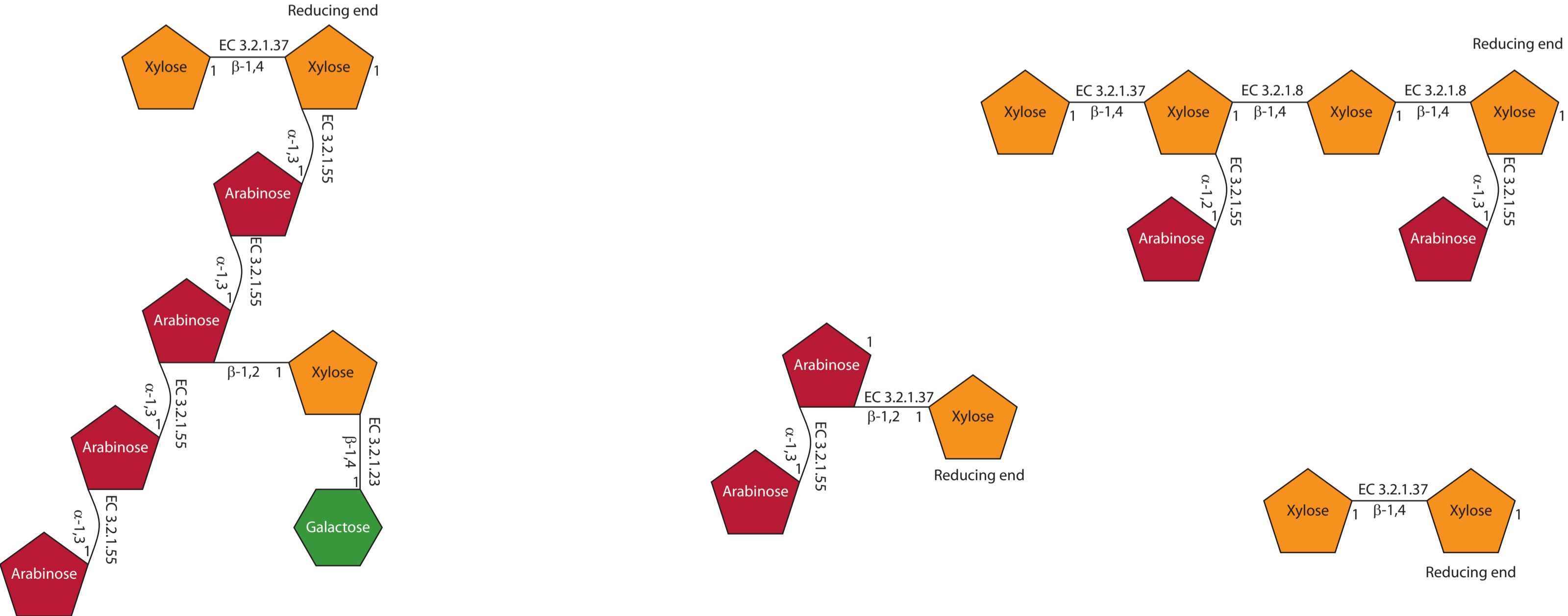

Supplement: Additional file 10: — Schematic representation of xylan. The nature of the bonds between the sugar units are noted where they are known. The number of sides on the sugar polygons reflect the number of carbons of the sugar. The first carbon is indicated on all sugars with a 1 to clarify the bond configurations. Carbons are numbered clockwise from the first carbon with the two last carbons of the sugar (hexose or pentose) on the same corner of the polygon. The oligos are hypothetical hydrolysis products and sugars that appear due to the action of exo-acting enzymes. The structure is based on refs. [20,60,68,69]. [file 1471-2164-13-313-S10.pdf]

# Xyloglucan type XXGG

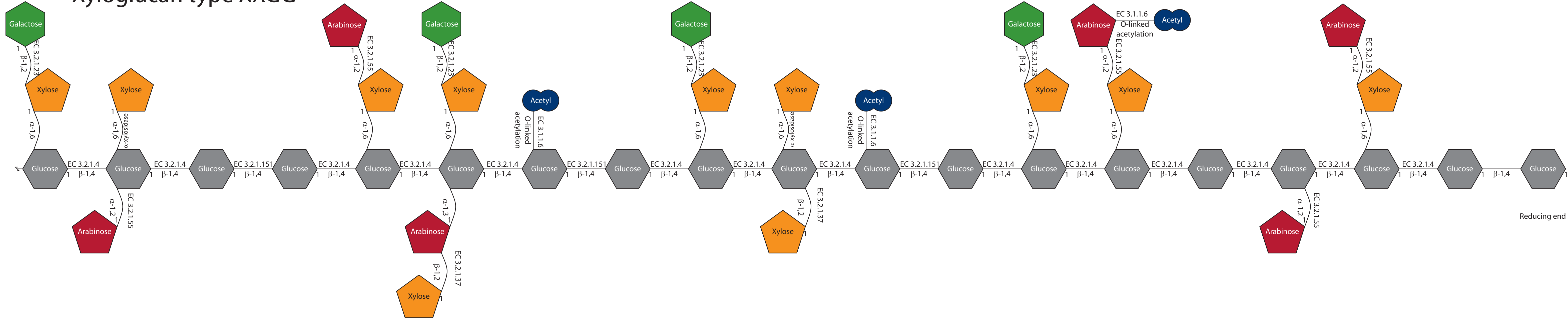

## Oligos

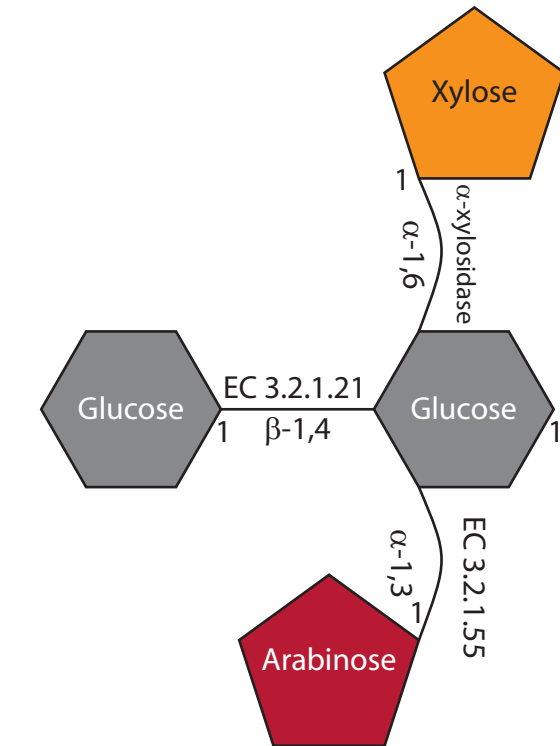

Supplement: Additional file 11: — Schematic representation of xyloglucan type XXGG. The nature of the bonds between the sugar units are noted where they are known. The number of sides on the sugar polygons reflect the number of carbons of the sugar. The first carbon is indicated on all sugars with a 1 to clarify the bond configurations. Carbons are numbered clockwise from the first carbon with the two last carbons of the sugar (hexose or pentose) on the same corner of the polygon. The oligos are hypothetical hydrolysis products and sugars that appear due to the action of exo-acting enzymes. The structure is based on refs. [20,70,71]. [file 1471-2164-13-313-S11.pdf]

Xyloglucan type XXXG

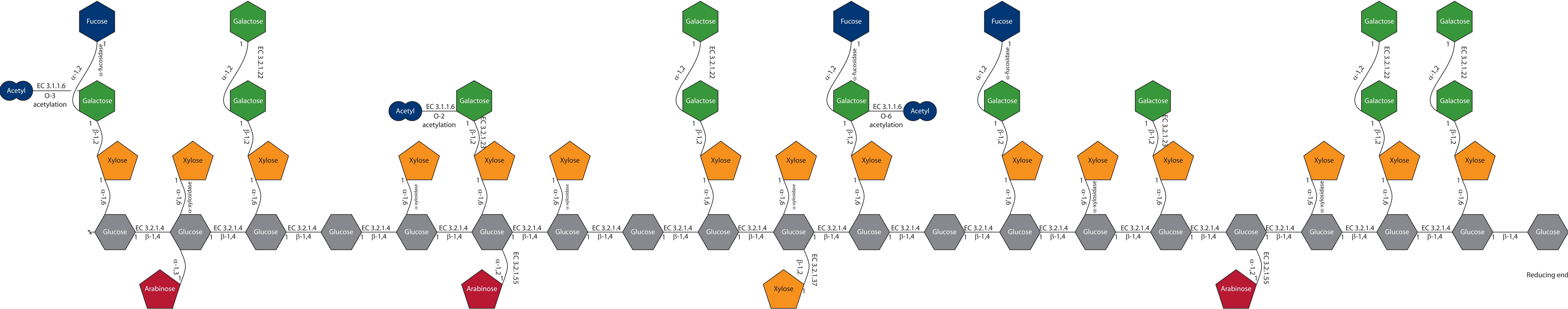

Supplement: Additional file 12: — Schematic representation of xyloglucan type XXXG. The nature of the bonds between the sugar units are noted where they are known. The number of sides on the sugar polygons reflect the number of carbons of the sugar. The first carbon is indicated on all sugars with a 1 to clarify the bond configurations. Carbons are numbered clockwise from the first carbon with the two last carbons of the sugar (hexose or pentose) on the same corner of the polygon. The oligos are hypothetical hydrolysis products and sugars that appear due to the action of exo-acting enzymes. The structure is based on refs. [20,70,71]. [file 1471-2164-13-313-S12.pdf]

## Arabinogalactan (type I)

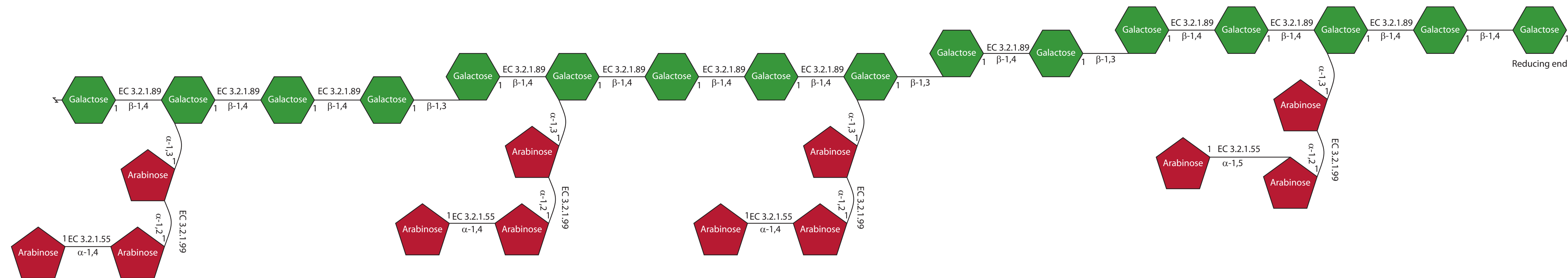

## Oligos

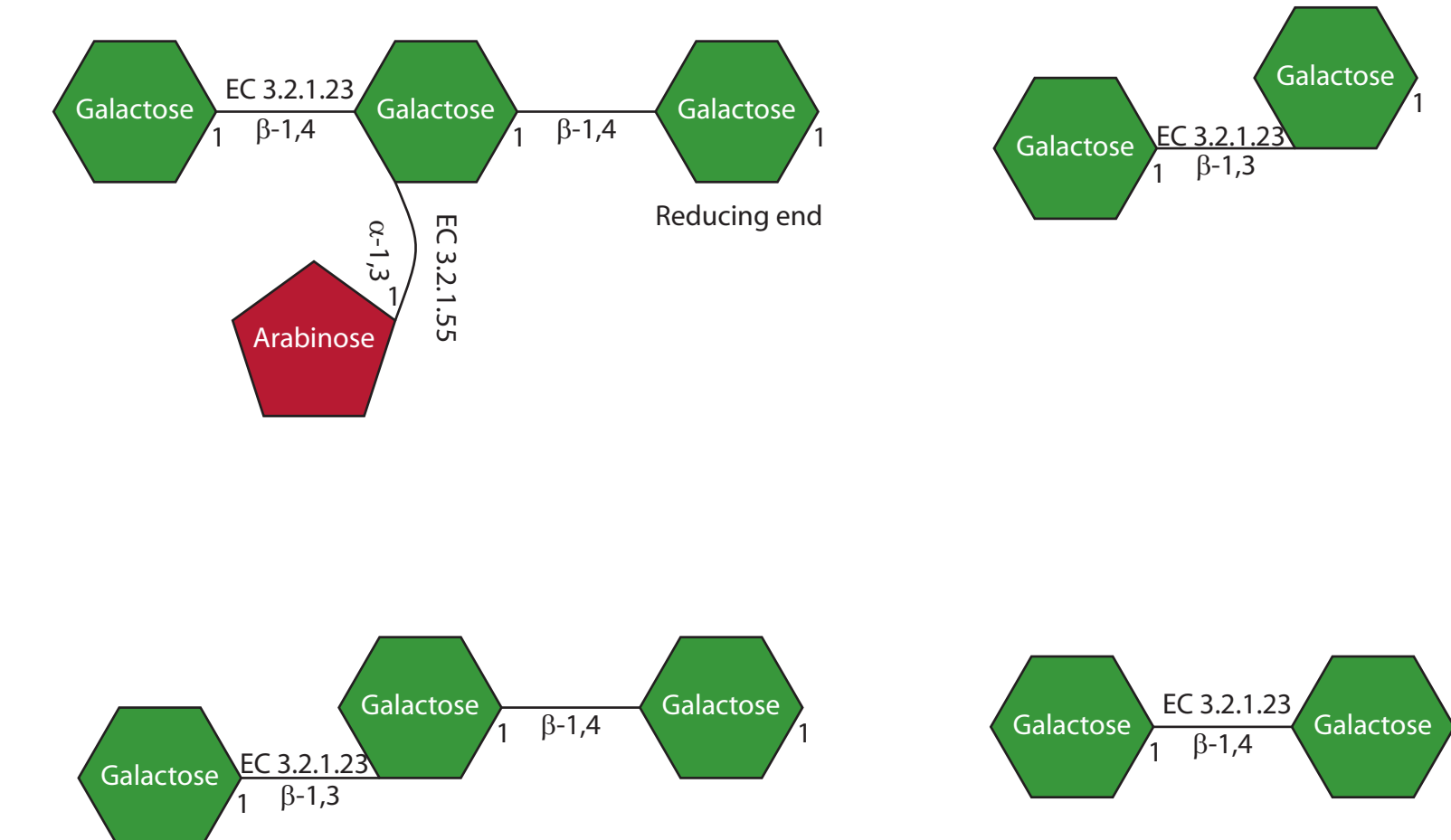

Supplement: Additional file 13: — Schematic representation of arabinogalactan type I. The nature of the bonds between the sugar units are noted where they are known. The number of sides on the sugar polygons reflect the number of carbons of the sugar. The first carbon is indicated on all sugars with a 1 to clarify the bond configurations. Carbons are numbered clockwise from the first carbon with the two last carbons of the sugar (hexose or pentose) on the same corner of the polygon. The oligos are hypothetical hydrolysis products and sugars that appear due to the action of exo-acting enzymes. The structure is based on refs. [12,72,73]. [file 1471-2164-13-313-S13.pdf]

Arabinogalactan (type II)

Oligos

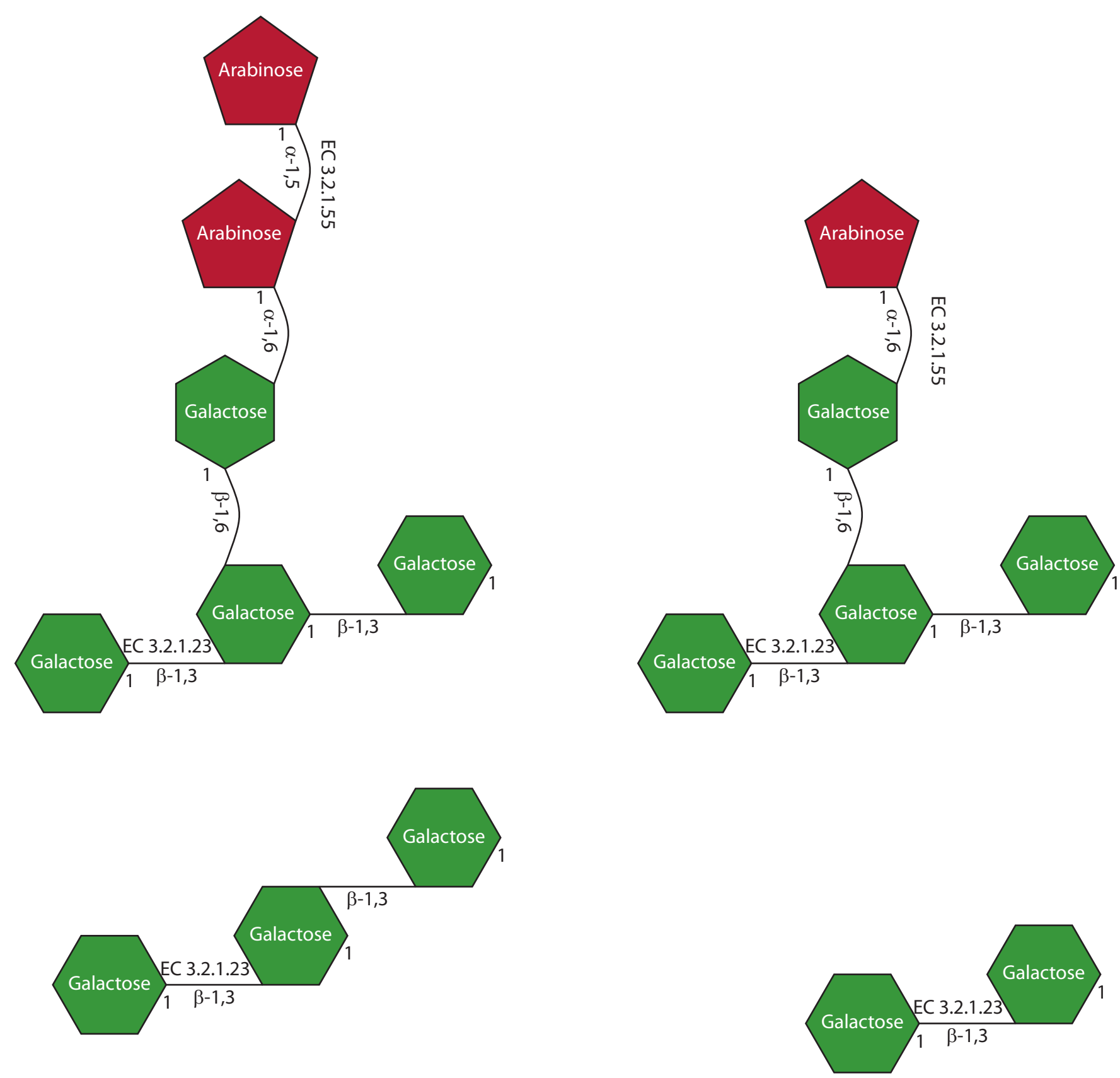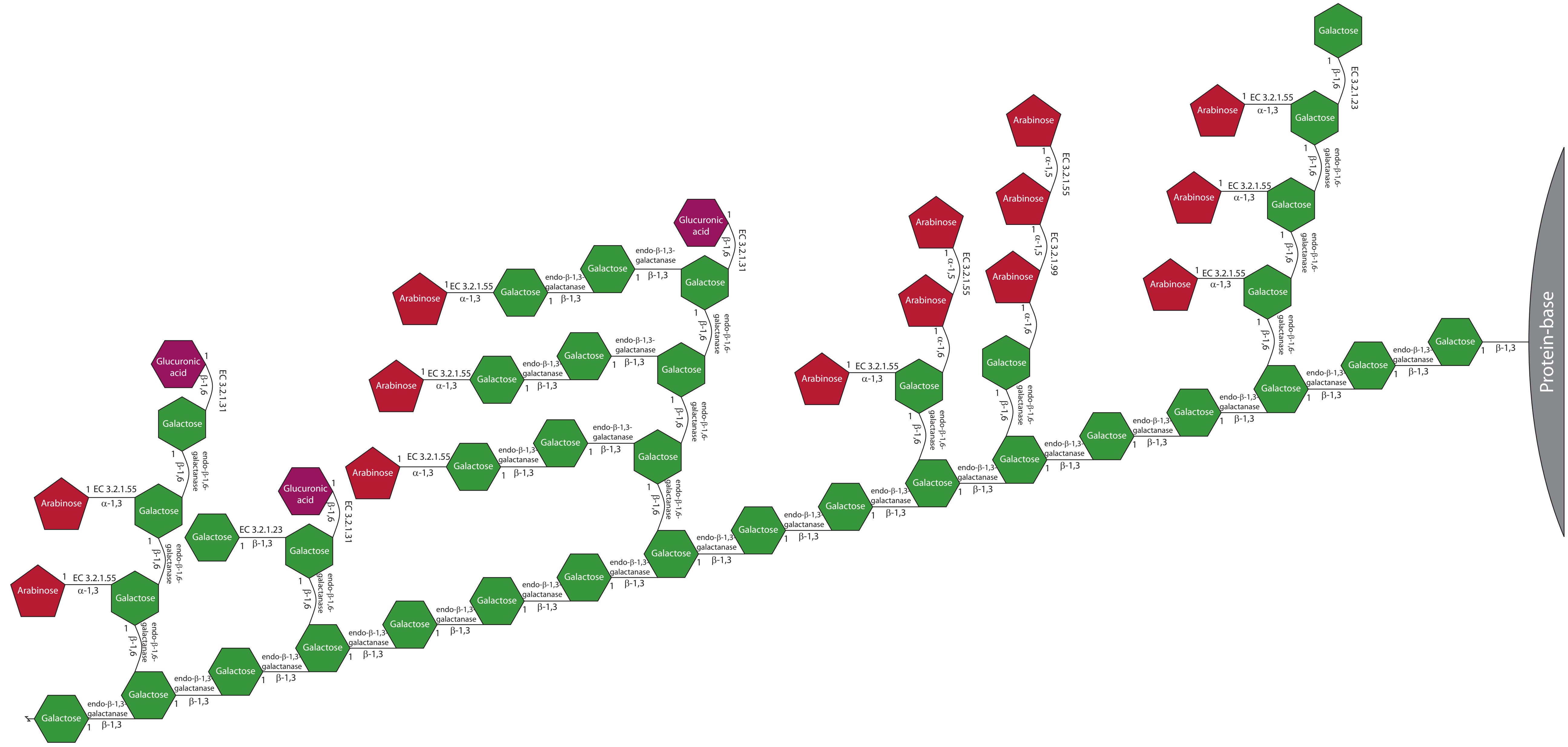

Protein-base

Supplement: Additional file 14: — Schematic representation of arabinogalactan type II (protein bound arabinogalactan). The nature of the bonds between the sugar units are noted where they are known. The number of sides on the sugar polygons reflect the number of carbons of the sugar. The first carbon is indicated on all sugars with a 1 to clarify the bond configurations. Carbons are numbered clockwise from the first carbon with the two last carbons of the sugar (hexose or pentose) on the same corner of the polygon. The oligos are hypothetical hydrolysis products and sugars that appear due to the action of exo-acting enzymes. The structure is based on [12,72,74,75]. [file 1471-2164-13-313-S14.pdf]

Arabinan

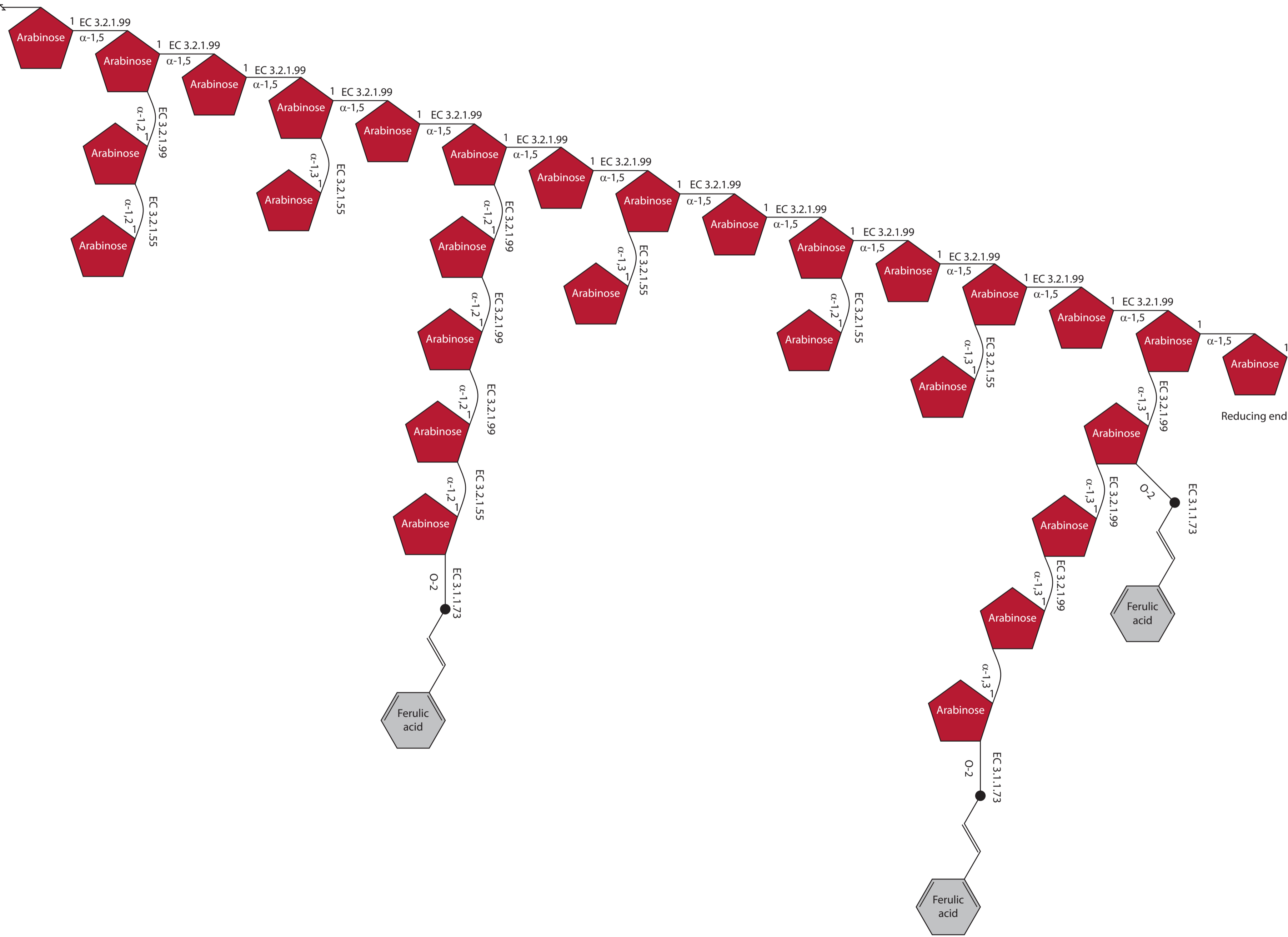

Oligos

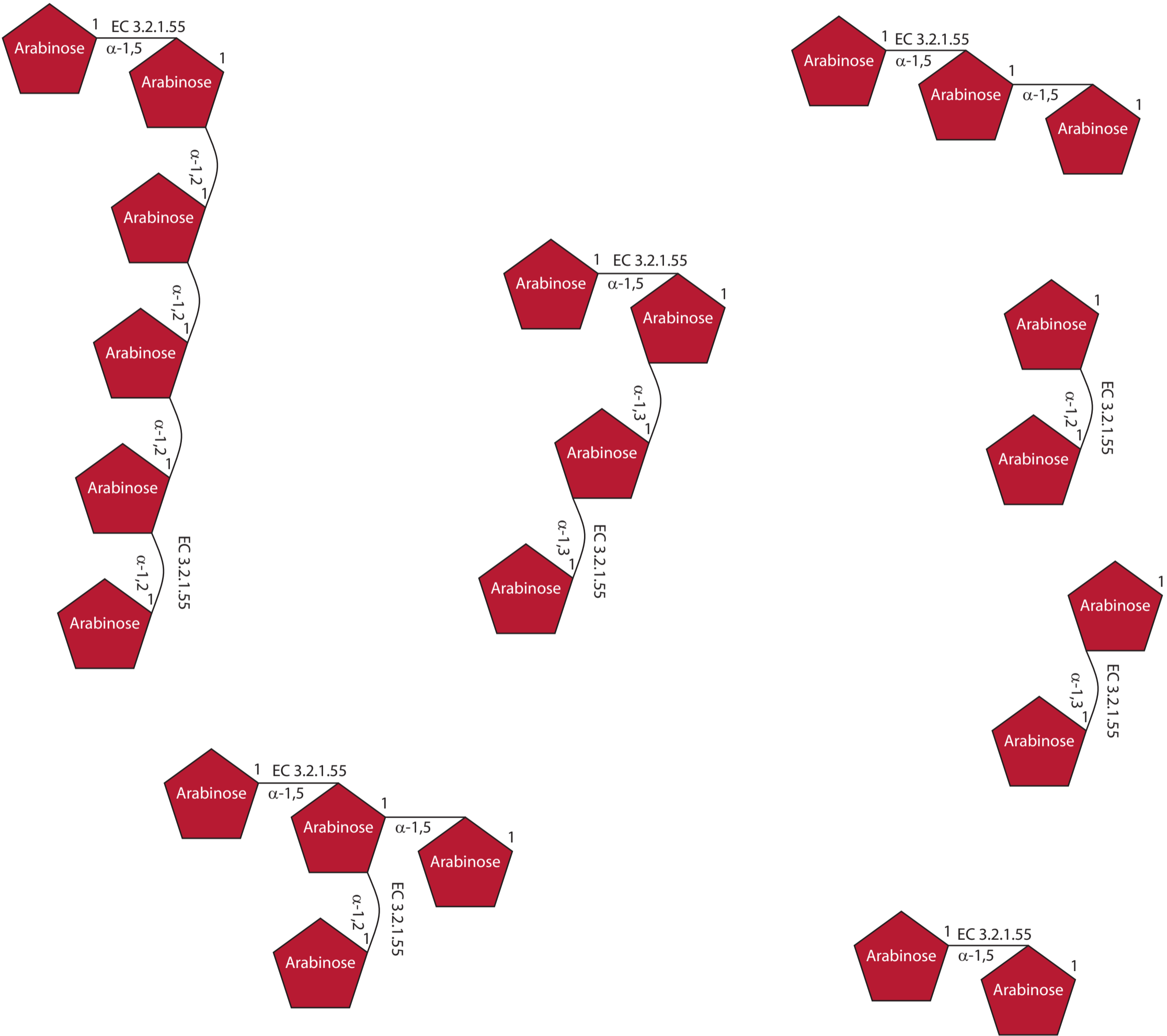

Supplement: Additional file 15: — Schematic representation of arabinan. The nature of the bonds between the sugar units are noted where they are known. The number of sides on the sugar polygons reflect the number of carbons of the sugar. The first carbon is indicated on all sugars with a 1 to clarify the bond configurations. Carbons are numbered clockwise from the first carbon with the two last carbons of the sugar (hexose or pentose) on the same corner of the polygon. The oligos are hypothetical hydrolysis products and sugars that appear due to the action of exo-acting enzymes. The structure is based on refs. [76,77,78]. [file 1471-2164-13-313-S15.pdf]

## Rhamnogalacturonan I

## Arabinan sidechains

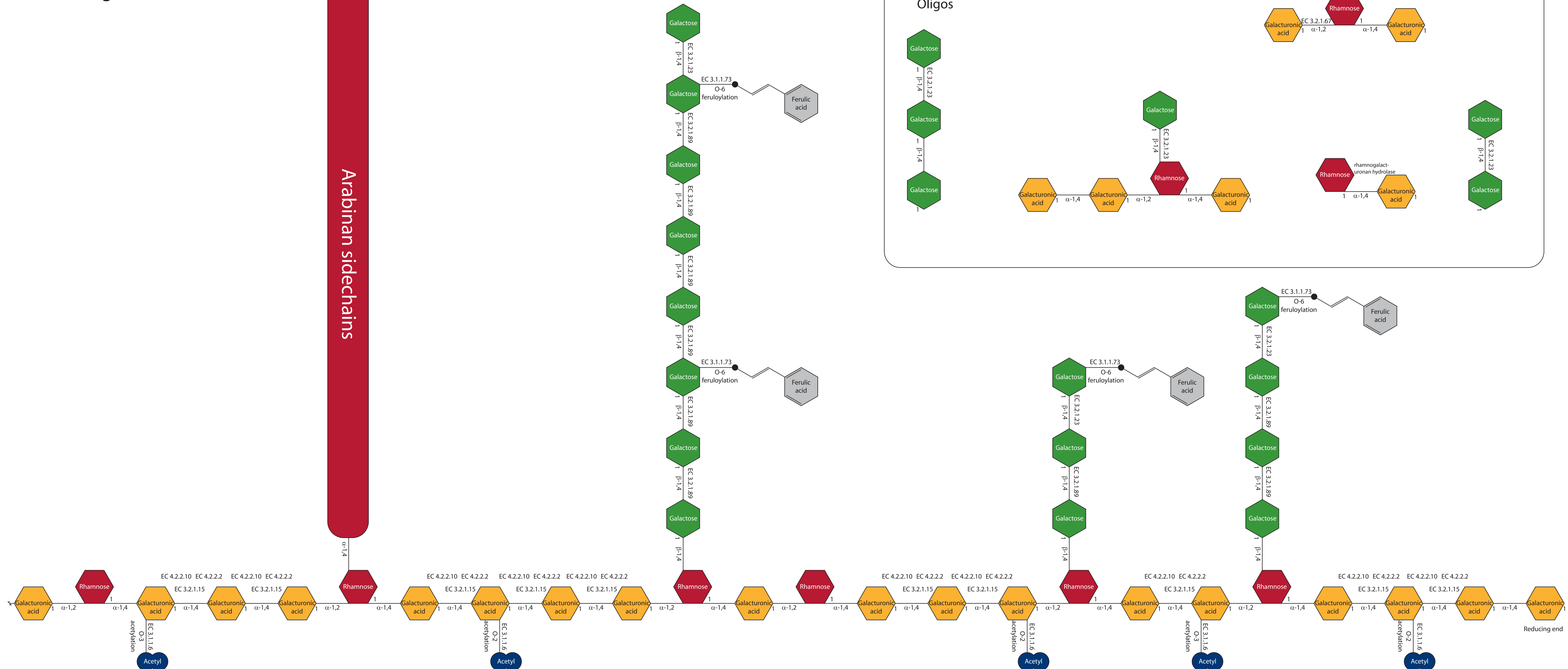

Supplement: Additional file 16: — Schematic representation of rhamnogalacturonan type I. The nature of the bonds between the sugar units are noted where they are known. The number of sides on the sugar polygons reflect the number of carbons of the sugar. The first carbon is indicated on all sugars with a 1 to clarify the bond configurations. Carbons are numbered clockwise from the first carbon with the two last carbons of the sugar (hexose or pentose) on the same corner of the polygon. The oligos are hypothetical hydrolysis products and sugars that appear due to the action of exo-acting enzymes. The structure is based on refs. [20,76,77,79,80]. [file 1471-2164-13-313-S16.pdf]

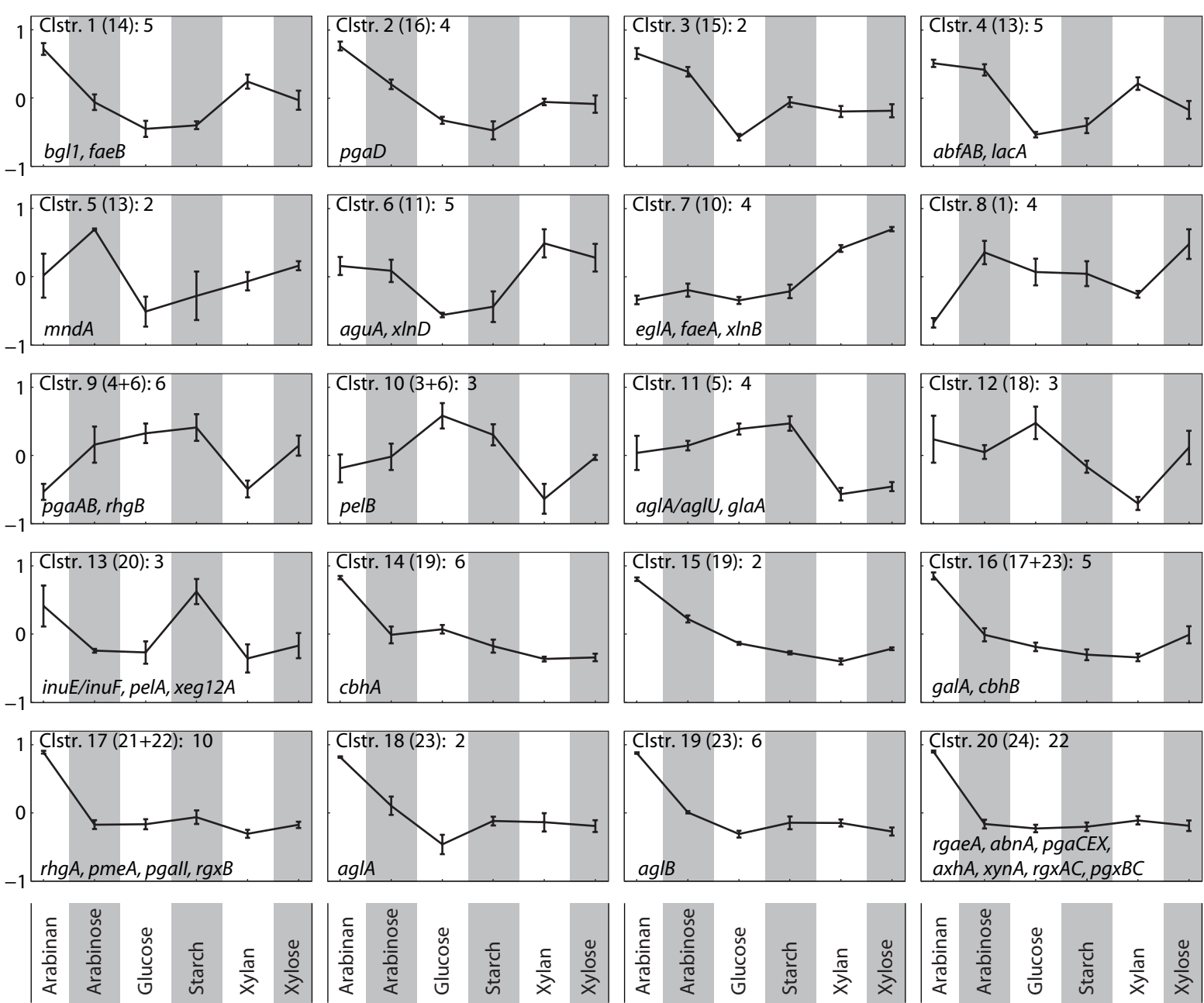

Supplement: Additional file 23: — Clustering of 103 significantly regulated genes coding for enzymes with putative and characterized polysaccharide-activities. All genes were found to be significantly regulated in at least one pairwise-comparison of two carbon sources. Cluster numbers in parentheses is the number of the corresponding cluster(s) in Figure 2. The number of genes in each cluster is shown next to the cluster number. The gene names of known genes found in a particular cluster are found at the bottom of the transcription profile graph of each cluster. The genes were clustered using the ClusterLustre algorithm [50]. [file 1471-2164-13-313-S23.pdf]
