# Supplementary material for: Mapping the polysaccharide degradation potential of Aspergillus niger
Source: BMC Genomics. 2012 Jul 16;13:313. doi: 10.1186/1471-2164-13-313 (PMC3542576; doi:10.1186/1471-2164-13-313)
Supplement: Additional file 18: — Full map of 16 polysaccharide structures with the addition of genes from A. niger ATCC 1015 specific for each type of bond. The nature of the bonds between the sugar units are noted where they are known. The number of sides on the sugar polygons reflect the number of carbons of the sugar. The first carbon is indicated on all sugars with a 1 to clarify the bond configurations. Carbons are numbered clockwise from the first carbon with the two last carbons of the sugar (hexose or pentose) on the same corner of the polygon. The oligos are hypothetical hydrolysis products and sugars that appear due to the action of exo-acting enzymes. Type and approximate 2D orientation is shown for each of the chemical bonds connecting sugars and/or acids. Next to each bond, one can find verified and hypothetical enzymes in A. niger ATCC 1015 capable of catalyzing the hydrolysis of the bond. The bottom panel denotes CAZy-families for each of the genes. See Additional Files 1-16 for details on literature references for structures. [file 1471-2164-13-313-S18.pdf]

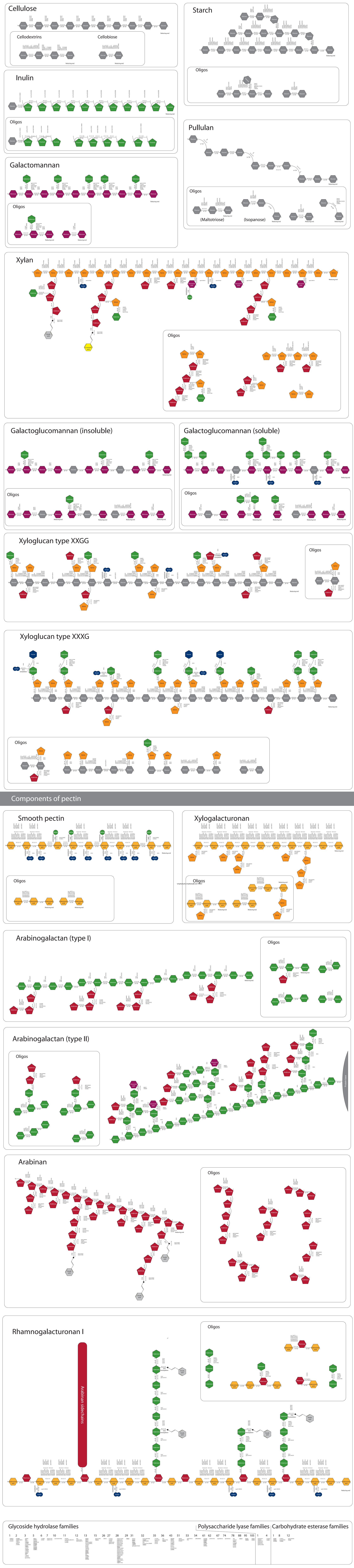

Cellulose
Cellodextrins
Cellobiose
Starch
Oligos
Inulin
Oligos
Pullulan
Oligos
(Maltotriose)
(Isopanose)
Galactomannan
Oligos
Xylan
Oligos
Galactoglucomannan (insoluble)
Oligos
Galactoglucomannan (soluble)
Oligos
Xyloglucan type XXGG
Oligos
Xyloglucan type XXXG
Oligos
Components of pectin
Smooth pectin
Oligos
Xylogalacturonan
Oligos
Arabinogalactan (type I)
Oligos
Arabinogalactan (type II)
Oligos
Arabinan
Oligos
Rhamnogalacturonan I
Arabinan sidechains
Oligos
Glycoside hydrolase families
Polysaccharide lyase families
Carbohydrate esterase families

Legend Suppl file 23 - Full map of 16 polysaccharide structures with the addition of genes from *A. niger* ATCC 1015 specific for each type of bond.

The nature of the bonds between the sugar units are noted where they are known. The number of sides on the sugar polygons reflect the number of carbons of the sugar. The first carbon is indicated on all sugars with a 1 to clarify the bond configurations. Carbons are numbered clockwise from the first carbon with the two last carbons of the sugar (hexose or pentose) on the same corner of the polygon. The oligos are hypothetical hydrolysis products and sugars that appear due to the action of exo-acting enzymes. Type and approximate 2D orientation is shown for each of the chemical bonds connecting sugars and/or acids. Next to each bond, one can find verified and hypothetical enzymes in *A. niger* ATCC 1015 capable of catalyzing the hydrolysis of the bond. The bottom panel denotes CAZy-families for each of the genes. See Additional Files 1-16 for details on literature references for structures.
