# Supplementary material for: Mapping the polysaccharide degradation potential of Aspergillus niger
Source: BMC Genomics. 2012 Jul 16;13:313. doi: 10.1186/1471-2164-13-313 (PMC3542576; doi:10.1186/1471-2164-13-313)
Supplement: Additional file 19: — Map of statistically significantly regulated genes coding for carbohydrate-active enzymes in a pairwise comparison of growth on arabinan versus L-arabinose. Genes marked with a red box are significantly up-regulated on arabinan relative to L-arabinose, a green box denotes the opposite, gray boxes means that the gene is found in the A. niger CBS 513.88 sequence, but no bi-directional best hit is found in the A. niger ATCC 1015 sequence. The absence of a box mean that this gene is not statistically significantly regulated in this comparison. [file 1471-2164-13-313-S19.pdf]

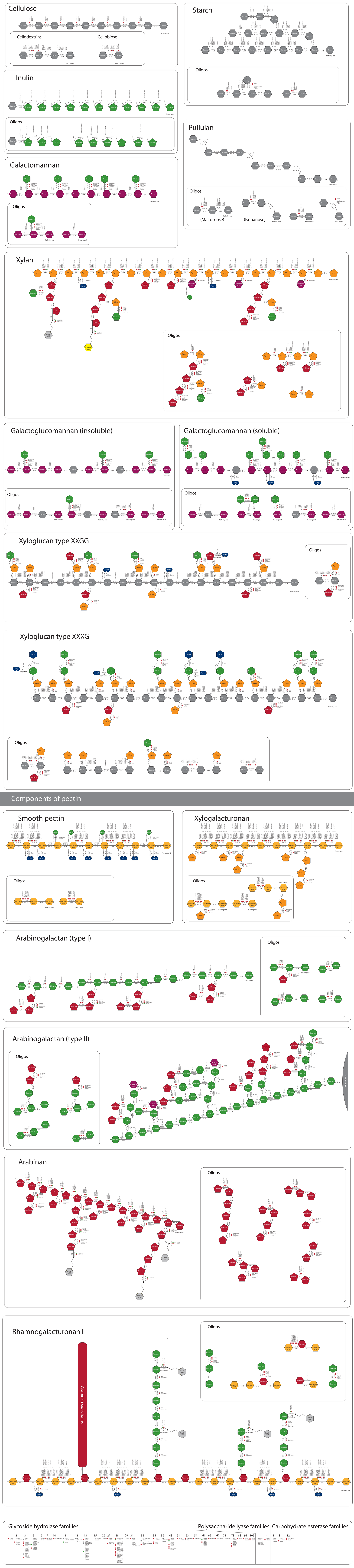

Cellulose
Cellodextrins
Cellobiose
Starch
Oligos
Inulin
Oligos
Pullulan
Oligos
(Maltotriose)
(Isopanose)
Galactomannan
Oligos
Xylan
Oligos
Galactoglucomannan (insoluble)
Oligos
Galactoglucomannan (soluble)
Oligos
Xyloglucan type XXGG
Oligos
Xyloglucan type XXXG
Oligos
Components of pectin
Smooth pectin
Oligos
Xylogalacturonan
Oligos
Arabinogalactan (type I)
Oligos
Arabinogalactan (type II)
Oligos
Arabinan
Oligos
Rhamnogalacturonan I
Oligos
Arabinan sidechains
Glycoside hydrolase families
Polysaccharide lyase families
Carbohydrate esterase families

Legend Suppl file 17 - Map of statistically significantly regulated genes coding for carbohydrate-active enzymes in a pairwise comparison of growth on arabinan versus L-arabinose

Genes marked with a red box are significantly up-regulated on arabinan relative to *L*-arabinose, a green box denotes the opposite, gray boxes means that the gene is found in the *A. niger* CBS 513.88 sequence, but no bi-directional best hit is found in the *A. niger* ATCC 1015 sequence. The absence of a box mean that this gene is not statistically significantly regulated in this comparison.
