# Supplementary material for: Mapping the polysaccharide degradation potential of Aspergillus niger
Source: BMC Genomics. 2012 Jul 16;13:313. doi: 10.1186/1471-2164-13-313 (PMC3542576; doi:10.1186/1471-2164-13-313)
Supplement: Additional file 24: — 24 maps of enzymatic activities present in the expression-based clusters 1-24 of Figure 2. Genes marked with a red box are found in the cluster, gray boxes means that the gene is found in the A. niger CBS 513.88 sequence, but no bi-directional best hit is found in the A. niger ATCC 1015 sequence. [file Cluster18.pdf › Cluster18.pdf]

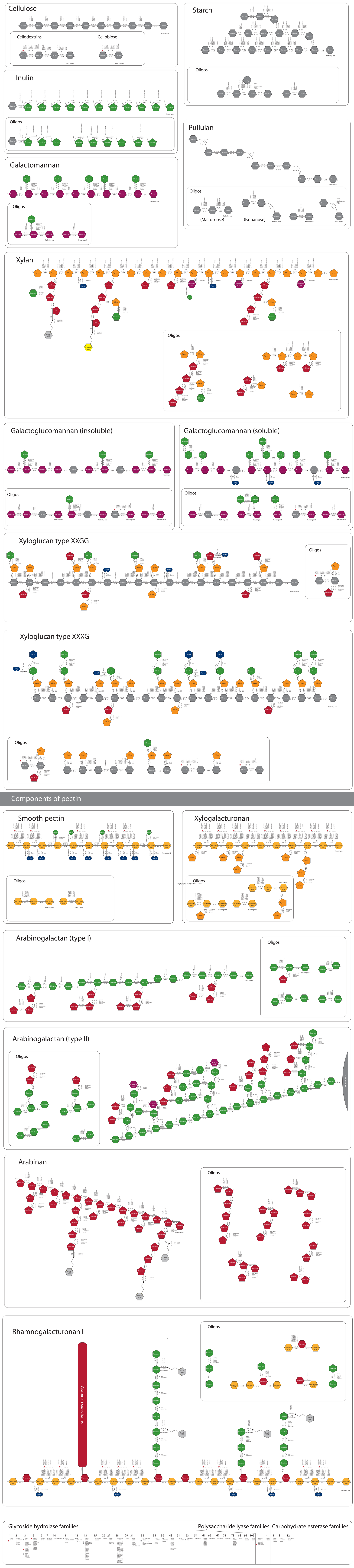
Cellulose
Cellodextrins
Cellobiose
Starch
Oligos
Inulin
Oligos
Pullulan
Oligos
(Maltotriose)
(Isopanose)
Galactomannan
Oligos
Xylan
Oligos
Galactoglucomannan (insoluble)
Oligos
Galactoglucomannan (soluble)
Oligos
Xyloglucan type XXGG
Oligos
Xyloglucan type XXXG
Oligos
Components of pectin
Smooth pectin
Oligos
Xylogalacturonan
Oligos
Arabinogalactan (type I)
Oligos
Arabinogalactan (type II)
Oligos
Arabinan
Oligos
Rhamnogalacturonan I
Arabinan sidechains
Oligos
Glycoside hydrolase families
Polysaccharide lyase families
Carbohydrate esterase families
