# Supplementary material for: Mapping the polysaccharide degradation potential of Aspergillus niger
Source: BMC Genomics. 2012 Jul 16;13:313. doi: 10.1186/1471-2164-13-313 (PMC3542576; doi:10.1186/1471-2164-13-313)
Supplement: Additional file 17: — Tables of enzymatic activities required for the degradation of 16 different plant-derived polysaccharides. For each isoenzyme, putative or characterized, is noted literature references and the gene ID in the whole genome sequencings of A. niger CBS 513.88 and ATCC 1015 [3-7,10-12,14,18-36,39-41,45-49,51,59-68,70-226]. [file 1471-2164-13-313-S17.pdf]

Table 1: Enzymatic activities required for the degradation of starch by *A. niger*. For each isoenzyme, putative or characterized, is noted literature references and the gene ID in the sequencings of *A. niger* CBS 513.88 and ATCC 1015. Genes for *amyA* and *amyB* are not noted in the ATCC sequence list, as three almost identical genes exist *amyA*, *amyB* and ORF 140567. As the genes in the ATCC 1015 sequence are found by bi-directional best hits, it is, due to sequence differences between the two strains, not possible to determine 1:1 relationships for all three genes. However, ORF 140567 can be used as indicative for all of them. The list of necessary enzymes is gathered from [59].

EC number	Name	Gene characterization	Specificity	**Gene**	**Uniprot**	**CBS 513.88**	**ATCC 1015**
3.2.1.3	glucoamylase (exo-1,4-glucosidase/amyloglucosidase)					An02g00850	206445
3.2.1.3	glucoamylase (exo-1,4-glucosidase/amyloglucosidase)					An02g06950	37060
3.2.1.3	glucoamylase (exo-1,4-glucosidase/amyloglucosidase)	[100–202]		*glaA*	P69328	An03g06550	213597
3.2.1.1	α-amylase					An04g06930	45304
3.2.1.1	α-amylase	[155]		*amyA/amyB*	P56271	An05g02100	N/A
3.2.1.1	α-amylase					An09g03100	188489
3.2.1.1	α-amylase	[155]		*amyA/amyB*	P56271	An12g06930	N/A
3.2.1.1	α-amylase					An09g03110	122069
3.2.1.1	α-amylase					An11g03340	140567
3.2.1.1	α-amylase					An12g02460	57002
3.2.1.1	α-amylase					An15g07800	182162
3.2.1.41	α-1,4-pullulanase	[154,191]					
3.2.1.3	glucoamylase (exo-1,4-glucosidase/amyloglucosidase)	[92]				An02g00850	206445
3.2.1.3	glucoamylase (exo-1,4-glucosidase/amyloglucosidase)					An02g06950	37060
3.2.1.3	glucoamylase (exo-1,4-glucosidase/amyloglucosidase)	[92,100–202]		*glaA*	P69328	An03g06550	213597
3.2.1.20	α-glucosidase					An13g03710	50927
3.2.1.20	α-glucosidase					An01g04880	55419
3.2.1.20	α-glucosidase	[177]		*aglA/aglU*	P56526	An04g06920	214233
3.2.1.20	α-glucosidase					An07g00350	40261
3.2.1.20	α-glucosidase					An09g05880	128654
3.2.1.3	glucoamylase (exo-1,4-glucosidase/amyloglucosidase)					An02g00850	206445
3.2.1.3	glucoamylase (exo-1,4-glucosidase/amyloglucosidase)					An02g06950	37060
3.2.1.3	glucoamylase (exo-1,4-glucosidase/amyloglucosidase)	[100–202]		*glaA*	P69328	An03g06550	213597

Table 2: Enzymatic activities required for the degradation of cellulose by *A. niger*. For each isoenzyme, putative or characterized, is noted literature references and the gene ID in the sequencings of *A. niger* CBS 513.88 and ATCC 1015. The list of necessary enzymes is gathered from the work by [19,60].

EC number	Name	Gene characterization	Specificity	**Gene**	**Uniprot**	**CBS 513.88**	**ATCC 1015**
3.2.1.91	Cellulose 1,4-β-cellobiosidase (Cellobiohydrolase)	[51]		*cbhA*	Q9UVS9	An07g09330	53159
3.2.1.91	Cellulose 1,4-β-cellobiosidase (Cellobiohydrolase)	[51]		*cbhB*	Q9UVS8	An01g11660	51773
3.2.1.91	Cellulose 1,4-β-cellobiosidase (Cellobiohydrolase)					An12g02220	54490
3.2.1.4	endo-glucanase	[41,139,140,150]	[134]	*eglA*	O74705	An14g02760	211053
3.2.1.4	endo-glucanase	[41,87,138,150]	[134]	*eglB*	O74706	An07g08950	209376
3.2.1.4	endo-glucanase					An03g01050	194447
3.2.1.4	endo-glucanase					An03g05380	N/A
3.2.1.4	endo-glucanase					An03g05530	191511
3.2.1.4	endo-glucanase					An04g08550	194765
3.2.1.4	endo-glucanase					An08g01760	133986
3.2.1.4	endo-glucanase					An15g04900	182430
3.2.1.21	β-glucosidase					An03g03740	213437
3.2.1.21	β-glucosidase					An03g05330	44520
3.2.1.21	β-glucosidase					An04g03170	131747
3.2.1.21	β-glucosidase					An06g02040	176601
3.2.1.21	β-glucosidase					An07g07630	139037
3.2.1.21	β-glucosidase					An07g09760	N/A
3.2.1.21	β-glucosidase					An08g08240	38077
3.2.1.21	β-glucosidase					An11g06080	208871
3.2.1.21	β-glucosidase					An11g06090	N/A
3.2.1.21	β-glucosidase					An14g01770	210981
3.2.1.21	β-glucosidase					An15g01890	182309
3.2.1.21	β-glucosidase					An15g04800	181816
3.2.1.21	β-glucosidase					An17g00520	129891
3.2.1.21	β-glucosidase	[30–36,137,223]		*bgl1*	A2RAL4	An18g03570	56782

Table 3: Enzymatic activities required for the degradation of pullulan by *A. niger*. For each isoenzyme, putative or characterized, is noted literature references and the gene ID in the sequencings of *A. niger* CBS 513.88 and ATCC 1015. The list of necessary enzymes is gathered from the work by [61].

EC number	Name	Gene characterization	Specificity	**Gene**	**Uniprot**	**CBS 513.88**	**ATCC 1015**
3.2.1.57	isopullulanase	[86,93,94,175,191]	[93,191]	*ipuA*	O00105	N/A	N/A
3.2.1.41	pullulanase	[154]	[94]				
3.2.1.3	glucoamylase (exo-1,4-glucosidase/amyloglucosidas					An02g00850	206445
3.2.1.3	glucoamylase (exo-1,4-glucosidase/amyloglucosidas					An02g06950	37060
3.2.1.3	glucoamylase (exo-1,4-glucosidase/amyloglucosidas	[100–202]		*glaA*	P69328	An03g06550	213597

Table 4: Enzymatic activities required for the degradation of inulin by *A. niger*. For each isoenzyme, putative or characterized, is noted literature references and the gene ID in the sequencings of *A. niger* CBS 513.88 and ATCC 1015. The list of necessary enzymes is gathered from the work by [62].

EC number	Name	Gene characterization	Specificity	**Gene**	**Uniprot**	**CBS 513.88**	**ATCC 1015**
3.2.1.7	endo-inulinase	[7,88,178,226]		*inuA/inuB*	O74641	An11g03200	52928
3.2.1.80	exo-inulinase	[7,176]		*inuE/inuF/inu1*	Q0ZR33	An12g08280	56664
3.2.1.80	exo-inulinase	[7,176]		*inuE/inuF/inu1*	Q0ZR33	An12g08280	56664
3.2.1.26	invertase	[7,125,218]	[218]	*sucB/suc2*	Q0ZR36	An15g00320	N/A

Table 5: Enzymatic activities required for the degradation of galactomannan by *A. niger*. For each isoenzyme, putative or characterized, is noted literature references and the gene ID in the sequencings of *A. niger* CBS 513.88 and ATCC 1015. The list of necessary enzymes is gathered from the work by [19,63].

EC number	Name	Gene characterization	Specificity	**Gene**	**Uniprot**	**CBS 513.88**	**ATCC 1015**
3.2.1.78	Endo-1,4-β-D-mannanase	[22,132]	[22,132]			An05g01320	50378
3.2.1.78	Endo-1,4-β-D-mannanase					An15g07760	40875
3.2.1.25	β-mannosidase					An01g06630	172587
3.2.1.25	β-mannosidase	[23–25,132]	[23,24,132]	*mndA*	Q9UUZ3	An11g06540	138876
3.2.1.22	α-galactosidase	[24,26]	[24]	*aglC*	Q9UUZ4	An09g00260	212736
3.2.1.22	α-galactosidase					An01g01320	172232
3.2.1.22	α-galactosidase	[26,27]		*aglB*	Q9Y865	An02g11150	207264
3.2.1.22	α-galactosidase	[26,28,29]		*aglA*	A2QL72	An06g00170	37736
3.2.1.22	α-galactosidase					An11g06330	39180
3.2.1.22	α-galactosidase					An14g01800	185285

Table 6: Enzymatic activities required for the degradation of soluble galactoglucomannan by *A. niger*. For each isoenzyme, putative or characterized, is noted literature references and the gene ID in the sequencings of *A. niger* CBS 513.88 and ATCC 1015. The list of necessary enzymes is gathered from the work by [17–20].

EC number	Name	Gene characterization	Specificity	**Gene**	**Uniprot**	**CBS 513.88**	**ATCC 1015**
3.2.1.78	Endo-1,4-β-D-mannanase	[22]	[22]			An05g01320	50378
3.2.1.78	Endo-1,4-β-D-mannanase					An15g07760	40875
3.2.1.25	β-mannosidase					An01g06630	172587
3.2.1.25	β-mannosidase	[23–25]	[23,24]	*mndA*	Q9UUZ3	An11g06540	138876
3.2.1.22	α-galactosidase	[24,26]	[24]	*aglC*	Q9UUZ4	An09g00260	212736
3.2.1.22	α-galactosidase					An01g01320	172232
3.2.1.22	α-galactosidase	[26,27]		*aglB*	Q9Y865	An02g11150	207264
3.2.1.22	α-galactosidase	[26,28,29]		*aglA*	A2QL72	An06g00170	37736
3.2.1.22	α-galactosidase					An11g06330	39180
3.2.1.22	α-galactosidase					An14g01800	185285
3.2.1.21	β-glucosidase					An03g03740	213437
3.2.1.21	β-glucosidase					An03g05330	44520
3.2.1.21	β-glucosidase					An04g03170	131747
3.2.1.21	β-glucosidase					An06g02040	176601
3.2.1.21	β-glucosidase					An07g07630	139037
3.2.1.21	β-glucosidase					An07g09760	N/A
3.2.1.21	β-glucosidase					An08g08240	38077
3.2.1.21	β-glucosidase					An11g06080	208871
3.2.1.21	β-glucosidase					An11g06090	N/A
3.2.1.21	β-glucosidase					An14g01770	210981
3.2.1.21	β-glucosidase					An15g01890	182309
3.2.1.21	β-glucosidase					An15g04800	181816
3.2.1.21	β-glucosidase					An17g00520	129891
3.2.1.21	β-glucosidase	[31–36,137]		*bgl1*	A2RAL4	An18g03570	56782
3.2.1.23	β-galactosidase					An01g10350	46429
3.2.1.23	β-galactosidase					An06g00290	177434
3.2.1.23	β-galactosidase					An07g04420	180727
3.2.1.23	β-galactosidase					An14g05820	41910
3.2.1.23	β-galactosidase	[26,161]		*lacA*	P29853	An01g12150	51764
3.1.1.6	Acetyl esterase					An02g02540	N/A

Table 7: Enzymatic activities required for the degradation of insoluble galactoglucomannan by *A. niger*. For each isoenzyme, putative or characterized, is noted literature references and the gene ID in the sequencings of *A. niger* CBS 513.88 and ATCC 1015. The list of necessary enzymes is gathered from the work by [19].

EC number	Name	Gene characterization	Specificity	**Gene**	**Uniprot**	**CBS 513.88**	**ATCC 1015**
3.2.1.78	Endo-1,4-β-D-mannanase	[22]	[22]			An05g01320	50378
3.2.1.78	Endo-1,4-β-D-mannanase					An15g07760	40875
3.2.1.25	β-mannosidase					An01g06630	172587
3.2.1.25	β-mannosidase	[23–25]	[23,24]	*mndA*	Q9UUZ3	An11g06540	138876
3.2.1.22	α-galactosidase	[24,26]	[24]	*aglC*	Q9UUZ4	An09g00260	212736
3.2.1.22	α-galactosidase					An01g01320	172232
3.2.1.22	α-galactosidase	[26,27]		*aglB*	Q9Y865	An02g11150	207264
3.2.1.22	α-galactosidase	[26,28,29]		*aglA*	A2QL72	An06g00170	37736
3.2.1.22	α-galactosidase					An11g06330	39180
3.2.1.22	α-galactosidase					An14g01800	185285
3.2.1.21	β-glucosidase					An03g03740	213437
3.2.1.21	β-glucosidase					An03g05330	44520
3.2.1.21	β-glucosidase					An04g03170	131747
3.2.1.21	β-glucosidase					An06g02040	176601
3.2.1.21	β-glucosidase					An07g07630	139037
3.2.1.21	β-glucosidase					An07g09760	N/A
3.2.1.21	β-glucosidase					An08g08240	38077
3.2.1.21	β-glucosidase					An11g06080	208871
3.2.1.21	β-glucosidase					An11g06090	N/A
3.2.1.21	β-glucosidase					An14g01770	210981
3.2.1.21	β-glucosidase					An15g01890	182309
3.2.1.21	β-glucosidase					An15g04800	181816
3.2.1.21	β-glucosidase					An17g00520	129891
3.2.1.21	β-glucosidase	[30–36,137]		*bgl1*	A2RAL4	An18g03570	56782

Table 8: Enzymatic activities required for the degradation of smooth pectin by *A. niger*. For each isoenzyme, putative or characterized, is noted literature references and the gene ID in the sequencings of *A. niger* CBS 513.88 and ATCC 1015. Gene names in parentheses are genes that have not been characterized with classical molecular biological techniques, but identified from their expression profiles and sequence information. The list of necessary enzymes is gathered from the work by [19].

EC number	Name	Gene characterization	Specificity	**Gene**	**Uniprot**	**CBS 513.88**	**ATCC 1015**
4.2.2.2	pectate lyase A	[5,97]	[97]	*plyA*	Q9C2Z0	An10g00870	45021
4.2.2.10	pectin lyase	[5,133,141,164,172,192]	[207]	*pelA*	Q01172	An14g04370	41815
4.2.2.10	pectin lyase					An15g07160	210387
4.2.2.10	pectin lyase	[5,131,133]		*pelD*	P22864	An19g00270	55212
4.2.2.10	pectin lyase					An11g04030	208760
4.2.2.10	pectin lyase	[5,163,215]		*pelB*	Q00205	An03g00190	45821
3.2.1.67	exo-poly-D-galacturonase	[6]		*(rgxA)*		An01g14650	172236
3.2.1.67	exo-poly-D-galacturonase	[6]		*(rgxB)*		An03g02080	194461
3.2.1.67	exo-poly-D-galacturonase	[6]		*(pgxB)*		An03g06740	191158
3.2.1.67	exo-poly-D-galacturonase	[6]		*(pgxA)*		An11g04040	178172
3.2.1.67	exo-poly-D-galacturonase	[5,6]		*(pgaX)*		An12g07500	42184
3.2.1.67	exo-poly-D-galacturonase	[6]		*(rgxC)*		An18g04810	42917
3.2.1.15	endo-Polygalacturonase	[5,104,212]	[98,179]	*pgaI*	P26213	An01g11520	141677
3.2.1.15	endo-Polygalacturonase	[5,182]	[98]	*pgaE*	O42809	An01g14670	46255
3.2.1.15	endo-Polygalacturonase	[5,181,224]	[181]	*pgaB*	P26214	An02g04900	52219
3.2.1.15	endo-Polygalacturonase			*(pgxC)*		An02g12450	172944
3.2.1.15	endo-Polygalacturonase	[5,106]	[98]	*pgaC*	Q12554	An05g02440	43957
3.2.1.15	endo-Polygalacturonase			*(rhgC)*		An06g02070	123651
3.2.1.15	endo-Polygalacturonase	[5,183]	[183]	*pgaD*	Q9P4W2	An09g03260	50161
3.2.1.15	endo-Polygalacturonase					An09g05920	N/A
3.2.1.15	endo-Polygalacturonase	[5,201]	[201]	*rhgA*	P87160	An12g00950	189722
3.2.1.15	endo-Polygalacturonase	[5,14,105–107,147,180,194,213]	[98,165,166,179]	*pgaII*		An15g05370	182156
3.2.1.15	endo-Polygalacturonase	[5,181]	[181]	*pgaA*		An16g06990	214598
3.2.1.15	rhamnogalacturonase B	[5,201]	[201]	*rhgB*	P87161	An14g04200	211163
3.1.1.11	pectin methylesterase	[5,151,168]	[148,165,166]	*pmeA*	A2QHB7	An03g06310	44585
3.1.1.11	pectin methylesterase					An04g09690	214857
3.1.1.6	Acetyl esterase					An02g02540	N/A

Table 9: Enzymatic activities required for the degradation of xylan by *A. niger*. For each isoenzyme, putative or characterized, is noted literature references and the gene ID in the sequencings of *A. niger* CBS 513.88 and ATCC 1015. The list of necessary enzymes is gathered from the work by [19,59,68,69].

EC number	Name	Gene characterization	Specificity	**Gene**	**Uniprot**	**CBS 513.88**	**ATCC 1015**
3.2.1.8	Endo-(1,4)-β-xylanase					An01g14600	171269
3.2.1.8	Endo-(1,4)-β-xylanase	[158,209]		*xynA*	P55329	An03g00940	57436
3.2.1.8	Endo-(1,4)-β-xylanase					An11g03120	38924
3.2.1.8	Endo-(1,4)-β-xylanase	[136,203]		*exlA*		An14g07390	N/A
3.2.1.8	Endo-(1,4)-β-xylanase					An15g04550	183088
3.2.1.8	Endo-(1,4)-β-xylanase	[41,152,159,210]	[159]	*xynB/xlnB/xylB*	P55330	An01g00780	52071
3.2.1.37	Xylan 1,4-β-xylosidase	[41,109,211]	[211]	*xlnD*	O00089	An01g09960	205670
3.2.1.37	Xylan 1,4-β-xylosidase					An02g00140	174379
3.2.1.37	Xylan 1,4-β-xylosidase					An08g01900	47677
3.2.1.55	α-L-arabinofuranosidase	[5,10,11,39,45, 47,49]	[77,186]	*abfA*	P42254	An01g00330	206387
3.2.1.55	α-L-arabinofuranosidase	[41,124]	[156]	*axhA*	P79019	An03g00960	55136
3.2.1.55	α-L-arabinofuranosidase					An08g01710	38549
3.2.1.55	α-L-arabinofuranosidase	[5,10,39,41,45, 46,48,49]		*abfB*	P42255	An15g02300	200605
3.2.1.55	α-L-arabinofuranosidase					An09g00880	131891
3.2.1.131	α-glucuronidase	[41,113]		*aguA*		An14g05800	56619
3.1.1.72	Acetylxylan esterase	[41]		*axeA*	A2QZI3	An09g01010	43522
3.1.1.73	Ferulic acid esterase A	[5,40,41,90,112, 116,118]	[90,118]	*faeA*	O42807	An09g00120	51662
3.1.1.73	Ferulic acid esterase B	[115,160]		*faeB*	Q8WZI8	N/A	51478
3.1.1.6	Acetyl esterase	[91]				An02g02540	N/A
3.2.1.23	β-galactosidase					An01g10350	46429
3.2.1.23	β-galactosidase					An06g00290	177434
3.2.1.23	β-galactosidase					An07g04420	180727
3.2.1.23	β-galactosidase					An14g05820	41910
3.2.1.23	β-galactosidase	[26,161]		*lacA*	P29853	An01g12150	51764

Table 10: Enzymatic activities required for the degradation of xyloglucan type XXGG by *A. niger*. For each isoenzyme, putative or characterized, is noted literature references and the gene ID in the sequencings of *A. niger* CBS 513.88 and ATCC 1015. The list of necessary enzymes is gathered from the work by [19,70,71].

EC number	Name	Gene characterization	Specificity	**Gene**	**Uniprot**	**CBS 513.88**	**ATCC 1015**
3.2.1.-	α-Xylosidase I	[170]				N/A	N/A
3.2.1.-	α-Xylosidase II	[171]				N/A	N/A
3.2.1.23	β-galactosidase					An01g10350	46429
3.2.1.23	β-galactosidase					An06g00290	177434
3.2.1.23	β-galactosidase					An07g04420	180727
3.2.1.23	β-galactosidase					An14g05820	41910
3.2.1.23	β-galactosidase	[26,161]		*lacA*	P29853	An01g12150	51764
3.2.1.4	endo-glucanase					An03g01050	194447
3.2.1.4	endo-glucanase					An03g05380	N/A
3.2.1.4	endo-glucanase					An03g05530	191511
3.2.1.4	endo-glucanase					An04g08550	194765
3.2.1.4	endo-glucanase					An08g01760	133986
3.2.1.4	endo-glucanase					An15g04900	182430
3.2.1.4	endo-glucanase	[41,139,140,150]	[134]	*eglA*	O74705	An14g02760	211053
3.2.1.4	endo-glucanase	[41,87,138,150]	[134]	*eglB*	O74706	An07g08950	209376
3.2.1.4	endo-glucanase	[134]	[134]	*eglC*	Q8TFP1	An01g01870	206333
3.2.1.151	xyloglucan-specific endo-β-1,4-glucanase	[169]	[169]	*xeg12A*	A1XP58	An01g03340	52011
3.2.1.55	exo-α-L-arabinofuranosidase (arabinoxylan arabinofuranohydrolase)	[5,11,39,45,47,49]	[77,186]	*abfA*	P42254	An01g00330	206387
3.2.1.55	exo-α-L-arabinofuranosidase (arabinoxylan arabinofuranohydrolase)					An08g01710	38549
3.2.1.55	exo-α-L-arabinofuranosidase (arabinoxylan arabinofuranohydrolase)	[5,10,39,41,45,46, 48,49]		*abfB*	P42255	An15g02300	200605
3.2.1.55	exo-α-L-arabinofuranosidase (arabinoxylan arabinofuranohydrolase)					An09g00880	131891
3.1.1.6	Acetyl esterase	[91]				An02g02540	N/A
3.2.1.37	Xylan 1,4-β-xylosidase	[41,109,211]		*xlnD*	O00089	An01g09960	205670
3.2.1.37	Xylan 1,4-β-xylosidase					An02g00140	174379

Continues on next page

Continued from last page

EC number	Name	Gene characterization	Specificity	**Gene**	**Uniprot**	**CBS 513.88**	**ATCC 1015**
3.2.1.37	Xylan 1,4-β-xylosidase					An08g01900	47677
3.2.1.21	β-glucosidase					An03g03740	213437
3.2.1.21	β-glucosidase					An03g05330	44520
3.2.1.21	β-glucosidase					An04g03170	131747
3.2.1.21	β-glucosidase					An06g02040	176601
3.2.1.21	β-glucosidase					An07g07630	139037
3.2.1.21	β-glucosidase					An07g09760	N/A
3.2.1.21	β-glucosidase					An08g08240	38077
3.2.1.21	β-glucosidase					An11g06080	208871
3.2.1.21	β-glucosidase					An11g06090	N/A
3.2.1.21	β-glucosidase					An14g01770	210981
3.2.1.21	β-glucosidase					An15g01890	182309
3.2.1.21	β-glucosidase					An15g04800	181816
3.2.1.21	β-glucosidase					An17g00520	129891
3.2.1.21	β-glucosidase	[30–36,137,223]		*bgl1*	A2RAL4	An18g03570	56782

Table 11: Enzymatic activities required for the degradation of xyloglucan type XXXG by *A. niger*. For each isoenzyme, putative or characterized, is noted literature references and the gene ID in the sequencings of *A. niger* CBS 513.88 and ATCC 1015. The list of necessary enzymes is gathered from the work by [19,70,71].

EC number	Name	Gene characterization	Specificity	**Gene**	**Uniprot**	**CBS 513.88**	**ATCC 1015**
3.2.1.-	α-Xylosidase I	[170]				N/A	N/A
3.2.1.-	α-Xylosidase II	[171]				N/A	N/A
3.2.1.23	β-galactosidase					An01g10350	46429
3.2.1.23	β-galactosidase					An06g00290	177434
3.2.1.23	β-galactosidase					An07g04420	180727
3.2.1.23	β-galactosidase					An14g05820	41910
3.2.1.23	β-galactosidase	[26,161]		*lacA*	P29853	An01g12150	51764
3.2.1.4	endo-glucanase					An03g01050	194447
3.2.1.4	endo-glucanase					An03g05380	N/A
3.2.1.4	endo-glucanase					An03g05530	191511
3.2.1.4	endo-glucanase					An04g08550	194765
3.2.1.4	endo-glucanase					An08g01760	133986
3.2.1.4	endo-glucanase					An15g04900	182430
3.2.1.4	endo-glucanase	[41,139,140,150]	[134]	*eglA*	O74705	An14g02760	211053
3.2.1.4	endo-glucanase	[41,87,138,150]	[134]	*eglB*	O74706	An07g08950	209376
3.2.1.4	endo-glucanase	[134]	[134]	*eglC*	Q8TFP1	An01g01870	206333
3.2.1.151	xyloglucan-specific endo-β-1,4-glucanase	[169]	[169]	*xeg12A*	A1XP58	An01g03340	52011
	1,2-α-L-fucosidase	[95]	[95]	*fucA*			
3.2.1.55	exo-α-L-arabinofuranosidase (arabinoxylan arabinofuranohydrolase)	[5,11,39,45,47,49]	[77,186]	*abfA*	P42254	An01g00330	206387
3.2.1.55	exo-α-L-arabinofuranosidase (arabinoxylan arabinofuranohydrolase)					An08g01710	38549
3.2.1.55	exo-α-L-arabinofuranosidase (arabinoxylan arabinofuranohydrolase)	[5,10,39,41,45,46,48,49]		*abfB*	P42255	An15g02300	200605
3.2.1.55	exo-α-L-arabinofuranosidase (arabinoxylan arabinofuranohydrolase)					An09g00880	131891
3.1.1.6	Acetyl esterase	[91]				An02g02540	N/A
3.2.1.37	Xylan 1,4-β-xylosidase	[41,109,211]		*xlnD*	O00089	An01g09960	205670
3.2.1.37	Xylan 1,4-β-xylosidase					An02g00140	174379
3.2.1.37	Xylan 1,4-β-xylosidase					An08g01900	47677
3.2.1.21	β-glucosidase					An03g03740	213437
3.2.1.21	β-glucosidase					An03g05330	44520
3.2.1.21	β-glucosidase					An04g03170	131747
3.2.1.21	β-glucosidase					An06g02040	176601
3.2.1.21	β-glucosidase					An07g07630	139037
3.2.1.21	β-glucosidase					An07g09760	N/A
3.2.1.21	β-glucosidase					An08g08240	38077
3.2.1.21	β-glucosidase					An11g06080	208871
3.2.1.21	β-glucosidase					An11g06090	N/A
3.2.1.21	β-glucosidase					An14g01770	210981
3.2.1.21	β-glucosidase					An15g01890	182309
3.2.1.21	β-glucosidase					An15g04800	181816
3.2.1.21	β-glucosidase					An17g00520	129891
3.2.1.21	β-glucosidase	[30–36,137,223]		*bgl1*	A2RAL4	An18g03570	56782
3.2.1.22	α-galactosidase	[24,26]	[24]	*aglC*	Q9UUZ4	An09g00260	212736
3.2.1.22	α-galactosidase					An01g01320	172232

Continues on next page

EC number	Name	Gene characterization	Specificity	Gene	Uniprot	CBS 513.88	ATCC 1015
3.2.1.22	α-galactosidase	[26,27]		*aglB*	Q9Y865	An02g11150	207264
3.2.1.22	α-galactosidase	[26,28,29]		*aglA*	A2QL72	An06g00170	37736
3.2.1.22	α-galactosidase					An11g06330	39180
3.2.1.22	α-galactosidase					An14g01800	185285

Continued from last page

Table 12: Enzymatic activities required for the degradation of xylogalacturonan by *A. niger*. For each isoenzyme, putative or characterized, is noted literature references and the gene ID in the sequencings of *A. niger* CBS 513.88 and ATCC 1015. Gene names in parentheses are genes that have not been characterized with classical molecular biological techniques, but identified from their expression profiles and sequence information. The list of necessary enzymes is gathered from the work by [19,64–67].

EC number	Name	Gene characterization	Specificity	Gene	Uniprot	CBS 513.88	ATCC 1015
4.2.2.2	pectate lyase A	[5,97]	[97]	*plyA*	Q9C2Z0	An10g00870	45021
4.2.2.10	pectin lyase	[5,133,141,164, 172,192]	[207]	*pelA*	Q01172	An14g04370	41815
4.2.2.10	pectin lyase					An15g07160	210387
4.2.2.10	pectin lyase	[5,131,133]		*pelD*	P22864	An19g00270	55212
4.2.2.10	pectin lyase					An11g04030	208760
4.2.2.10	pectin lyase	[5,163,215]		*pelB*	Q00205	An03g00190	45821
3.2.1.67	exo-poly-D-galacturonase	[6]		*(rgxA)*		An01g14650	172236
3.2.1.67	exo-poly-D-galacturonase	[6]		*(rgxB)*		An03g02080	194461
3.2.1.67	exo-poly-D-galacturonase	[6]		*(pgxB)*		An03g06740	191158
3.2.1.67	exo-poly-D-galacturonase	[6]		*(pgxA)*		An11g04040	178172
3.2.1.67	exo-poly-D-galacturonase	[5,6]		*(pgaX)*		An12g07500	42184
3.2.1.67	exo-poly-D-galacturonase	[6]		*(rgxC)*		An18g04810	42917
3.2.1.15	endo-Polygalacturonase	[5,104,212]	[98,179]	*pgaI*	P26213	An01g11520	141677
3.2.1.15	endo-Polygalacturonase	[5,182]	[98]	*pgaE*	O42809	An01g14670	46255
3.2.1.15	endo-Polygalacturonase	[5,181,224]	[181]	*pgaB*	P26214	An02g04900	52219
3.2.1.15	endo-Polygalacturonase			*(pgxC)*		An02g12450	172944
3.2.1.15	endo-Polygalacturonase	[5,106]	[98]	*pgaC*	Q12554	An05g02440	43957
3.2.1.15	endo-Polygalacturonase			*(rhgC)*		An06g02070	123651
3.2.1.15	endo-Polygalacturonase	[5,183]	[183]	*pgaD*	Q9P4W2	An09g03260	50161
3.2.1.15	endo-Polygalacturonase					An09g05920	N/A
3.2.1.15	endo-Polygalacturonase	[5,201]	[201]	*rhgA*	P87160	An12g00950	189722
3.2.1.15	endo-Polygalacturonase	[5,14,105–107, 147,180,194,213]	[98,165,166,179]	*pgaII*		An15g05370	182156
3.2.1.15	endo-Polygalacturonase	[5,181]	[181]	*pgaA*		An16g06990	214598
3.2.1.15	rhamnogalacturonase B	[5,201]	[201]	*rhgB*	P87161	An14g04200	211163
3.2.1.37	Xylan 1,4-β-xylosidase	[41,109,211]		*xlnD*	O00089	An01g09960	205670
3.2.1.37	Xylan 1,4-β-xylosidase					An02g00140	174379
3.2.1.37	Xylan 1,4-β-xylosidase					An08g01900	47677

Table 13: Enzymatic activities required for the degradation of arabinogalactan I by *A. niger*. For each isoenzyme, putative or characterized, is noted literature references and the gene ID in the sequencings of *A. niger* CBS 513.88 and ATCC 1015. The list of necessary enzymes is gathered from the work by [12,72,73].

EC number	Name	Gene characterization	Specificity	**Gene**	**Uniprot**	**CBS 513.88**	**ATCC 1015**
3.2.1.89	β-1,4-endogalactanase					An16g06590	N/A
3.2.1.89	β-1,4-endogalactanase	[12,221]	[12,221]	*galA*	Q8X168	An18g05940	187227
3.2.1.23	β-galactosidase					An01g10350	46429
3.2.1.23	β-galactosidase					An06g00290	177434
3.2.1.23	β-galactosidase					An07g04420	180727
3.2.1.23	β-galactosidase					An14g05820	41910
3.2.1.23	β-galactosidase	[26,161]		*lacA*	P29853	An01g12150	51764
3.2.1.55	exo-α-L-arabinofuranosidase	[5,10,11,39,45,47,49]	[77,186]	*abfA*	P42254	An01g00330	206387
3.2.1.55	exo-α-L-arabinofuranosidase					An08g01710	38549
3.2.1.55	exo-α-L-arabinofuranosidase	[5,10,39,41,45,46,48,49]	[77,78,193]	*abfB*	P42255	An15g02300	200605
3.2.1.55	exo-α-L-arabinofuranosidase					An09g00880	131891
3.2.1.99	endo-1,5-α-L-arabinanase					An02g01400	134398
3.2.1.99	endo-1,5-α-L-arabinanase					An02g10550	197735
3.2.1.99	endo-1,5-α-L-arabinanase	[5,45,49,119]		*abnA*	P42256	An09g01190	203143
3.2.1.99	endo-1,5-α-L-arabinanase					An16g02730	184195

Table 14: Enzymatic activities required for the degradation of arabinogalactan II by *A. niger*. For each isoenzyme, putative or characterized, is noted literature references and the gene ID in the sequencings of *A. niger* CBS 513.88 and ATCC 1015. The list of necessary enzymes is gathered from the work by [12,72,74,75].

EC number	Name	Gene characterization	Specificity	**Gene**	**Uniprot**	**CBS 513.88**	**ATCC 1015**
3.2.1.31	β-glucuronidase					An01g01260	46827
3.2.1.31	β-glucuronidase					An05g02410	189620
	endo-β-1,3-galactanase					N/A	N/A
	endo-β-1,6-galactanase					N/A	N/A
3.2.1.55	exo-α-L-arabinofuranosidase	[5,10,11,39,45,47,49]	[77,186]	*abfA*	P42254	An01g00330	206387
3.2.1.55	exo-α-L-arabinofuranosidase					An08g01710	38549
3.2.1.55	exo-α-L-arabinofuranosidase	[5,10,39,41,45,46,48,49]	[77,78,193]	*abfB*	P42255	An15g02300	200605
3.2.1.55	exo-α-L-arabinofuranosidase					An09g00880	131891
3.2.1.99	endo-1,5-α-L-arabinanase					An02g01400	134398
3.2.1.99	endo-1,5-α-L-arabinanase					An02g10550	197735
3.2.1.99	endo-1,5-α-L-arabinanase	[5,39,45,49,119]		*abnA*	P42256	An09g01190	203143
3.2.1.99	endo 1,5-α-L-arabinanase					An16g02730	184195
3.2.1.23	β-galactosidase					An01g10350	46429
3.2.1.23	β-galactosidase					An06g00290	177434
3.2.1.23	β-galactosidase					An07g04420	180727
3.2.1.23	β-galactosidase					An14g05820	41910
3.2.1.23	β-galactosidase	[26,161]		*lacA*	P29853	An01g12150	51764

Table 15: Enzymatic activities required for the degradation of arabinan by *A. niger*. For each isoenzyme, putative or characterized, is noted literature references and the gene ID in the sequencings of *A. niger* CBS 513.88 and ATCC 1015. The list of necessary enzymes is gathered from the work by [76,78].

EC number	Name	Gene characterization	Specificity	**Gene**	**Uniprot**	**CBS 513.88**	**ATCC 1015**
3.2.1.55	exo-α-L-arabinofuranosidase	[5,39,45,47,49]	[77,186]	*abfA*	P42254	An01g00330	206387
3.2.1.55	exo-α-L-arabinofuranosidase	[5,11,39,45,47,49]				An08g01710	38549
3.2.1.55	exo-α-L-arabinofuranosidase	[5,10,39,41,45,46, 48,49]		*abfB*	P42255	An15g02300	200605
3.2.1.55	exo-α-L-arabinofuranosidase					An09g00880	131891
3.2.1.99	endo-1,5-α-L-arabinanase					An02g01400	134398
3.2.1.99	endo-1,5-α-L-arabinanase					An02g10550	197735
3.2.1.99	endo-1,5-α-L-arabinanase	[5,39,45,49,119]		*abnA*	P42256	An09g01190	203143
3.2.1.99	endo-1,5-α-L-arabinanase					An16g02730	184195
none	exo-arabinase	[19]					N/A
3.1.1.73	Ferulic acid esterase A	[5,40,41,90,112, 116,118]	[90,118]	*faeA*	O42807	An09g00120	51662
3.1.1.73	Ferulic acid esterase B	[115,160]		*faeB*	Q8WZI8	N/A	51478

Table 16: Enzymatic activities required for the degradation of rhamnogalacturonan I by *A. niger*. For each isoenzyme, putative or characterized, is noted literature references and the gene ID in the sequencings of *A. niger* CBS 513.88 and ATCC 1015. Gene names in parentheses are genes that have not been characterized with classical molecular biological techniques, but identified from their expression profiles and sequence information. The list of necessary enzymes is gathered from the work by [19,79,80].

EC number	Name	Gene characterization	Specificity	**Gene**	**Uniprot**	**CBS 513.88**	**ATCC 1015**
No EC	rhamnogalacturonan hydrolase	[5,201]		*rglA*	Q8NJK5	An14g01130	210947
4.2.2.2	pectate lyase A	[5,97]	[97]	*plyA*	Q9C2Z0	An10g00870	45021
4.2.2.10	pectin lyase	[5,133,141,164,172,192]	[207]	*pelA*	Q01172	An14g04370	41815
4.2.2.10	pectin lyase					An15g07160	210387
4.2.2.10	pectin lyase	[5,131,133]		*pelD*	P22864	An19g00270	55212
4.2.2.10	pectin lyase					An11g04030	208760
4.2.2.10	pectin lyase	[5,163,215]		*pelB*	Q00205	An03g00190	45821
3.2.1.67	exo-poly-D-galacturonase	[6]		*(rgxA)*		An01g14650	172236
3.2.1.67	exo-poly-D-galacturonase	[6]		*(rgxB)*		An03g02080	194461
3.2.1.67	exo-poly-D-galacturonase	[6]		*(pgxB)*		An03g06740	191158
3.2.1.67	exo-poly-D-galacturonase	[6]		*(pgxA)*		An11g04040	178172
3.2.1.67	exo-poly-D-galacturonase	[5,6]		*(pgaX)*		An12g07500	42184
3.2.1.67	exo-poly-D-galacturonase	[6]		*(rgxC)*		An18g04810	42917
3.2.1.15	endo-Polygalacturonase	[5,104,212]	[98,179]	*pgaI*	P26213	An01g11520	141677
3.2.1.15	endo-Polygalacturonase	[5,182]	[98]	*pgaE*	O42809	An01g14670	46255
3.2.1.15	endo-Polygalacturonase	[5,181,224]	[181]	*pgaB*	P26214	An02g04900	52219
3.2.1.15	endo-Polygalacturonase			*(pgxC)*		An02g12450	172944
3.2.1.15	endo-Polygalacturonase	[5,106]	[98]	*pgaC*	Q12554	An05g02440	43957
3.2.1.15	endo-Polygalacturonase			*(rhgC)*		An06g02070	123651
3.2.1.15	endo-Polygalacturonase	[5,183]	[183]	*pgaD*	Q9P4W2	An09g03260	50161
3.2.1.15	endo-Polygalacturonase					An09g05920	N/A
3.2.1.15	endo-Polygalacturonase	[5,201]	[201]	*rhgA*	P87160	An12g00950	189722
3.2.1.15	endo-Polygalacturonase	[5,14,105–107,147,180, 194,213]	[98,165,166,179]	*pgaII*		An15g05370	182156
3.2.1.15	endo-Polygalacturonase	[5,181]	[181]	*pgaA*		An16g06990	214598
3.2.1.15	rhamnogalacturonase B	[5,201]	[201]	*rhgB*	P87161	An14g04200	211163
No EC	rhamnogalacturonan acetylesterase (*rgaeA*)	[5,19,114]		*rgaeA*	Q96VT8	An09g02160	189254
3.2.1.55	exo-α-L-arabinofuranosidase	[5,10,11,39,45,47,49]	[77,186]	*abfA*	P42254	An01g00330	206387
3.2.1.55	exo-α-L-arabinofuranosidase					An08g01710	38549
3.2.1.55	exo-α-L-arabinofuranosidase	[5,10,39,41,45,46,49]		*abfB*	P42255	An15g02300	200605
3.2.1.55	exo-α-L-arabinofuranosidase					An09g00880	131891
3.2.1.99	endo 1,5-α-L-arabinanase					An02g01400	134398
3.2.1.99	endo 1,5-α-L-arabinanase					An02g10550	197735
3.2.1.99	endo 1,5-α-L-arabinanase	[5,39,45,49,119]		*abnA*	P42256	An09g01190	203143
3.2.1.99	endo 1,5-α-L-arabinanase					An16g02730	184195
No EC	exo-arabinase	[19]				N/A	N/A
3.2.1.23	β-galactosidase					An01g10350	46429

Continues on next page

EC number	Name	Gene characterization	Specificity	**Gene**	**Uniprot**	**CBS 513.88**	**ATCC 1015**
		Continued from last page					
3.2.1.23	β-galactosidase					An06g00290	177434
3.2.1.23	β-galactosidase					An07g04420	180727
3.2.1.23	β-galactosidase					An14g05820	41910
3.2.1.23	β-galactosidase	[26,161]		*lacA*	P29853	An01g12150	51764
3.2.1.89	β-1,4-endogalactanase					An16g06590	N/A
3.2.1.89	β-1,4-endogalactanase	[12,221]	[12,221]	*galA*	Q8X168	An18g05940	187227
3.1.1.73	Ferulic acid esterase A	[5,40,41,90,112,116,118]	[90,118]	*faeA*	O42807	An09g00120	51662
3.1.1.73	Ferulic acid esterase B	[115,160]		*faeB*	Q8WZI8	N/A	51478

Table 17: Literature on *A. niger* carbohydrate-active enzymes that could not be correlated to a specific gene.

EC number	Name	Enzyme characterization	Specificity
3.1.1.11	pectin methylesterase		[206]
3.1.1.6	Acetyl esterase	[157,167]	[157]
3.1.1.73	cinnamate esterase	[96]	
3.2.1.-	Xylosidase	[170,171]	
3.2.1.131	α-glucuronidase	[153,204]	[153]
3.2.1.15	endo-Polygalacturonase	[110,149]	[82,149,199]
3.2.1.21	β-glucosidase	[84,89,123,205,219,220,222,225]	[205,123,219,220]
3.2.1.22	α-galactosidase	[83,85,145,200]	[145,200]
3.2.1.23	β-galactosidase	[102,129]	
3.2.1.3	glucoamylase	[122,184]	
3.2.1.31	β-glucuronidase	[128]	
3.2.1.37	Xylan 1,4-β-xylosidase	[108,142,189]	
3.2.1.4	endo-glucanase	[198,214]	
3.2.1.40	α-L-rhamnosidase	[162]	
3.2.1.55	α-L-arabinofuranosidase	[130,143,144,146,217]	
3.2.1.67	exo-poly-D-galacturonase	[135,187]	[82,135,166,190]
3.2.1.78	Endo-1,4-β-D-mannanase	[117,173,188]	[173]
3.2.1.8	Endo-(1,4)-β-xylanase	[99,111,120,121,126,127,142,174,195,196,216]	[99,120,174]
3.2.1.89	β-1,4-endogalactanase	[208]	[208]
3.2.1.91	Cellulose 1,4-β-cellobiosidase (Cellobiohydrolase)	[81,197]	
3.2.1.99	endo 1,5-α-L-arabinanase	[186]	
4.2.2.10	pectin lyase		[82,165]
No EC	rhamnogalacturonan acetylesterase	[77]	
No EC	exo-β-1,3-galactanase	[185]	[185]
No EC	endo-β-1,6-galactanase	[103]	

Table 18: Clustering results for genes coding for polysaccharide-acting enzymes. The first two columns are the gene IDs from the two *A. niger* genome sequencings. The genes are sorted according to clusters. A short description of the expression pattern is found in the Regulation column, while a summary term for the activity profile of the genes of the cluster is found in the Activity profile column (if one such existed

ATCC 1015	CBS 513.88	Cluster	Gene	Regulation	Activity profile
122069	An09g03110	1	α-amylase	Up on mono-sugars and starch	Galactomannan and insoluble galactoglucomannan degradation
123981	An11g07660	1	candidate membrane bound β-glycosidase	Up on mono-sugars and starch	Galactomannan and insoluble galactoglucomannan degradation
184195	An16g02730	1	endo 1,5-α-L-arabinanase	Up on mono-sugars and starch	Galactomannan and insoluble galactoglucomannan degradation
211595	An12g04610	1	candidate endo-1,4-glucanase	Up on mono-sugars and starch	Galactomannan and insoluble galactoglucomannan degradation
214608	An16g06800	1	candidate endoglucanase; C-terminal CBM1 module	Up on mono-sugars and starch	Galactomannan and insoluble galactoglucomannan degradation
38077	An08g08240	1	β-glucosidase	Up on mono-sugars and starch	Galactomannan and insoluble galactoglucomannan degradation
39180	An11g06330	1	α-galactosidase	Up on mono-sugars and starch	Galactomannan and insoluble galactoglucomannan degradation
40875	An15g07760	1	Endo-1,4-β-D-mannanase	Up on mono-sugars and starch	Galactomannan and insoluble galactoglucomannan degradation
44520	An03g05330	1	β-glucosidase	Up on mono-sugars and starch	Galactomannan and insoluble galactoglucomannan degradation
46827	An01g01260	1	β-glucuronidase	Up on mono-sugars and starch	Galactomannan and insoluble galactoglucomannan degradation
182162	An15g07800	2	α-amylase	Up on mono-sugars and starch	GH family 13.
191511	An03g05530	2	endo-glucanase	Up on mono-sugars and starch	GH family 13.
40878	An15g07810	2	candidate α-1,3-glucan synthase	Up on mono-sugars and starch	GH family 13.
54378	An09g03070	2	candidate α-1,3-glucan synthase	Up on mono-sugars and starch	GH family 13.
55204	An04g09890	2	membrane-bound α-1,3-glucan synthase (*agsA*);	Up on mono-sugars and starch	GH family 13.
128077	An07g04930	3	candidate membrane-bound α-glycosidase distantly related to endoarabinanases	Down on xylan	No clear profile
45304	An04g06930	3	α-amylase	Down on xylan	No clear profile
50378	An05g01320	3	Endo-1,4-β-D-mannanase	Down on xylan	No clear profile
53797	An14g02670	3	candidate endoglucanase; C-terminal CBM1 module	Down on xylan	No clear profile
178393	An11g06320	4	endo-rhamnogalacturonase	No clear regulation	No clear profile
180922	An07g01000	4	endo-rhamnogalacturonase	No clear regulation	No clear profile
210233	An15g04570	4	candidate β-glycosidase distantly related to endoglucanases; C-terminal CBM1 module	No clear regulation	No clear profile
52219	An02g04900	4	endo-Polygalacturonase (*pgaB*)	No clear regulation	No clear profile
119858	An01g10930	5	candidate α-glucosidase	Up on starch and glucose	Starch-degradation
140567	An11g03340	5	α-amylase	Up on starch and glucose	Starch-degradation
181816	An15g04800	5	β-glucosidase	Up on starch and glucose	Starch-degradation
213597	An03g06550	5	glucoamylase (exo-1,4-glucosidase/amyloglucosidase) (*glaA*)	Up on starch and glucose	Starch-degradation
214233	An04g06920	5	α-glucosidase (*aglA/aglU*)	Up on starch and glucose	Starch-degradation
206445	An02g00850	6	glucoamylase (exo-1,4-glucosidase/amyloglucosidase)	Up on glucose and starch	α and β-glucan degradation and the pectin-backbone

Continues on next page

Continued from last page

ATCC 1015	CBS 513.88	Cluster	Gene	Regulation	Gene profile
209376	An07g08950	6	endo-glucanase (*eglB*)	Up on glucose and starch	α and β-glucan degradation and the pectin-backbone
211163	An14g04200	6	rhamnogalacturonase B (*rhgB*)	Up on glucose and starch	α and β-glucan degradation and the pectin-backbone
214598	An16g06990	6	endo-Polygalacturonase (*pgaA*)	Up on glucose and starch	α and β-glucan degradation and the pectin-backbone
37060	An02g06950	6	glucoamylase (exo-1,4-glucosidase/amyloglucosidase)	Up on glucose and starch	α and β-glucan degradation and the pectin-backbone
45821	An03g00190	6	pectin lyase (*pelB*)	Up on glucose and starch	α and β-glucan degradation and the pectin-backbone
123651	An06g02070	7	endo-Polygalacturonase ((*rhgC*))	No clear regulation	Pectin-backbone degradation
182430	An15g04900	7	endo-glucanase	No clear regulation	Pectin-backbone degradation
208760	An11g04030	7	pectin lyase	No clear regulation	Pectin-backbone degradation
44977	An10g00290	7	candidate α-glycosidase related to bacterial α-L-rhamnosidases	No clear regulation	Pectin-backbone degradation
45021	An10g00870	7	pectate lyase A (*plyA*)	No clear regulation	Pectin-backbone degradation
134398	An02g01400	8	endo 1,5-α-L-arabinanase	Up on starch	Xylan-backbone degradation
141677	An01g11520	8	endo-Polygalacturonase (*pgaI*)	Up on starch	Xylan-backbone degradation
172587	An01g06630	8	β-mannosidase	Up on starch	Xylan-backbone degradation
176718	An08g09140	8	candidate α-glycosidase related to bacterial α-L-rhamnosidases	Up on starch	Xylan-backbone degradation
206333	An01g01870	8	endo-glucanase (*eglC*)	Up on starch	Xylan-backbone degradation
40261	An07g00350	8	α-glucosidase	Up on starch	Xylan-backbone degradation
52011	An01g03340	8	xyloglucan-specific endo-β-1,4-glucanase (*xeg12A*)	Up on starch	Xylan-backbone degradation
178172	An11g04040	9	exo-poly-D-galacturonase ((*pgxA*))	Up on starch	Xylan-backbone degradation
189911	An12g03070	9	candidate α-glycosidase	Up on starch	Xylan-backbone degradation
55212	An19g00270	9	pectin lyase (*pelD*)	Up on starch	Xylan-backbone degradation
133986	An08g01760	10	endo-glucanase	Up on xylan, moreso on xylose	Xylan-active and with two endo-glucanases
211053	An14g02760	10	endo-glucanase (*eglA*)	Up on xylan, moreso on xylose	Xylan-active and with two endo-glucanases
43784	An12g02540	10	candidate β-glycosidase related to endoglucanases	Up on xylan, moreso on xylose	Xylan-active and with two endo-glucanases
46290	An01g13610	10	candidate α-glycosidase related to α-amylases and maltohexaose-forming α-amylases	Up on xylan, moreso on xylose	Xylan-active and with two endo-glucanases
51662	An09g00120	10	Ferulic acid esterase A (*faeA*)	Up on xylan, moreso on xylose	Xylan-active and with two endo-glucanases
52071	An01g00780	10	Endo-(1,4)-β-xylanase (*xynB/xlnB/xylB*)	Up on xylan, moreso on xylose	Xylan-active and with two endo-glucanases
176601	An06g02040	11	β-glucosidase	Up on arabinan, arabinose, xylan and xylose	Xylan exo-activities
205670	An01g09960	11	Xylan 1,4-β-xylosidase (*xlnD*)	Up on arabinan, arabinose, xylan and xylose	Xylan exo-activities
47677	An08g01900	11	Xylan 1,4-β-xylosidase	Up on arabinan, arabinose, xylan and xylose	Xylan exo-activities
56619	An14g05800	11	α-glucuronidase (*aguA*)	Up on arabinan, arabinose, xylan and xylose	Xylan exo-activities
131891	An09g00880	12	α-L-arabinofuranosidase	Up on xylan	No clear profile
188489	An09g03100	12	α-amylase	Up on xylan	No clear profile
210947	An14g01130	12	rhamnogalacturonan hydrolase (*rglA*)	Up on xylan	No clear profile
212736	An09g00260	12	α-galactosidase (*aglC*)	Up on xylan	No clear profile
54490	An12g02220	12	Cellulose 1,4-β-cellobiosidase (Cellobiohydrolase)	Up on xylan	No clear profile
138876	An11g06540	13	β-mannosidase (*mndA*)	Up on xylan, arabinan and arabinose	Galactoglucomannan degradation and arabinofuranosidases
183088	An15g04550	13	Endo-(1,4)-β-xylanase	Up on xylan, arabinan and arabinose	Galactoglucomannan degradation and arabinofuranosidases

Continues on next page

Continued from last page

ATCC 1015	CBS 513.88	Cluster	Gene	Regulation	Gene profile
185285	An14g01800	13	α-galactosidase	Up on xylan, arabinan and arabinose	Galactoglucomannan degradation and arabinofuranosidases
194447	An03g01050	13	endo-glucanase	Up on xylan, arabinan and arabinose	Galactoglucomannan degradation and arabinofuranosidases
200605	An15g02300	13	α-L-arabinofuranosidase (*abfB*)	Up on xylan, arabinan and arabinose	Galactoglucomannan degradation and arabinofuranosidases
206387	An01g00330	13	α-L-arabinofuranosidase (*abfA*)	Up on xylan, arabinan and arabinose	Galactoglucomannan degradation and arabinofuranosidases
37673	An06g02460	13	candidate β-glycosidase distantly related to β-N-acetylglucosaminidases	Up on xylan, arabinan and arabinose	Galactoglucomannan degradation and arabinofuranosidases
43342	An09g03300	13	related to α-glycosidases	Up on xylan, arabinan and arabinose	Galactoglucomannan degradation and arabinofuranosidases
51764	An01g12150	13	β-galactosidase (*lacA*)	Up on xylan, arabinan and arabinose	Galactoglucomannan degradation and arabinofuranosidases
140573	An11g02100	14	candidate β-glycosidase; related to monolignol β-glucosidase	Up on xylan and arabinan	Removal of ferulic esters from arabinan and xylan
41703	An14g02920	14	related to rhamnogalacturonyl hydrolases	Up on xylan and arabinan	Removal of ferulic esters from arabinan and xylan
49940	An18g05620	14	candidate α-glycosidase	Up on xylan and arabinan	Removal of ferulic esters from arabinan and xylan
51478	An12g10390	14	Ferulic acid esterase B (*faeB*)	Up on xylan and arabinan	Removal of ferulic esters from arabinan and xylan
53702	An16g00540	14	candidate α-glycosidase distantly related to α-1,2-L-fucosidases	Up on xylan and arabinan	Removal of ferulic esters from arabinan and xylan
56782	An18g03570	14	β-glucosidase (*bgl1*)	Up on xylan and arabinan	Removal of ferulic esters from arabinan and xylan
122978	An08g10780	15	candidate β-glucosidase related to galactan 1,3-β-galactosidase; C-terminal CBM35 module	No clear regulation	No clear profile.
46065	An04g09700	15	endo-xylogalacturonan hydrolase	No clear regulation	No clear profile.
52928	An11g03200	15	endo-inulinase (*inuA/inuB*)	No clear regulation	No clear profile.
184037	An16g02760	16	candidate α-glycosidase distantly related to α-1,2-L-fucosidases	Up on arabinose and arabinan	No clear profile.
212893	An12g01850	16	candidate β-glycosidase related to bacterial β-mannosidases	Up on arabinose and arabinan	No clear profile.
38549	An08g01710	16	α-L-arabinofuranosidase	Up on arabinose and arabinan	No clear profile.
50161	An09g03260	16	endo-Polygalacturonase (*pgaD*)	Up on arabinose and arabinan	No clear profile.
52111	An02g00610	16	candidate β-glycosidase distantly related to β-glucuronidases	Up on arabinose and arabinan	No clear profile.
52452	An02g13240	16	candidate α-glycosidase related to α-glucosidases	Up on arabinose and arabinan	No clear profile.
182309	An15g01890	17	β-glucosidase	Up on arabinan	Cellulose-degradation
210716	An16g02100	17	candidate β-glycosidase	Up on arabinan	Cellulose-degradation
212915	An12g02450	17	candidate α-1,3-glucan synthase; N-terminal GH13 module and C-terminal GT5 module	Up on arabinan	Cellulose-degradation
45461	An16g09090	17	candidate membrane bound β-glycosidase related to β-N-acetylglucosaminidase	Up on arabinan	Cellulose-degradation
51773	An01g11660	17	Cellulose 1,4-β-cellobiosidase (Cellobiohydrolase) (*cbhB*)	Up on arabinan	Cellulose-degradation
57002	An12g02460	17	α-amylase	Up on arabinan	Cellulose-degradation

Continues on next page

Continued from last page

ATCC 1015	CBS 513.88	Cluster	Gene	Regulation	Gene profile
120104	An02g07590	18	candidate β-glycosidase related to β-N-acetylhexosaminidase	No clear regulation	No clear profile.
210387	An15g07160	18	pectin lyase	No clear regulation	No clear profile.
213437	An03g03740	18	β-glucosidase	No clear regulation	No clear profile.
52811	An08g01100	18	candidate β-glycosidase related to exo-1,3-β-glucanases	No clear regulation	No clear profile.
128654	An09g05880	19	α-glucosidase	Up on arabinan	Cellulose-degradation
129891	An17g00520	19	β-glucosidase	Up on arabinan	Cellulose-degradation
175759	An06g02060	19	candidate β-glycosidase related to glucan 1,3-β-glucosidase	Up on arabinan	Cellulose-degradation
180727	An07g04420	19	β-galactosidase	Up on arabinan	Cellulose-degradation
190816	An04g02700	19	candidate α-glycosidase distantly related to plants alkaline α-galactosidases	Up on arabinan	Cellulose-degradation
194765	An04g08550	19	endo-glucanase	Up on arabinan	Cellulose-degradation
211162	An14g04190	19	candidate 1,4-α-glucan branching enzyme	Up on arabinan	Cellulose-degradation
39337	An11g08700	19	endo-rhamnogalacturonase	Up on arabinan	Cellulose-degradation
46621	An01g06120	19	candidate α-glycosidase related to glycogen-debranching enzyme	Up on arabinan	Cellulose-degradation
53159	An07g09330	19	Cellulose 1,4-β-cellobiosidase (Cellobiohydrolase) (*cbhA*)	Up on arabinan	Cellulose-degradation
205580	An01g11670	20	candidate endoglucanase; C-terminal CBM1 module	Up on starch and arabinan	No clear profile.
41815	An14g04370	20	pectin lyase (*pelA*)	Up on starch and arabinan	No clear profile.
44822	An13g02110	20	candidate α-glycosidase related to mammalian α-L-fucosidases	Up on starch and arabinan	No clear profile.
56664	An12g08280	20	exo-inulinase (*inuE/inuF/inu1*)	Up on starch and arabinan	No clear profile.
171269	An01g14600	21	Endo-(1,4)-β-xylanase	Up on arabinan	Xylan- and pectin-backbone activitities
182100	An15g03550	21	related to β-glycosidases	Up on arabinan	Xylan- and pectin-backbone activitities
182156	An15g05370	21	endo-Polygalacturonase (*pgaII*)	Up on arabinan	Xylan- and pectin-backbone activitities
189722	An12g00950	21	endo-Polygalacturonase (*rhgA*)	Up on arabinan	Xylan- and pectin-backbone activitities
43522	An09g01010	21	Acetylxylan esterase (*axeA*)	Up on arabinan	Xylan- and pectin-backbone activitities
52688	An08g05230	21	candidate endoglucanase	Up on arabinan	Xylan- and pectin-backbone activitities
187227	An18g05940	22	β-1,4-endogalactanase (*galA*)	Up on arabinan	No clear profile
194461	An03g02080	22	exo-poly-D-galacturonase ((*rgxB*))	Up on arabinan	No clear profile
197735	An02g10550	22	endo 1,5-α-L-arabinanase	Up on arabinan	No clear profile
202490	An18g04100	22	candidate glucan 1,3-β-glucosidase	Up on arabinan	No clear profile
38924	An11g03120	22	Endo-(1,4)-β-xylanase	Up on arabinan	No clear profile
41877	An14g05340	22	distantly related to rhamnogalacturonyl hydrolases	Up on arabinan	No clear profile
50927	An13g03710	22	α-glucosidase	Up on arabinan	No clear profile
170172	An01g06620	23	candidate α-L-rhamnosidase	Up on arabinan	Exo-α- and β- galactosidase and glucosidase
172232	An01g01320	23	α-galactosidase	Up on arabinan	Exo-α- and β- galactosidase and glucosidase
189620	An05g02410	23	β-glucuronidase	Up on arabinan	Exo-α- and β- galactosidase and glucosidase
207264	An02g11150	23	α-galactosidase (*aglB*)	Up on arabinan	Exo-α- and β- galactosidase and glucosidase
210981	An14g01770	23	β-glucosidase	Up on arabinan	Exo-α- and β- galactosidase and glucosidase
214857	An04g09690	23	pectin methylesterase	Up on arabinan	Exo-α- and β- galactosidase and glucosidase
37736	An06g00170	23	α-galactosidase (*aglA*)	Up on arabinan	Exo-α- and β- galactosidase and glucosidase
41910	An14g05820	23	β-galactosidase	Up on arabinan	Exo-α- and β- galactosidase and glucosidase
46429	An01g10350	23	β-galactosidase	Up on arabinan	Exo-α- and β- galactosidase and glucosidase
51410	An04g09070	23	candidate α-L-rhamnosidase	Up on arabinan	Exo-α- and β- galactosidase and glucosidase

Continues on next page

Continued from last page

ATCC 1015	CBS 513.88	Cluster	Gene	Regulation	Gene profile
131668	An12g05700	24	related to α-L-rhamnosidases	Up on arabinan	Rhamnogalacturonan I, smooth pectin and xylogalacturonan degradation
131747	An04g03170	24	β-glucosidase	Up on arabinan	Rhamnogalacturonan I, smooth pectin and xylogalacturonan degradation
139037	An07g07630	24	β-glucosidase	Up on arabinan	Rhamnogalacturonan I, smooth pectin and xylogalacturonan degradation
172236	An01g14650	24	exo-poly-D-galacturonase ((*rgxA*))	Up on arabinan	Rhamnogalacturonan I, smooth pectin and xylogalacturonan degradation
172944	An02g12450	24	endo-Polygalacturonase ((*pgxC*))	Up on arabinan	Rhamnogalacturonan I, smooth pectin and xylogalacturonan degradation
174379	An02g00140	24	Xylan 1,4-β-xylosidase	Up on arabinan	Rhamnogalacturonan I, smooth pectin and xylogalacturonan degradation
176039	An06g02420	24	candidate β-glycosidase	Up on arabinan	Rhamnogalacturonan I, smooth pectin and xylogalacturonan degradation
177434	An06g00290	24	β-galactosidase	Up on arabinan	Rhamnogalacturonan I, smooth pectin and xylogalacturonan degradation
179265	An11g00200	24	candidate β-glucosidase	Up on arabinan	Rhamnogalacturonan I, smooth pectin and xylogalacturonan degradation
189254	An09g02160	24	rhamnogalacturonan acetylesterase (*rgaeA*)	Up on arabinan	Rhamnogalacturonan I, smooth pectin and xylogalacturonan degradation
191158	An03g06740	24	exo-poly-D-galacturonase ((*pgxB*))	Up on arabinan	Rhamnogalacturonan I, smooth pectin and xylogalacturonan degradation
198063	An08g11070	24	candidate invertase	Up on arabinan	Rhamnogalacturonan I, smooth pectin and xylogalacturonan degradation
203143	An09g01190	24	endo 1,5-α-L-arabinanase (*abnA*)	Up on arabinan	Rhamnogalacturonan I, smooth pectin and xylogalacturonan degradation
208871	An11g06080	24	β-glucosidase	Up on arabinan	Rhamnogalacturonan I, smooth pectin and xylogalacturonan degradation
36414	An01g01340	24	candidate glycosidase related to a bacterial d-4,5 unsaturated glucuronyl hydrolase	Up on arabinan	Rhamnogalacturonan I, smooth pectin and xylogalacturonan degradation
40264	An07g00240	24	candidate α-glycosidase related to bacterial α-L-rhamnosidases	Up on arabinan	Rhamnogalacturonan I, smooth pectin and xylogalacturonan degradation
42184	An12g07500	24	exo-poly-D-galacturonase ((*pgaX*))	Up on arabinan	Rhamnogalacturonan I, smooth pectin and xylogalacturonan degradation
42916	An18g04800	24	candidate membrane-bound α-glycosidase related to α-L-rhamnosidases	Up on arabinan	Rhamnogalacturonan I, smooth pectin and xylogalacturonan degradation
42917	An18g04810	24	exo-poly-D-galacturonase ((*rgxC*))	Up on arabinan	Rhamnogalacturonan I, smooth pectin and xylogalacturonan degradation
43957	An05g02440	24	endo-Polygalacturonase (*pgaC*)	Up on arabinan	Rhamnogalacturonan I, smooth pectin and xylogalacturonan degradation
44585	An03g06310	24	pectin methylesterase (*pmeA*)	Up on arabinan	Rhamnogalacturonan I, smooth pectin and xylogalacturonan degradation
46255	An01g14670	24	endo-Polygalacturonase (*pgaE*)	Up on arabinan	Rhamnogalacturonan I, smooth pectin and xylogalacturonan degradation
50997	An17g00300	24	candidate β-glycosydase	Up on arabinan	Rhamnogalacturonan I, smooth pectin and xylogalacturonan degradation
55136	An03g00960	24	α-L-arabinofuranosidase (*axhA*)	Up on arabinan	Rhamnogalacturonan I, smooth pectin and xylogalacturonan degradation
55419	An01g04880	24	α-glucosidase	Up on arabinan	Rhamnogalacturonan I, smooth pectin and xylogalacturonan degradation

Continues on next page

Continued from last page

ATCC 1015	CBS 513.88	Cluster	Gene	Regulation	Gene profile
57436	An03g00940	24	Endo-(1,4)-β-xylanase (*xynA*)	Up on arabinan	Rhamnogalacturonan I, smooth pectin and xylogalacturonan degradation

Table 19: Clustering results for significantly regulated genes coding for polysaccharide-acting enzymes. The first two columns are the gene IDs from the two genome sequencings. The genes are sorted according to clusters. The “Similar to”-column refers to which cluster of App. Table 18/Figure **??** the cluster in this table is similar to. A short description of the expression pattern is found in the Regulation column, while a summary term for the activity profile of the genes of the cluster is found in the Activity profile column (if one such existed

ATCC 1015	CBS 513.88	Cluster	Similar to	Gene	Regulation	Gene profile
140573	An11g02100	1	14	candidate β-glycosidase; related to monolignol β-glucosidase	Removal of ferulic esters from arabinan and xylan	Up on xylan and arabinan
41703	An14g02920	1	14	related to rhamnogalacturonyl hydrolases	Removal of ferulic esters from arabinan and xylan	Up on xylan and arabinan
51478	An12g10390	1	14	Ferulic acid esterase B (*faeB*)	Removal of ferulic esters from arabinan and xylan	Up on xylan and arabinan
53702	An16g00540	1	14	candidate α-glycosidase distantly related to α-1,2-L-fucosidases	Removal of ferulic esters from arabinan and xylan	Up on xylan and arabinan
56782	An18g03570	1	14	β-glucosidase (*bgl1*)	Removal of ferulic esters from arabinan and xylan	Up on xylan and arabinan
184037	An16g02760	2	16	candidate α-glycosidase distantly related to α-1,2-L-fucosidases	No clear profile.	Up on arabinose and arabinan
212893	An12g01850	2	16	candidate β-glycosidase related to bacterial β-mannosidases	No clear profile.	Up on arabinose and arabinan
38549	An08g01710	2	16	α-L-arabinofuranosidase	No clear profile.	Up on arabinose and arabinan
50161	An09g03260	2	16	endo-Polygalacturonase (*pgaD*)	No clear profile.	Up on arabinose and arabinan
122978	An08g10780	3	15	candidate β-glucosidase related to galactan 1,3-b-galactosidase; C-terminal CBM35 module	No clear profile.	No clear regulation
46065	An04g09700	3	15	endo-xylogalacturonan hydrolase	No clear profile.	No clear regulation
185285	An14g01800	4	13	α-galactosidase	Galactoglucomannan degradation and arabinofuranosidases	Up on xylan, arabinan and arabinose
194447	An03g01050	4	13	endo-glucanase	Galactoglucomannan degradation and arabinofuranosidases	Up on xylan, arabinan and arabinose
200605	An15g02300	4	13	α-L-arabinofuranosidase (*abfB*)	Galactoglucomannan degradation and arabinofuranosidases	Up on xylan, arabinan and arabinose
206387	An01g00330	4	13	α-L-arabinofuranosidase (*abfA*)	Galactoglucomannan degradation and arabinofuranosidases	Up on xylan, arabinan and arabinose
51764	An01g12150	4	13	β-galactosidase (*lacA*)	Galactoglucomannan degradation and arabinofuranosidases	Up on xylan, arabinan and arabinose
138876	An11g06540	5	13	β-mannosidase (*mndA*)	No clear profile	Up on xylan, arabinan and arabinose
183088	An15g04550	5	13	Endo-(1,4)-β-xylanase	No clear profile	Up on xylan, arabinan and arabinose
188489	An09g03100	6	11	α-amylase	Xylan exo-activities	Up on arabinan, arabinose, xylan and xylose
205670	An01g09960	6	11	Xylan 1,4-β-xylosidase (*xlnD*)	Xylan exo-activities	Up on arabinan, arabinose, xylan and xylose
43342	An09g03300	6	11	related to α-glycosidases	Xylan exo-activities	Up on arabinan, arabinose, xylan and xylose
47677	An08g01900	6	11	Xylan 1,4-β-xylosidase	Xylan exo-activities	Up on arabinan, arabinose, xylan and xylose
56619	An14g05800	6	11	α-glucuronidase (*aguA*)	Xylan exo-activities	Up on arabinan, arabinose, xylan and xylose
211053	An14g02760	7	10	endo-glucanase (*eglA*)	Xylan-active enzymes	Up on xylan, moreso on xylose
46290	An01g13610	7	10	candidate α-glycosidase related to α-amylases and maltohexaose-forming α-amylases	Xylan-active enzymes	Up on xylan, moreso on xylose
51662	An09g00120	7	10	Ferulic acid esterase A (*faeA*)	Xylan-active enzymes	Up on xylan, moreso on xylose
52071	An01g00780	7	10	Endo-(1,4)-β-xylanase (*xynB/xlnB/xylB*)	Xylan-active enzymes	Up on xylan, moreso on xylose
122069	An09g03110	8	1	α-amylase	No clear profile.	Arabinan down and Xylose up
184195	An16g02730	8	1	endo 1,5-α-L-arabinanase	No clear profile.	Arabinan down and Xylose up

Continues on next page

Continued from last page

ATCC 1015	CBS 513.88	Cluster	Similar to	Gene	Regulation	Gene profile
214608	An16g06800	8	1	candidate endoglucanase; C-terminal CBM1 module	No clear profile.	Arabinan down and Xylose up
44520	An03g05330	8	1	β-glucosidase	No clear profile.	Arabinan down and Xylose up
191511	An03g05530	9	4 and 6	endo-glucanase	Endo-acting enzymes	Induced by high sugar-concentrations
210233	An15g04570	9	4 and 6	candidate β-glycosidase distantly related to endoglucanases; C-terminal CBM1 module	Endo-acting enzymes	Induced by high sugar-concentrations
211163	An14g04200	9	4 and 6	rhamnogalacturonase B (*rhgB*)	Endo-acting enzymes	Induced by high sugar-concentrations
214598	An16g06990	9	4 and 6	endo-Polygalacturonase (*pgaA*)	Endo-acting enzymes	Induced by high sugar-concentrations
52219	An02g04900	9	4 and 6	endo-Polygalacturonase (*pgaB*)	Endo-acting enzymes	Induced by high sugar-concentrations
55204	An04g09890	9	4 and 6	membrane-bound α-1,3-glucan synthase (EC 2.4.1.183); N-terminal GH13 module and C-terminal GT5 module	Endo-acting enzymes	Induced by high sugar-concentrations
206445	An02g00850	10	3 and 6	glucoamylase (exo-1,4-glucosidase/amyloglucosidase)	Amylases and pectin lyase	Up on glucose and starch, down on xylan
45304	An04g06930	10	3 and 6	α-amylase	Amylases and pectin lyase	Up on glucose and starch, down on xylan
45821	An03g00190	10	3 and 6	pectin lyase (*pelB*)	Amylases and pectin lyase	Up on glucose and starch, down on xylan
119858	An01g10930	11	5	candidate α-glucosidase	Starch-degradation	Up on starch and glucose
140567	An11g03340	11	5	α-amylase	Starch-degradation	Up on starch and glucose
213597	An03g06550	11	5	glucoamylase (exo-1,4-glucosidase/amyloglucosidase) (*glaA*)	Starch-degradation	Up on starch and glucose
214233	An04g06920	11	5	α-glucosidase (*aglA/aglU*)	Starch-degradation	Up on starch and glucose
120104	An02g07590	12	18	candidate β-glycosidase related to β-N-acetylhexosaminidase	No clear profile.	No clear regulation
210387	An15g07160	12	18	pectin lyase	No clear profile.	No clear regulation
54378	An09g03070	12	18	candidate α-1,3-glucan synthase; N-terminal GH13 module and C-terminal GT5 module	No clear profile.	No clear regulation
41815	An14g04370	13	20	pectin lyase (*pelA*)	Pectin lyases and glucanases/glucosidases	Up on starch and arabinan
52011	An01g03340	13	20	xyloglucan-specific endo-β-1,4-glucanase (*xeg12A*)	Pectin lyases and glucanases/glucosidases	Up on starch and arabinan
56664	An12g08280	13	20	exo-inulinase (*inuE/inuF/inu1*)	Pectin lyases and glucanases/glucosidases	Up on starch and arabinan
128654	An09g05880	14	19	α-glucosidase	Glucans and glucosidases	Up on arabinan and glucose
129891	An17g00520	14	19	β-glucosidase	Glucans and glucosidases	Up on arabinan and glucose
190816	An04g02700	14	19	candidate α-glycosidase distantly related to plants alkaline α-galactosidases	Glucans and glucosidases	Up on arabinan and glucose
194765	An04g08550	14	19	endo-glucanase	Glucans and glucosidases	Up on arabinan and glucose
211162	An14g04190	14	19	candidate 1,4-a-glucan branching enzyme	Glucans and glucosidases	Up on arabinan and glucose
53159	An07g09330	14	19	Cellulose 1,4-β-cellobiosidase (Cellobiohydrolase) (*cbhA*)	Glucans and glucosidases	Up on arabinan and glucose
39337	An11g08700	15	19	endo-rhamnogalacturonase	No clear profile	Up on arabinan
46621	An01g06120	15	19	candidate α-glycosidase related to glycogen-debranching enzyme	No clear profile	Up on arabinan
170172	An01g06620	16	17 and 23	candidate α-L-rhamnosidase	Cellulose-degradation and rhamnosidases	Up on arabinan
187227	An18g05940	16	17 and 23	β-1,4-endogalactanase (*galA*)	Cellulose-degradation and rhamnosidases	Up on arabinan
210716	An16g02100	16	17 and 23	candidate β-glycosidase	Cellulose-degradation and rhamnosidases	Up on arabinan
51410	An04g09070	16	17 and 23	candidate α-L-rhamnosidase	Cellulose-degradation and rhamnosidases	Up on arabinan

Continues on next page

Continued from last page

ATCC 1015	CBS 513.88	Cluster	Similar to	Gene	Regulation	Gene profile
51773	An01g11660	16	17 and 23	Cellulose 1,4-β-cellobiosidase (Cellobiohydrolase) (*cbhB*)	Cellulose-degradation and rhamnosidases	Up on arabinan
182156	An15g05370	17	21-22	endo-Polygalacturonase (*pgaII*)	Xylan- and pectin-backbone activities	Up on arabinan
189722	An12g00950	17	21-22	endo-Polygalacturonase (*rhgA*)	Xylan- and pectin-backbone activities	Up on arabinan
194461	An03g02080	17	21-22	exo-poly-D-galacturonase ((*rgxB*))	Xylan- and pectin-backbone activities	Up on arabinan
197735	An02g10550	17	21-22	endo 1,5-α-L-arabinanase	Xylan- and pectin-backbone activities	Up on arabinan
202490	An18g04100	17	21-22	candidate glucan 1,3-β-glucosidase	Xylan- and pectin-backbone activities	Up on arabinan
38924	An11g03120	17	21-22	Endo-(1,4)-β-xylanase	Xylan- and pectin-backbone activities	Up on arabinan
41877	An14g05340	17	21-22	distantly related to rhamnogalacturonyl hydrolases	Xylan- and pectin-backbone activities	Up on arabinan
44585	An03g06310	17	21-22	pectin methylesterase (*pmeA*)	Xylan- and pectin-backbone activities	Up on arabinan
50927	An13g03710	17	21-22	α-glucosidase	Xylan- and pectin-backbone activities	Up on arabinan
52688	An08g05230	17	21-22	candidate endoglucanase	Xylan- and pectin-backbone activities	Up on arabinan
207264	An02g11150	18	23	α-galactosidase (*aglB*)	No clear profile	Up on arabinan
214857	An04g09690	18	23	pectin methylesterase	No clear profile	Up on arabinan
172232	An01g01320	19	23	α-galactosidase	Exo-α- and β-galactosidase and α- and β-glucosidase	Up on arabinan
189620	An05g02410	19	23	β-glucuronidase	Exo-α- and β-galactosidase and α- and β-glucosidase	Up on arabinan
210981	An14g01770	19	23	β-glucosidase	Exo-α- and β-galactosidase and α- and β-glucosidase	Up on arabinan
37736	An06g00170	19	23	α-galactosidase (*aglA*)	Exo-α- and β-galactosidase and α- and β-glucosidase	Up on arabinan
41910	An14g05820	19	23	β-galactosidase	Exo-α- and β-galactosidase and α- and β-glucosidase	Up on arabinan
46429	An01g10350	19	23	β-galactosidase	Exo-α- and β-galactosidase and α- and β-glucosidase	Up on arabinan
131668	An12g05700	20	24	related to α-L-rhamnosidases	Rhamnogalacturonan I, smooth pectin and xylogalacturonan degradation	Up on arabinan
131747	An04g03170	20	24	β-glucosidase	Rhamnogalacturonan I, smooth pectin and xylogalacturonan degradation	Up on arabinan
172236	An01g14650	20	24	exo-poly-D-galacturonase ((*rgxA*))	Rhamnogalacturonan I, smooth pectin and xylogalacturonan degradation	Up on arabinan
172944	An02g12450	20	24	endo-Polygalacturonase ((*pgxC*))	Rhamnogalacturonan I, smooth pectin and xylogalacturonan degradation	Up on arabinan
174379	An02g00140	20	24	Xylan 1,4-β-xylosidase	Rhamnogalacturonan I, smooth pectin and xylogalacturonan degradation	Up on arabinan

Continues on next page

Continued from last page

ATCC 1015	CBS 513.88	Cluster	Similar to	Gene	Regulation	Gene profile
177434	An06g00290	20	24	β-galactosidase	Rhamnogalacturonan I, smooth pectin and xylogalacturonan degradation	Up on arabinan
179265	An11g00200	20	24	candidate β-glucosidase	Rhamnogalacturonan I, smooth pectin and xylogalacturonan degradation	Up on arabinan
189254	An09g02160	20	24	rhamnogalacturonan acetylesterase (*rgaeA*)	Rhamnogalacturonan I, smooth pectin and xylogalacturonan degradation	Up on arabinan
191158	An03g06740	20	24	exo-poly-D-galacturonase ((*pgxB*))	Rhamnogalacturonan I, smooth pectin and xylogalacturonan degradation	Up on arabinan
198063	An08g11070	20	24	candidate invertase	Rhamnogalacturonan I, smooth pectin and xylogalacturonan degradation	Up on arabinan
203143	An09g01190	20	24	endo 1,5-α-L-arabinanase (*abnA*)	Rhamnogalacturonan I, smooth pectin and xylogalacturonan degradation	Up on arabinan
208871	An11g06080	20	24	β-glucosidase	Rhamnogalacturonan I, smooth pectin and xylogalacturonan degradation	Up on arabinan
36414	An01g01340	20	24	candidate glycosidase related to a bacterial d-4,5 unsaturated glucuronyl hydrolase	Rhamnogalacturonan I, smooth pectin and xylogalacturonan degradation	Up on arabinan
42184	An12g07500	20	24	exo-poly-D-galacturonase ((*pgaX*))	Rhamnogalacturonan I, smooth pectin and xylogalacturonan degradation	Up on arabinan
42916	An18g04800	20	24	candidate membrane-bound α-glycosidase related to α-L-rhamnosidases	Rhamnogalacturonan I, smooth pectin and xylogalacturonan degradation	Up on arabinan
42917	An18g04810	20	24	exo-poly-D-galacturonase ((*rgxC*))	Rhamnogalacturonan I, smooth pectin and xylogalacturonan degradation	Up on arabinan
43957	An05g02440	20	24	endo-Polygalacturonase (*pgaC*)	Rhamnogalacturonan I, smooth pectin and xylogalacturonan degradation	Up on arabinan
46255	An01g14670	20	24	endo-Polygalacturonase (*pgaE*)	Rhamnogalacturonan I, smooth pectin and xylogalacturonan degradation	Up on arabinan
50997	An17g00300	20	24	candidate β-glycosidase	Rhamnogalacturonan I, smooth pectin and xylogalacturonan degradation	Up on arabinan
55136	An03g00960	20	24	α-L-arabinofuranosidase (*axhA*)	Rhamnogalacturonan I, smooth pectin and xylogalacturonan degradation	Up on arabinan
55419	An01g04880	20	24	α-glucosidase	Rhamnogalacturonan I, smooth pectin and xylogalacturonan degradation	Up on arabinan
57436	An03g00940	20	24	Endo-(1,4)-β-xylanase (*xynA*)	Rhamnogalacturonan I, smooth pectin and xylogalacturonan degradation	Up on arabinan
